# Anionic Magnesium and Calcium Hydrides: Transforming CO into Unsaturated Disilyl Ethers

**DOI:** 10.1002/anie.202215218

**Published:** 2022-11-29

**Authors:** Jacob S. McMullen, Ryan Huo, Petra Vasko, Alison J. Edwards, Jamie Hicks

**Affiliations:** ^1^ Research School of Chemistry Australian National University Acton ACT 2601 Australia; ^2^ Department of Chemistry University of Helsinki A.I. Virtasen aukio 1, P.O. Box 55 00014 Helsinki Finland; ^3^ Australian Centre for Neutron Scattering Australian Nuclear Science and Technology Organisation Sydney NSW 2234 Australia

**Keywords:** Carbon Monoxide, Group 2, Hydrides, Main Group Elements, Neutron Diffraction

## Abstract

The synthesis, characterisation and reactivity of two isostructural anionic magnesium and calcium complexes is reported. By X‐ray and neutron diffraction techniques, the anionic hydrides are shown to exist as dimers, held together by a range of interactions between the two anions and two bridging potassium cations. Unlike the vast proportion of previously reported dimeric group 2 hydrides, which have hydrides that bridge two group 2 centres, here the hydrides are shown to be “terminal”, but stabilised by interactions with the potassium cations. Both anionic hydrides were found to insert and couple CO under mild reaction conditions to give the corresponding group 2 *cis*‐ethenediolate complexes. These *cis*‐ethenediolate complexes were found to undergo salt elimination reactions with silyl chlorides, allowing access to small unsaturated disilyl ethers with a high percentage of their mass originating from the C_1_ source CO.

## Graphical abstract

CO has directly been transformed into small organic starting materials using earth‐abundant, anionic group 2 hydride reagents.

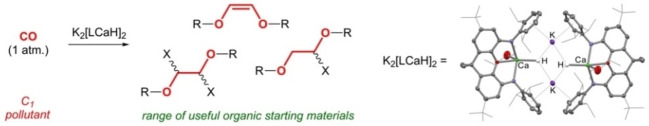

Small C_1_ molecules such as CO and CO_2_ are often regarded as environmental pollutants that are common waste products in numerous industrial and biological processes.^[1]^ To a synthetic chemist however, these small molecules can be viewed as sustainable, inexpensive feedstocks for the synthesis of high‐value commodity chemicals.^[2]^ As CO can now be sustainably sourced from biomass,^[3]^ it now appears to be an underutilised starting material in chemical synthesis. That said, the idea of using CO in the synthesis of useful molecules is not new; Franz Fischer and Hans Tropsch developed the method for synthesising mixtures of long‐chain hydrocarbons directly from Syngas (CO+H_2_) almost a century ago.^[4]^ However, problems in controlling exact chain lengths, as well as losing >50 % by mass in the by‐product H_2_O, still remain an issue to this day.[^4^, ^5^]

Recently, a number of systems have been reported to homologate CO in controllable ways, which involve both C−C and C−O coupling.^[6]^ Many of these systems are low‐oxidation state main group species, which reductively couple CO to give fragments of the type (C_*n*_O_*n*_)^*m*−^ (*n*=2–6, *m*=2–6), with or without the aid of a transition metal carbonyl catalyst. Examples include two recent studies by Crimmin and co‐workers,[^7^, ^8^] who have shown controllable step‐wise growth of CO chains using an Al^I^ reagent in combination with various transition metal carbonyl fragments (e.g. Figure 1, **I**); the hexamerisation of CO with a magnesium(I) complex by Jones and co‐workers (**II**);^[9]^ the reductive coupling of CO by an acyclic silylene by Aldridge and co‐workers (**III**);^[10]^ and the reductive coupling of 4 molecules of CO by a single diboryne (**IV**),^[11]^ amongst a handful of others.^[12]^

**Figure 1:**
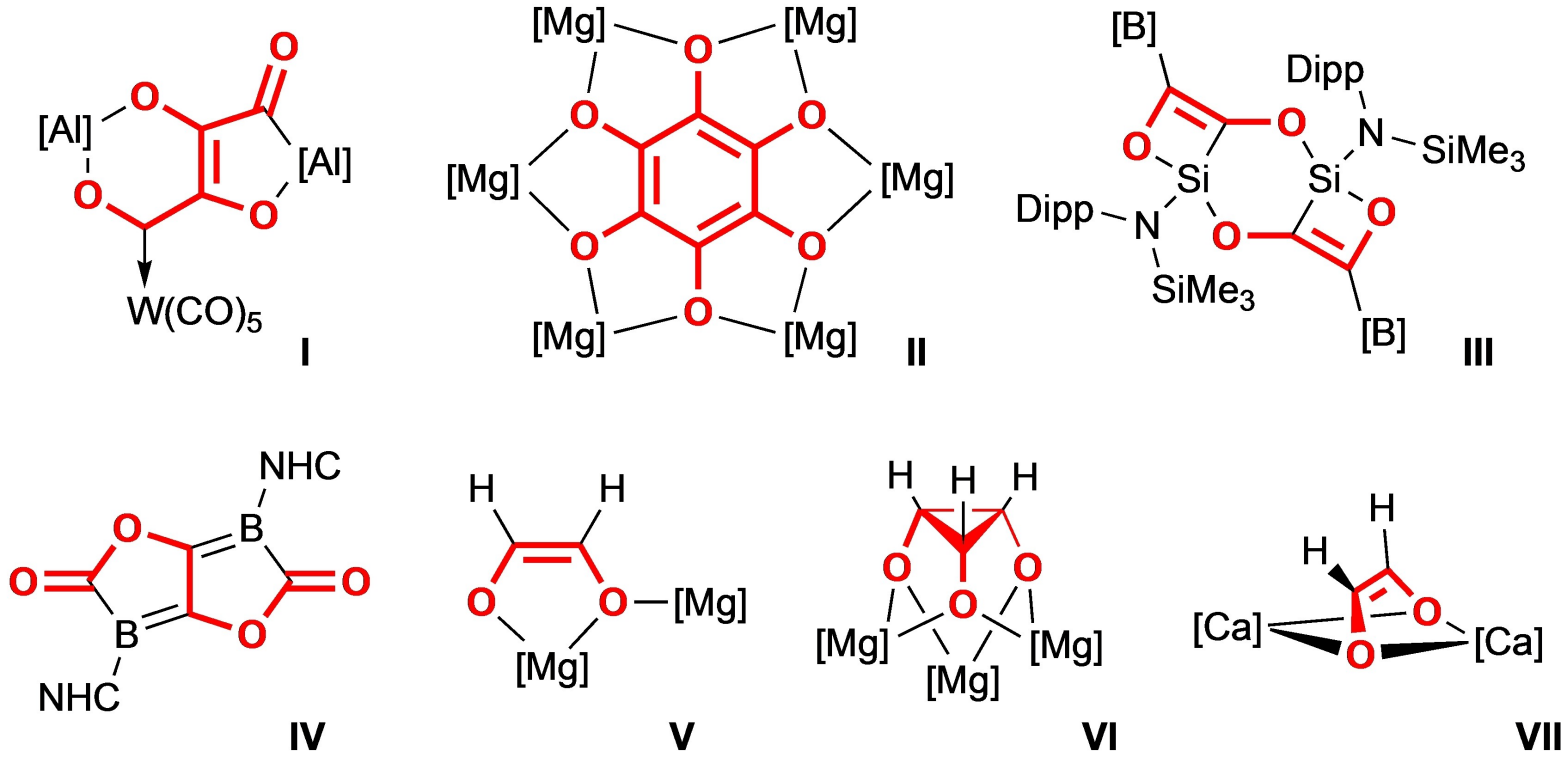
A selection of recently reported CO coupled products, isolated from reactions between main group compounds and CO. The atoms of the molecule originating from CO are shown in red.

Main group metal hydrides have also seen some limited success in reducing CO to form C−C bonds.^[13]^ These reactions have the added benefit of transferring hydrogen atoms onto the newly formed carbon‐oxygen chains, creating more complex and potentially useful CHO‐containing products. Notable examples here include two almost simultaneous reports by the groups of Jones and Hill, on a dimeric magnesium hydride activating two equivalents of CO to give a *cis*‐ethenediolate unit (**V**).[^14^, ^15^] Jones and co‐workers went on to show that decreasing the steric bulk of the *β*‐diketiminate ligand can instead lead to trimerisation of CO to yield a cyclopropanetriolate unit (**VI**).^[14]^ Hill and co‐workers later reported that a similar ethenediolate complex could also be synthesised using a dimeric *β*‐diketiminate stabilised calcium hydride complex (**VII**).^[16]^ Similar reactions have recently been reported by the groups of Okuda and Cheng, using a dimeric calcium and barium hydride complex, respectively.[^17^, ^18^]

Within all of the CO coupling studies however, very few have undergone/attempted the final step of realising the CO coupled fragment from the main group reagent to yield small organic molecules of synthetic use.^[19]^ This is likely due to these neutral compounds forming (multiple) strong bonds between the CO coupled motifs and the main group centre. Herein, we show that *anionic* magnesium and calcium hydride complexes can insert and couple two molecules of CO to give the alkaline earth metal coordinated *cis*‐ethenediolate compounds. Moreover, as these compounds are dianionic, the *cis*‐ethenediolate unit can easily be liberated in simple salt metathesis reactions to give small unsaturated disilyl ethers, versatile organic starting materials, with a high percentage of their mass originating from CO.

The *anionic* molecular group 2 hydrides reported in this work were initially prepared by an unconventional route. K_2_[(**NON**)MgH(THF)]_2_ (**1‐Mg, NON**=4,5‐bis(2,6‐diisopropylanilido)‐2,7‐di‐tertbutyl‐9,9‐dimethyl‐xanthene)) was synthesised by stirring a toluene solution of the neutral magnesium complex (**NON**)Mg(THF) over excess KC_8_ at 60 °C overnight (Scheme 1, *route a*). After workup, this gave **1‐Mg** in a reasonable crystalline yield (55 %). The calcium analogue K_2_[(**NON**)CaH(OEt_2_)]_2_ (**1‐Ca**) was synthesised via a similar route, by stirring a benzene solution of the previously reported (**NON**)Ca(OEt_2_)_2_ complex^[20]^ over excess KC_8_ at room temperature overnight (Scheme 1, *route a*). After workup, this gave the calcium analogue **1‐Ca** in good crystalline yields (74 %). Both **1‐Mg** and **1‐Ca** were also found to be accessible by heating benzene solutions of the same precursor complexes over excess KH (Scheme 1, *route b*). However, over multiple repeats, this reaction tended to be less reliable than *route a*, due to a further reaction occurring between the products (**1‐Mg** and **1‐Ca**) and KH, causing a decomposition of the products to K_2_(**NON**) and insoluble AeH_2_ (Ae=Mg or Ca).

**Scheme 1:**
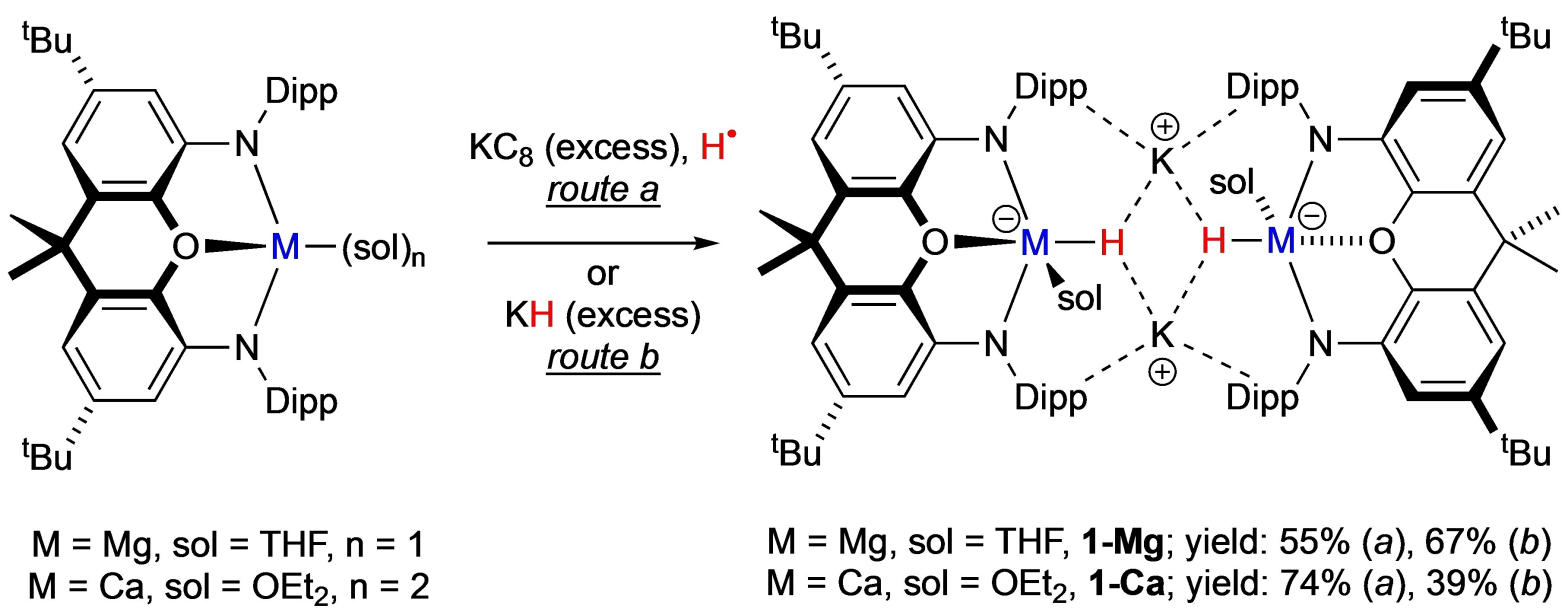
Synthesis of **1‐Mg** and **1‐Ca**.

Both **1‐Mg** and **1‐Ca** have been comprehensively characterised by various spectroscopic, diffraction and computational techniques. The ^1^H NMR spectra of **1‐Mg** and **1‐Ca** are highly informative. In both cases, a single **NON** ligand environment is seen, with the addition of resonances corresponding to one solvent molecule (THF in **1‐Mg** and Et_2_O in **1‐Ca**), which are considerably shifted from those reported for the “free” solvent in solution, indicating coordination. Moreover, an extra singlet with integration of 1H (per **NON** ligand) is observed in each spectrum (δ=2.72 ppm in **1‐Mg** and 3.63 ppm in **1‐Ca**) corresponding to the hydride. These hydride resonances are considerably more upfield compared to those in the previously reported neutral [(BDI)M(*μ*‐H)]_2_ systems (*cf* 4.03 ppm for M=Mg and 4.27 ppm for M=Ca).^[21]^

To investigate the solid state structures of **1‐Mg** and **1‐Ca** both compounds were additionally analysed by single crystal X‐ray and neutron diffraction (Figure 2 and Supporting Information).^[22]^ From the X‐ray diffraction data, the two complexes can be seen to be essentially isostructural, forming symmetric dimeric structures in the solid state. These dimeric structures are held together by various interactions between the anionic [(**NON**)MH(sol)]^−^ fragments and the potassium cations, with the two group 2 metal centres approximately 7 Å apart (Mg⋅⋅⋅Mg=6.923(1) Å, Ca⋅⋅⋅Ca=7.377(1) Å). This is a common motif for anionic complexes bearing the **NON** ligand with potassium cations, previously observed in systems such as the potassium aluminyl complex K_2_[Al(**NON**)]_2_.^[23]^ The group 2 metal centre in **1‐Mg** and **1‐Ca** are 5‐coordinate, with coordination by one tridentate **NON** ligand, one donor solvent and a hydride. The dimeric structure of **1‐Mg** and **1‐Ca** was also found to be retained in solution (C_6_D_6_) by diffusion‐ordered spectroscopy (DOSY) NMR experiments. Similar diffusion coefficients of 3.77(5)×10^−10^ m^2^ s^−1^ (**1‐Mg**) and 3.83(6)×10^−10^ m^2^ s^−1^ (**1‐Ca**) were recorded for the two complexes in C_6_D_6_, translating to hydrodynamic radii of approx. 9.6 Å for both complexes. This is almost identical to that reported for the dimeric potassium aluminyl complex K_2_[Al(**NON**)]_2_ (hydrodynamic radius of 9.7 Å)^[21]^ and considerably larger than the monomeric complex (**NON**)Mg(OEt_2_) (hydrodynamic radius of 7.6 Å)^[19]^ both bearing the same **NON** ligand. These results strongly suggest that the dimeric structure of **1‐Mg** and **1‐Ca** are retained in solution.

**Figure 2:**
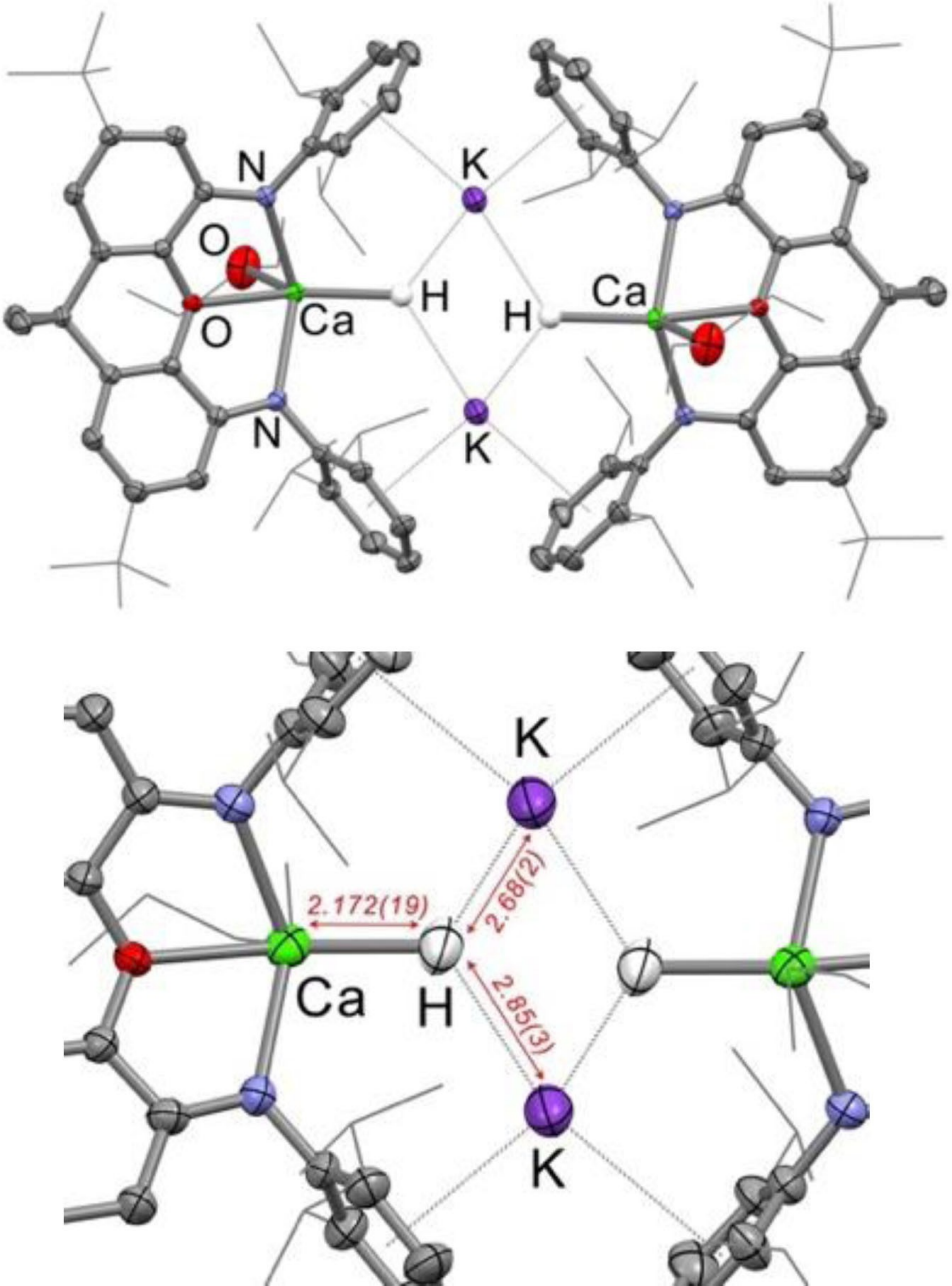
Molecular structure of **1‐Ca** (top) as determined by X‐ray crystallography and part of the molecular structure of **1‐Ca** as determined by neutron diffraction (bottom), with key distances displayed in red (Å).

As X‐ray diffraction is inherently unreliable at accurately determining the position of hydrogen atoms, **1‐Mg** and **1‐Ca** were additionally characterised by neutron diffraction (Figure 2 and Supporting Information).^[22]^ Single crystal neutron diffraction of **1‐Mg** was successful in locating the hydrides in the K_2_Mg_2_ pocket, with a Mg−H bond length of 1.71(7) Å and H⋅⋅⋅K interactions of 2.67(11) and 2.92(12) Å. The hydride was also found to be essentially *trans* to the oxygen in the xanthene backbone of the ligand, with an O−Mg−H angle of 169(3)°. A similar structure was found for **1‐Ca**, with a Ca−H bond length of 2.172(19) Å, H⋅⋅⋅K interactions of 2.68(2) and 2.85(3) Å and a xanthene backbone O−Ca−H angle of 160.8(7)° (Figure 2). The structures of these mixed group 1/2 hydrides are best compared to the mixed alkali metal/magnesium hydride complexes recently reported by Mulvey and co‐workers.^[24]^ However, in the reported complexes, the hydrides bridge the two group 2 centres. In the case of **1‐Mg** and **1‐Ca** this is not the case—the hydrides here are best described as “terminal”, but stabilised by interactions with two potassium cations. A terminal calcium hydride is yet to be reported; we do not claim **1‐Ca** to be the first truly terminal calcium hydride, but believe it to be the closest example reported to date.

The electronic structures of **1‐Mg** and **1‐Ca** were additionally probed using density functional theory (DFT) calculations. The optimised gas phase structures and calculated bond parameters for **1‐Mg** and **1‐Ca** are in good agreement with those experimentally observed. The M−H bond lengths were found to be 1.839 (**1‐Mg**) and 2.170 Å (**1‐Ca**). Inspection of the calculated frontier molecular orbitals reveals that the M−H σ‐interaction is the HOMO‐10 for **1‐Mg** and the HOMO‐7 for **1‐Ca**. Further bonding analyses confirm that the M−H interaction can be described as ionic in nature: the calculated Wiberg bond indices (WBIs) are 0.1980 (**1‐Mg**) and 0.1491 (**1‐Ca**) (see Supporting Information for further details).

The synthesis of **1‐Mg** and **1‐Ca** by *route a* was initially unexpected, as no typical hydride source was added to either reaction (Scheme 1). Furthermore, the reactions are reminiscent to work recently reported by Hill, McMullen and co‐workers, where a similar bulky diamido magnesium complex was reduced with sodium metal,^[25]^ which led to the formal one‐electron reduction of the metal centre, to give a dianionic, dimagnesium(I) complex with a Mg−Mg bond. In our case, we postulate a similar one‐electron reduction of the (**NON**)M starting materials is also occurring, but due to the different steric and electronic demands of the ligand sets, the **NON** complexes do not dimerise to give metal‐metal bonded species, but rather extract H⋅ from somewhere in the reaction medium.^[26]^ To investigate whether this was a tenable mechanism in the synthesis of **1‐Mg** and **1‐Ca**, the reactions were repeated but in deuterated solvents (d_8_‐toluene for **1‐Mg** and d_6_‐benzene for **1‐Ca**). Analysis of the resulting products by ^1^H NMR spectroscopy revealed a significant decrease in the integral of the hydride resonance for both compounds (85 % decrease for **1‐Mg** and 90 % for **1‐Ca**), with the remainder of the spectra essentially unchanged. This indicates that between 85–90 % of the hydride in these products was deuteride, further evidenced by strong resonances appearing in ^2^H NMR spectra at δ=2.76 and 3.70 ppm for **1‐Mg** and **1‐Ca**, respectively. This suggests that approx. 85–90 % of the hydride atoms in **1‐Mg** and **1‐Ca** are being abstracted from the reaction solvent, with the remaining 10–15 % coming from other sources (**NON** ligand, coordinated solvent etc.). This mixture of sources for the hydride is consistent with a radical extraction mechanism.

Having these complexes in hand, the reactivity of **1‐Mg** and **1‐Ca** towards CO was investigated. A benzene solution of **1‐Mg** was exposed to CO (1 atm) at room temperature. Monitoring this reaction by ^1^H NMR, it was found that over the course of 2 days, the reaction proceeds to completion to give a single new product with a characteristic resonance at δ=5.23 ppm. A similar reaction was observed between **1‐Ca** and CO (1 atm), although this reaction proceeded much quicker (<2 mins) to yield a single new product with a characteristic resonance at δ=5.23 ppm. Both products were crystallographically characterised and their solid state structures determined (Figure 3).^[22]^

**Figure 3:**
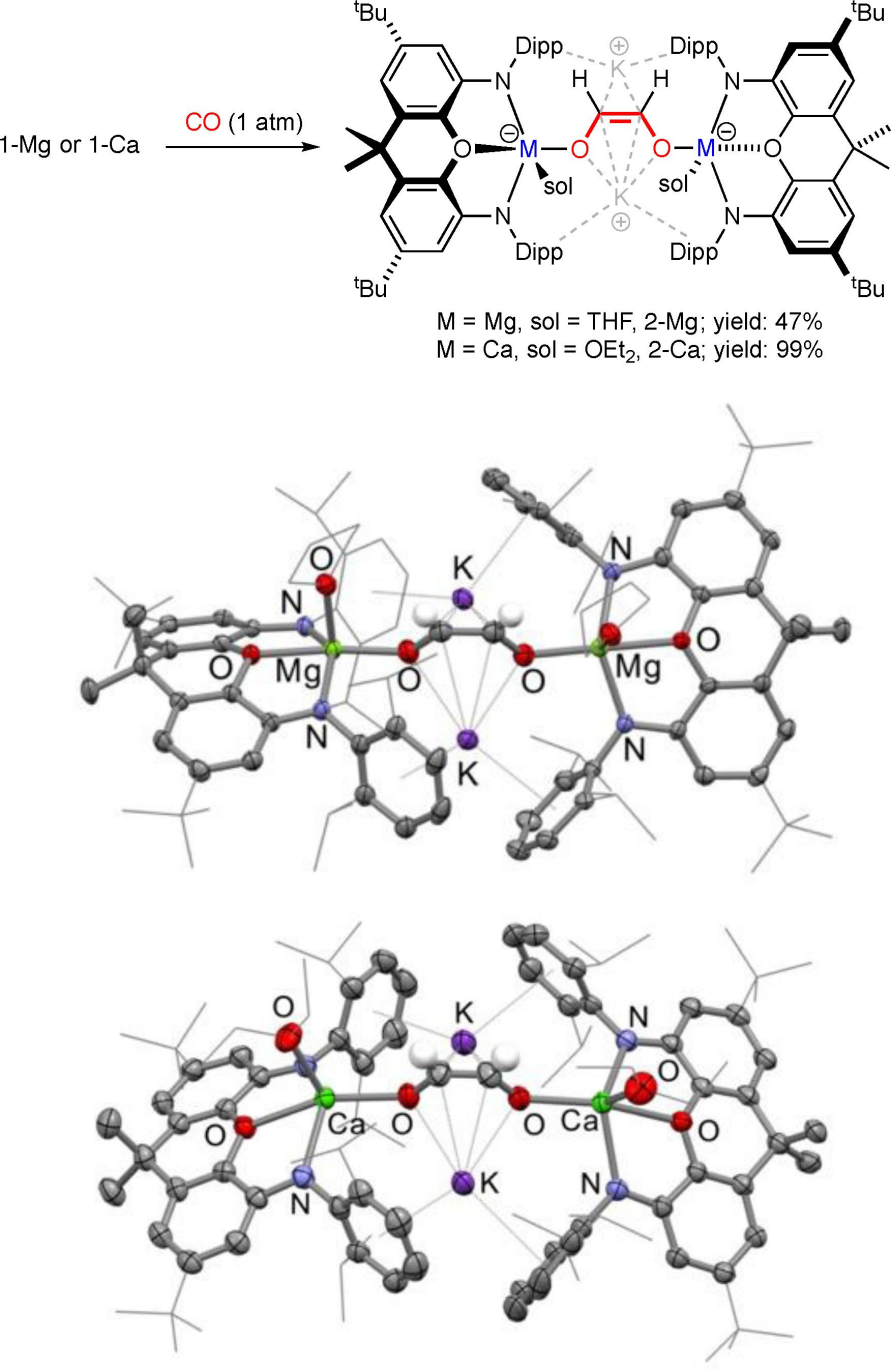
Synthesis of **2‐Mg** and **2‐Ca** (top) and molecular structures of **2‐Mg** (centre) and **2‐Ca** (bottom) as determined by X‐ray crystallography.

From X‐ray crystallography, it was revealed that both **1‐Mg** and **1‐Ca** were found to C=C couple two molecules of CO to give the essentially isostructural dianionic *cis*‐ethenediolate complexes K_2_[{(**NON**)M(sol)}_2_(*μ*‐O_2_C_2_H_2_)] (M=Mg, sol=THF, **2‐Mg**; M=Ca, sol=Et_2_O, **2‐Ca**). Both structures feature a bridging ethenediolate unit, with each group 2 center only coordinating/interacting with one of the two oxygen atoms. This contrasts with the neutral ethenediolate complexes reported by Jones and Hill (**V** and **VII** Figure 1),[^14^, ^15^, ^16^] which show higher coordination numbers to the ethenediolate unit. The potassium cations in the solid state structures of **2‐Mg** and **2‐Ca** lie on either side of the ethenediolate unit, and each form *η* ^4^ interactions with the [O_2_C_2_H_2_]^2−^ unit in an inverse‐sandwich type of structure. Worthy of note is the fact that the *cis*‐isomer of ethenediolate is formed exclusively, with no evidence of the *trans*‐isomer ever being observed by X‐ray or NMR. This is likely due to the dimeric nature of the hydrides templating the formation of the *cis*‐ethenediolate unit, similar to that previously proposed for the dimeric *β*‐diketiminate systems.[^14^, ^15^, ^16^]

As **2‐Mg** and **2‐Ca** are dianionic ethenediolate complexes, they can be viewed as simple Lewis acid (Mg, Ca) stabilised complexes of the K_2_[O_2_C_2_H_2_] salt. It was therefore hypothesised that these complexes could act as nucleophilic sources of the *cis*‐ethenediolate dianion towards electrophiles in simple salt metathesis reactions. With this in mind, 2 equivalents of silyl chloride R_3_SiCl (R=Me or ^i^Pr) were added to THF solutions of **2‐Mg** and **2‐Ca** at room temperature. These reactions all led to good yields of the unsaturated disilyl ethers [R_3_SiOC(H)]_2_ (R=Me, **3 a**; R=^i^Pr, **3 b**) (Scheme 2). These reactions were accompanied by the formation of 2 equiv of both KCl and the corresponding group 2 complex (**NON**)Mg(THF) or (**NON**)Ca(THF)_2_.

**Scheme 2:**
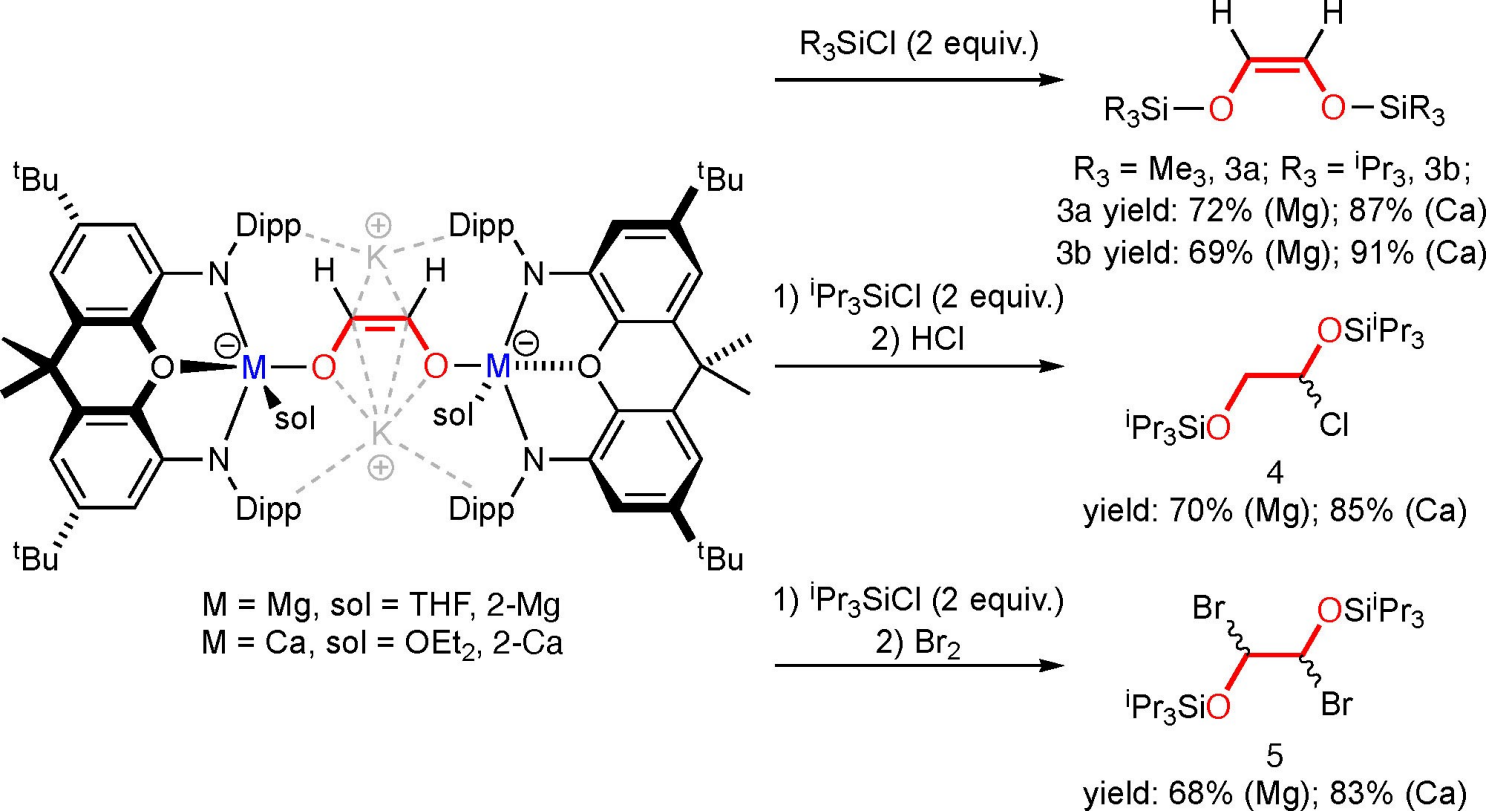
Synthesis of a series of small organic molecules from **2‐Mg** and **2‐Ca**. Parts of the molecule originating from CO are shown in red. Yields for **4** and **5** reported over 2 steps.

These small unsaturated disilyl ethers are versatile starting materials in a range of organic reactions.^[27]^ For example, **3 b** can easily be transformed into silyl protected ethanediols by simple addition reactions with HCl (**4**) or Br_2_ (**5**), yielding a series of useful, functionalised small organic molecules in which a high percentage of their mass originated from CO (Scheme 2).

Generation of the CO‐coupled small molecules **3**–**5** is not yet catalytic, mainly due to the incompatibility of reagents required for each synthetic step (KC_8_ and R_3_SiCl for example). That said, the liberation of the [C_2_H_2_O_2_]^2−^ unit from **2‐Mg** or **2‐Ca** with silyl chlorides does return the neutral (**NON**)Mg(THF) or (**NON**)Ca(THF)_2_ complex, which can be recycled resulting in a closed synthetic cycle (Scheme 3). (**NON**)Mg(THF) is the exact starting material used to synthesise hydride **1‐Mg**, but (**NON**)Ca(THF)_2_ on the other hand requires a simple exchange of the coordinated THF molecules with Et_2_O before the reagent can be reused. This is readily achieved by heating the complex under high vacuum before dissolving the residue in Et_2_O to give (**NON**)Ca(OEt_2_)_2_.

**Scheme 3:**
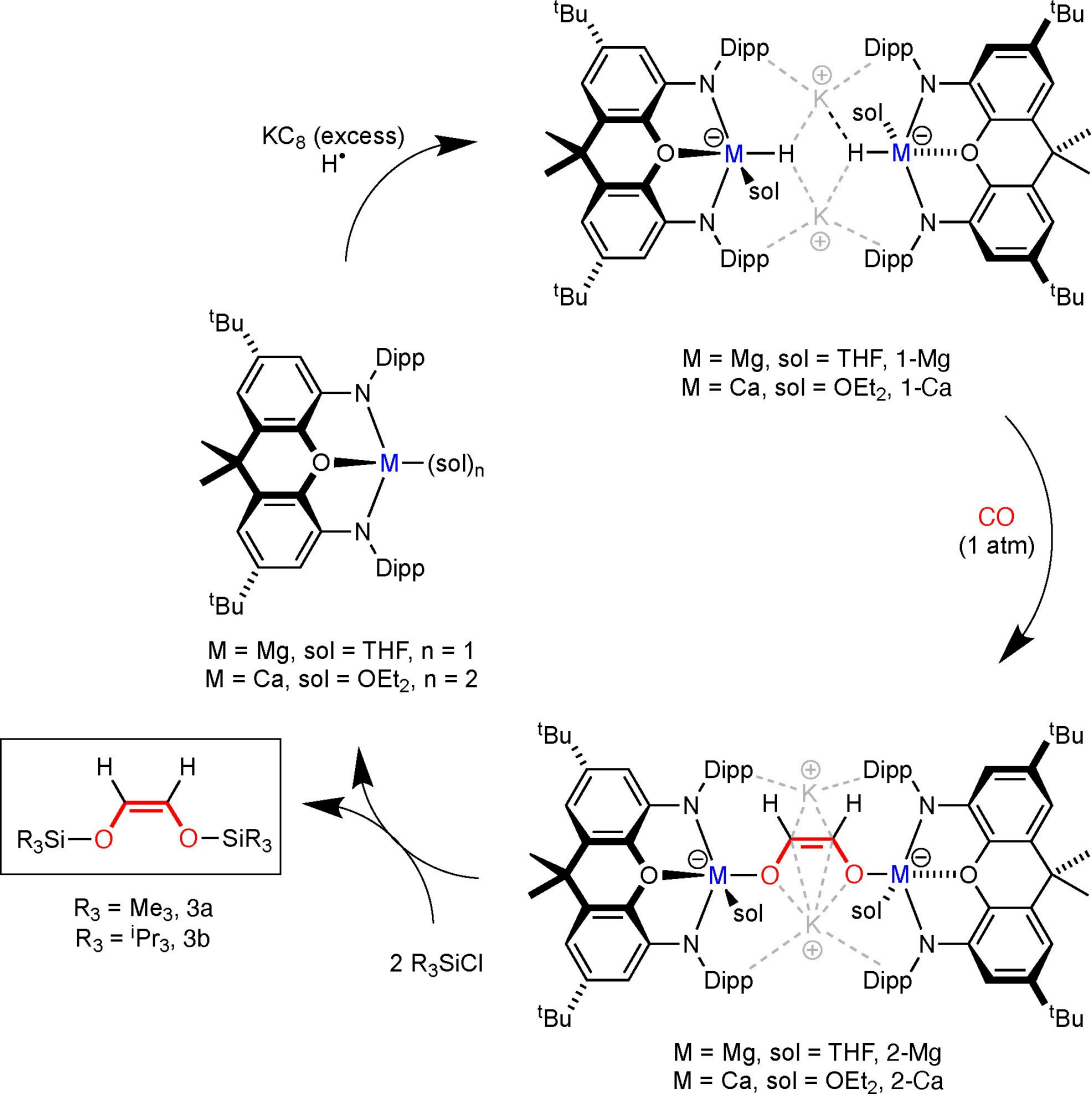
Closed synthetic cycle showing the conversion of CO into unsaturated disilyl ethers **3 a** and **3 b**.

## Conflict of interest

The authors declare no conflict of interest.

## Supporting information

As a service to our authors and readers, this journal provides supporting information supplied by the authors. Such materials are peer reviewed and may be re‐organized for online delivery, but are not copy‐edited or typeset. Technical support issues arising from supporting information (other than missing files) should be addressed to the authors.

Supporting Information

Supporting Information

## Data Availability

The data that support the findings of this study are available in the supplementary material of this article.
