# Supplementary material for: Anionic Magnesium and Calcium Hydrides: Transforming CO into Unsaturated Disilyl Ethers
Source: Angew Chem Int Ed Engl. 2022 Nov 29;62(1):e202215218. doi: 10.1002/anie.202215218 (PMC10100151; doi:10.1002/anie.202215218)
Supplement: Supplementary file 1 — Supporting Information [file ANIE-62-0-s002.pdf]

Angewandte Chemie
Eine Zeitschrift der Gesellschaft Deutscher Chemiker

Supporting Information

Anionic Magnesium and Calcium Hydrides: Transforming CO into Unsaturated Disilyl Ethers

*J. S. McMullen, R. Huo, P. Vasko, A. J. Edwards, J. Hicks**

Supplementary Information

This PDF files includes:

Materials and Methods

Synthetic and characterising data for new compounds	S2
^{1}H and ^{13}C NMR spectra of new compounds	S8
Diffusion ordered NMR spectroscopy (DOSY) experimental details	S26
X-ray crystallographic studies	S28
Neutron Laue diffraction experiment details	S22
Computational details	S36
References	S56

Figs. S1 – S31

Tables S1 – S5

(57 pages total)

Materials and Methods

Synthetic and characterising data for new compounds

General considerations. All manipulations were carried out using standard Schlenk line or dry-box techniques under an atmosphere of argon. Solvents were degassed by sparging with argon and dried by passing through a column of the appropriate drying agent. NMR spectra were measured in benzene-d_6, which was dried over potassium, distilled under reduced pressure and stored under argon in a Teflon valve ampoule. NMR samples were prepared under argon in 5 mm Norell Select Series NMR Tube fitted with J. Young Teflon valves. ^{1}H and ^{13}C{^{1}H} NMR spectra were recorded on a Bruker Avance 400 or 700 MHz spectrometer at 298 K and referenced internally to residual protio-solvent (^{1}H) or solvent (^{13}C) resonances and are reported relative to tetramethylsilane (δ = 0 ppm). Assignments were confirmed using two-dimensional ^{1}H-^{1}H and ^{13}C-^{1}H NMR correlation experiments. Chemical shifts are quoted in δ (ppm) and coupling constants in Hz. Elemental analyses were carried out by Dr Remi Rouquette at the Macquarie Analytical & Workshop Facility (MAWF), Macquarie University or by Elemental Microanalysis Ltd., Devon. H_2(**NON**), (**NON**)Mg(OEt_2) and (**NON**)Ca(OEt_2)$_2$ were prepared by literature methods.[S1,S2] All other reagents were used as received.

Synthesis of (NON)Mg(THF): (**NON**)Mg(OEt_2) (2.00 g, 2.60 mmol) was dissolved in tetrahydrofuran (30 mL) and stirred at room temperature for 5 minutes. The solvent was removed under reduced pressure and the resulting solid was heated to 150 °C under reduced pressure for 2 hours to yield (**NON**)Mg(OEt_2) as a colourless powder. **Yield:** 1.86 g (2.42 mmol, 93%). **^{1}H NMR** (400 MHz, C_6D_6): 0.71 (br, 4H, THF- OCH_2CH_2), 1.09 (d, $^3J_{HH}$ = 7.0 Hz, 12H, Dipp-^{i}Pr-CH_3), 1.26 (d, $^3J_{HH}$ = 6.8 Hz, 12H, Dipp-^{i}Pr-CH_3), 1.35 (s, 18H, ^{t}Bu-CH_3), 1.72 (s, 6H, CMe$_2$-CH_3), 2.94 (br, 4H, THF-OCH_2) 3.55 (hept, $^3J_{HH}$ = 6.4 Hz, 4H, Dipp-^{i}Pr-C*H*), 6.33 (s, 2H, Xanth-C$_{ortho}$*H*), 6.72 (s, 2H, Xanth-C$_{para}$*H*), 7.14-7.24 (m, 6H, Dipp-C_6H_3-C*H*); **^{13}C{^{1}H} NMR** (101 MHz, C_6D_6): 24.5 (Dipp-^{i}Pr-CH_3), 24.6 (THF-OCH_2CH_2), 25.5 (Dipp-^{i}Pr-*C*H$_3$), 28.5 (Dipp-^{i}Pr-*C*H), 30.2 (CMe$_2$-*C*H$_3$), 32.1 (^{t}Bu-*C*H$_3$), 35.2 (^{t}Bu-*C*Me$_3$), 36.0 (*C*Me$_2$), 71.1 (THF-O*C*H$_2$) 104.7, 108.9, 124.0, 124.1, 130.4, 137.9, 146.1, 147.1, 147.3, 147.6 (Ar-*C*); **IR** *υ*/cm^{-1} (Nujol): 1617(m), 1575(m), 1411(m), 1335(s), 1306(s), 1252(s), 1201(s), 1112(m), 1014(m), 935(m), 895(m), 859(m), 773(s), 662(m), 527(m); **Elemental Analysis** - calc. for $C_{51}H_{70}MgN_2O_2$: C 79.82%, H 9.19%, N 3.65%; found: C 79.82% H 9.34%, N 3.87%

Preparation of K_2[(NON)MgH(THF)]$_2$ (1-Mg), *route a*: To a suspension of KC_8 (1.10 g, 8.11 mmol) in toluene (10 mL) was added a solution of (**NON**)Mg(THF) (1.23 g, 1.60 mmol) in toluene (10 mL) at room temperature. The reaction mixture heated to 60 °C and stirred overnight, whereupon the KC_8 changed colour from bronze to black. The reaction mixture was allowed to cooled, filtered and volatiles from the filtrate *in vacuo* to give a pale yellow powder. This pale yellow powder was dissolved in a minimal volume of warm benzene (ca. 4

mL at 60 °C) and slowly cooled to room temperature overnight to give **1-Mg** as large colourless crystals. **Yield:** 0.710 g (0.440 mmol, 55%).

Preparation of K_2[(NON)MgH(THF)]$_2$ (1-Mg), *route b*: Into a 50 mL J. Young reaction flask containing a mixture of (**NON**)Mg(THF) (0.500 g, 0.652 mmol) and KH (0.250 g, 6.25 mmol) was added benzene (10 mL) at room temperature. The reaction vessel was sealed and heated to 100 °C where it was stirred rapidly for 15 minutes, before stopping the stirring and allowing to cool to room temperature. The reaction mixture was filtered, and volatiles removed from the filtrate concentrated *in vacuo* to give a mixture of 85:15 **1-Mg** and K_2(**NON**) as an off white powder. This powder redissolved in the minimum volume of warm benzene (ca. 3 mL) to give pure **1-Mg** as colourless crystals. **Yield** 0.352 g (0.218 mmol, 67%). N.B. The rate and yield of this reaction is strongly influenced by the potassium hydride used. Grinding the potassium hydride with a mortar and pestle immediately before use significantly increases the rate of reaction. The reaction time is also important as longer reaction times lead to decomposition of **1-Mg** and the formation of K_2(**NON**). **^{1}H NMR** (400 MHz, C_6D_6): 0.95 (d, $^3J_{HH}$ = 6.7 Hz, 24H, Dipp-^{i}Pr-CH_3), 1.23 (d, $^3J_{HH}$ = 6.5 Hz, 24H, Dipp-^{i}Pr-CH_3), 1.36 (s, 36H, ^{t}Bu-CH_3), 1.44 (br, 8H, THF-OCH$_2$CH_2), 1.73 (s, 12H, CMe$_2$-CH_3), 2.72 (s, 2H, MgH), 3.60 (hept, $^3J_{HH}$ = 6.7 Hz, 8H, Dipp-^{i}Pr-CH), 3.81 (br, 8H, THF-OCH_2), 6.01 (s, 4H, Xanth-C$_{ortho}H$), 6.62 (s, 4H, Xanth-C$_{para}H$), 7.05-7.13 (m, 12H, Dipp-C$_6H_3$); **^{13}C{^{1}H} NMR** (101 MHz, C_6D_6): 25.2, 25.4 (Dipp-^{i}Pr-CH$_3$), 25.6 (THF-OCH$_2$$CH_2$), 27.6 (Dipp-^{i}Pr-$C$H), 31.0 (CMe$_2$-$CH_3$), 32.2 (^{t}Bu-$CH_3$) 35.1 ($CMe_3$), 35.6 ($CMe_2$) 69.5 (THF-O$CH_2$), 103.0, 106.8, 123.5, 123.7, 146.5, 147.4, 147.8, 151.6 (Ar-C); **IR** υ/cm^{-1} (Nujol): 1612(m), 1573(m), 1486(s), 1428(s), 1411(m), 1339(s), 1306(s), 1249(s), 1202(s), 1114(m), 1017(m), 879(m), 775(m), 727(s), 678(s), 522(s), 464(m), 425(m); **Elemental Analysis** - calc. for $C_{51}H_{71}KMgN_2O_2$: C 75.85%, H 8.86%, N 3.47% found: C 75.85%, H 9.48%, N 3.43%.

Preparation of K_2[(NON)CaH(OEt$_2$)]$_2$ (1-Ca), *route a*: To a suspension of KC_8 (0.250 g, 1.85 mmol) in benzene (10 mL) was added a solution of (**NON**)Ca(OEt$_2$)$_2$ (0.500 g, 0.582 mmol) in benzene (10 mL) at room temperature. The reaction mixture was stirred overnight at room temperature, producing a pale yellow solution and a black precipitate. The reaction mixture was filtered and the filtrate concentrated *in vacuo* (ca. 4 mL), which was left to stand overnight at room temperature to give **1-Ca** as large colourless crystals. **Yield** 0.355 g (0.215 mmol, 74%).

Preparation of K_2[(NON)CaH(OEt$_2$)]$_2$ (1-Ca), *route b*: Into a 25 mL J. Young reaction flask containing a mixture of (**NON**)Ca(OEt$_2$)$_2$ (0.200 g, 0.232 mmol) and KH (0.050 g, 1.25 mmol) was added benzene (3 mL) at room temperature. The reaction vessel was sealed and heated to 100 °C where it was stirred rapidly for 1 hour, before stopping the stirring and allowing to cool to room temperature. The reaction mixture was filtered, and volatiles removed from the filtrate concentrated *in vacuo* to give a mixture of 90:10 **1-Ca** and K_2(**NON**) as an off white powder. This powder redissolved in the minimum volume of warm benzene (ca 1 mL) to give pure **1-Ca** as colourless crystals. **Yield** 0.075 g (0.045 mmol, 39%). N.B. The rate and yield of this reaction is

strongly influenced by the potassium hydride used. Grinding the potassium hydride with a mortar and pestle immediately before use significantly increases the rate of reaction. The reaction time is also important as longer reaction times lead to decomposition of **1-Ca** and the formation of K_2(**NON**). **^{1}H NMR** (400 MHz, C_6D_6, 298 K): δ = 0.95 (d, $^3J_{HH}$ = 7.1 Hz, 24H, CH(CH_3)$_2$), 0.99 (br., 12H, OCH$_2$CH_2), 1.30 (d, $^3J_{HH}$ = 6.6 Hz, 24H, CH(CH_3)$_2$), 1.34 (s, 36H, C(CH_3)$_3$), 1.77 (s, 12H, C(CH_3)$_2$), 3.35 (q, $^3J_{HH}$ = 7.1 Hz, 8H, OCH_2CH$_3$), 3.55 (sept., $^3J_{HH}$ = 6.8 Hz, 8H, CH(CH$_3$)$_2$), 3.63 (s, 2H, CaH), 6.09 (d, $^4J_{HH}$ = 2.2 Hz, 4H, NON-*o*-CH), 6.64 (d, $^4J_{HH}$ = 2.2 Hz, 4H, NON-*p*-CH), 7.06 (t, $^3J_{HH}$ = 7.5 Hz, 4H, Dipp-*p*-CH), 7.16 (d, $^3J_{HH}$ = 7.5 Hz, 8H, Dipp-*m*-CH); ^{13}C{^{1}H} NMR (101 MHz, C_6D_6): δ = 15.0 (OCH$_2$$CH_3$), 25.0 (br. - CH($CH_3$)$_2$), 28.0 ($C$H(CH$_3$)$_2$), 32.1 (XA-C($CH_3$)$_2$), 32.2 (C($CH_3$)$_3$), 35.0 ($C$(CH$_3$)$_3$), 35.4 (XA-$C$(CH$_3$)$_2$), 66.6 (O$CH_2CH_3$), 103.6, 107.2, 122.0, 123.5, 129.6, 139.3, 146.2, 146.4, 148.4, 151.5 (Ar-C); **IR** υ/cm^{-1} (Nujol): 2951(s), 2862(m) 1605(m), 1575(m), 1480(s), 1464(s), 1426(s), 1413(m), 1355(m), 1342(m), 1306(s), 1248(s), 1200(s), 1187(s), 1116(m), 1054(m), 1015(m), 1007(m), 874(m), 843(m), 796(m), 774(m), 548(m), 457(s), 421(s); **Elemental Analysis** - calc. for $C_{102}H_{146}Ca_2N_4O_4$: C 74.22%, H 8.92%, N 3.39%, found: C 74.25%, H 9.25%, N 3.66%.

Preparation of K_2[(NON)CaH(OEt$_2$)]$_2$ (1-Ca), route a: To a suspension of KC_8 (0.250 g, 1.85 mmol) in benzene (10 mL) was added a solution of (**NON**)Ca(OEt$_2$)$_2$ (0.500 g, 0.582 mmol) in benzene (10 mL) at room temperature. The reaction mixture was stirred overnight at room temperature, producing a pale yellow solution and a black precipitate. The reaction mixture was filtered and the filtrate concentrated *in vacuo* (ca. 4 mL), which was left to stand overnight at room temperature to give **1-Ca** as large colourless crystals. **Yield** 0.355 g (0.215 mmol, 74%). **^{1}H NMR** (400 MHz, C_6D_6, 298 K): δ = 0.95 (d, $^3J_{HH}$ = 7.1 Hz, 24H, CH(CH_3)$_2$), 0.99 (br., 12H, OCH$_2$CH_2), 1.30 (d, $^3J_{HH}$ = 6.6 Hz, 24H, CH(CH_3)$_2$), 1.34 (s, 36H, C(CH_3)$_3$), 1.77 (s, 12H, C(CH_3)$_2$), 3.35 (q, $^3J_{HH}$ = 7.1 Hz, 8H, OCH_2CH$_3$), 3.55 (sept., $^3J_{HH}$ = 6.8 Hz, 8H, CH(CH$_3$)$_2$), 3.63 (s, 2H, CaH), 6.09 (d, $^4J_{HH}$ = 2.2 Hz, 4H, NON-*o*-CH), 6.64 (d, $^4J_{HH}$ = 2.2 Hz, 4H, NON-*p*-CH), 7.06 (t, $^3J_{HH}$ = 7.5 Hz, 4H, Dipp-*p*-CH), 7.16 (d, $^3J_{HH}$ = 7.5 Hz, 8H, Dipp-*m*-CH); ^{13}C{^{1}H} NMR (101 MHz, C_6D_6): δ = 15.0 (OCH$_2$$CH_3$), 25.0 (br. - CH($CH_3$)$_2$), 28.0 ($C$H(CH$_3$)$_2$), 32.1 (XA-C($CH_3$)$_2$), 32.2 (C($CH_3$)$_3$), 35.0 ($C$(CH$_3$)$_3$), 35.4 (XA-$C$(CH$_3$)$_2$), 66.6 (O$CH_2CH_3$), 103.6, 107.2, 122.0, 123.5, 129.6, 139.3, 146.2, 146.4, 148.4, 151.5 (Ar-C); **IR** υ/cm^{-1} (Nujol): 2951(s), 2862(m) 1605(m), 1575(m), 1480(s), 1464(s), 1426(s), 1413(m), 1355(m), 1342(m), 1306(s), 1248(s), 1200(s), 1187(s), 1116(m), 1054(m), 1015(m), 1007(m), 874(m), 843(m), 796(m), 774(m), 548(m), 457(s), 421(s); **Elemental Analysis** - calc. for $C_{102}H_{146}Ca_2N_4O_4$: C 74.22%, H 8.92%, N 3.39%, found: C 74.25%, H 9.25%, N 3.66%.

Preparation of K_2[{(NON)Mg(THF)}$_2$O$_2$C$_2$H$_2$] (2-Mg): A solution of compound **1-Mg** (1.00 g, 0.619 mmol) in benzene (5 mL) was prepared in a J. Youngs reaction flask (50 mL). The headspace of the reaction flask was purged with carbon monoxide (~1 atm) for 30 seconds, before sealing, briefly agitating and leaving to stand at room temperature for 48 hours, which gave **2-Mg** as colourless crystals. **Yield:** 0.483 g (0.289 mmol, 47%). **^{1}H NMR** (400 MHz, C_6D_6): 0.97 (d, $^3J_{HH}$ = 6.8 Hz, 24H, Dipp-^{i}Pr-CH_3), 1.21 (d, $^3J_{HH}$ = 6.7 Hz, 24H, Dipp-^{i}Pr-CH_3),

1.35 (br, 44H, ^{t}Bu-CH_3 & THF-OCH$_2$CH_2), 1.74 (s, 12H, CMe$_2$-CH_3), 3.57 (hept, $^{3}J_{HH}$ = 7.6 Hz, 8H, Dipp-^{i}Pr-CH), 3.86 (br, 8H, THF-OCH_2), 5.23 (s, 2H, Mg-OCH), 5.97 (d, $^{4}J_{HH}$ = 1.5 Hz, 4H, Xanth-C$_{ortho}$$H$), 6.64 (d, $^{4}J_{HH}$ = 1.5 Hz, 4H, Xanth-C$_{para}$$H$), 7.05-7.13 (m, 12H, Dipp-C$_6$$H_3$); **^{13}C{^{1}H} NMR** (101 MHz, C$_6$D$_6$): 25.0 (THF-OCH$_2$CH$_2$), 25.1 (Dipp-^{i}Pr-CH$_3$), 27.7 (Dipp-^{i}Pr-CH), 32.2 (CMe$_2$-CH$_3$ & ^{t}Bu-CH$_3$), 35.1 (CMe$_3$), 35.6 (CMe$_2$), 70.2 (THF-OCH$_2$), 103.6, 107.3, 124.0, 124.2, (Ar-C), 128.6 (Mg-OCH), 129.1 137.6, 146.5, 147.3, 147.6, 151.4 (Ar-C); **IR** υ/cm^{-1} (cm^{-1}) (Nujol): 1619(m), 1600(m), 1576(m), 1485(s), 1427(s), 1408(s), 1339(s), 1303(s), 1250(s), 1212(s), 1203(s), 1119(s), 1018(s), 936(m), 891(m), 848(s), 774(s), 675(m), 661(m), 526(m), 427(s). **Elemental Analysis** – calc. for $C_{104}H_{142}K_2Mg_2N_4O_6$: C 74.75%, H 8.57%, N 3.35%; found: C 74.70%; H 9.10%, N 3.60%.

Preparation of K$_2$[{(NON)Ca(OEt$_2$)}$_2$O$_2$C$_2$H$_2$] (2-Ca): A solution of K$_2$[(**NON**)CaH(OEt$_2$)]$_2$ (0.200 g, 0.121 mmol) in toluene (10 mL) was prepared in J. Young sample flask (50 mL). The solution was frozen, the atmosphere evacuated (5.0 x 10^{-3} mbar) and refilled with dry CO (1.0 bar). The flask was slowly warmed to room temperature and stirred overnight, whereupon volatiles were removed *in vacuo* to give **2-Ca** as a colourless solid. **Yield** 0.205 g (0.120 mmol, 99%). N.B. X-ray quality crystals were obtained by recrystallizing this solid from warm benzene. **^{1}H NMR** (400 MHz, C$_6$D$_6$, 298 K): δ = 0.87 (t, $^{3}J_{HH}$ = 7.0 Hz, 12H, OCH$_2$CH_2), 0.97 (d, $^{3}J_{HH}$ = 6.9 Hz, 24H, CH(CH_3)$_2$), 1.28 (d, $^{3}J_{HH}$ = 6.8 Hz, 24H, CH(CH_3)$_2$), 1.33 (s, 36H, C(CH_3)$_3$), 1.80 (s, 12H, C(CH_3)$_2$), 3.43 (q, $^{3}J_{HH}$ = 7.1 Hz, 8H, OCH_2CH$_3$), 3.51 (sept., $^{3}J_{HH}$ = 6.8 Hz, 8H, CH(CH$_3$)$_2$), 5.23 (s, 2H, OCH), 6.07 (d, $^{4}J_{HH}$ = 2.1 Hz, 4H,NON-*o*-CH), 6.64 (d, $^{4}J_{HH}$ = 2.1 Hz, 4H, NON-*p*-CH), 7.09 (t, $^{3}J_{HH}$ = 7.5 Hz, 4H, Dipp-*p*-CH), 7.18 (d, $^{3}J_{HH}$ = 7.5 Hz, 8H, Dipp-*m*-CH); **^{13}C{^{1}H} NMR** (101 MHz, C$_6$D$_6$): δ = 14.3 (OCH$_2$CH$_3$), 24.6, 25.1 (br. - CH(CH$_3$)$_2$), 28.1 (CH(CH$_3$)$_2$), 32.1 (C(CH$_3$)$_3$), 35.2 (XA-C(CH$_3$)$_2$), 34.9 (C(CH$_3$)$_3$), 35.2 (XA-C(CH$_3$)$_2$), 64.9 (OCH$_2$CH$_3$), 104.1, 107.2, 122.0, 123.7, 128.6, 129.0, 138.7, 145.6, 146.3, 148.5, 152.5; **IR** υ/cm^{-1} (Nujol): 2955(s), 2924(s), 2864(s), 1611(m), 1576(s), 1463(s), 1426(s), 1381(m), 1342(s), 1302(s), 1250(s), 1202(s), 1116(s), 1052(m), 1018(m), 938(m), 874(m), 836(s), 794(m), 769(m), 659(m), 550(m), 511(m), 422(m); **Elemental Analysis** – unfortunately, even after numerous attempts, a reliable elemental analysis could not be acquired for this compound.

General preparation of 3a/3b from 2-Mg. To a solution of **2-Mg** (0.200 g, 0.120 mmol) in THF (5 mL) was added the appropriate chlorosilane (0.263 mmol, 2.2 equiv.) at room temperature. The reaction mixture was stirred at room temperature for 2 days, producing a fine colourless precipitate (KCl). The reaction mixture was filtered and volatiles from the filtrate removed *in vacuo* to give essentially 1:2 mixture of *cis*-[R$_3$SiOC(H)=C(H)OSiR$_3$] (R = Me; **3a** and ^{i}Pr **3b**) and (**NON**)Mg(THF). These compounds were easily separated by vacuum distillation – distilling off **3a/3b** as colourless oils and leaving (**NON**)Mg(THF) as a colourless precipitate. (**NON**)Mg(THF) is the starting material to make the hydride **1-Mg** and therefore is recyclable.

General preparation of 3a/3b from 2-Ca. To a solution of **2-Ca** (0.200 g, 0.117 mmol) in THF (5 mL) was added the appropriate chlorosilane (0.258 mmol, 2.2 equiv.) at room temperature. The reaction mixture was stirred at room temperature for 1 hour, producing a fine colourless precipitate (KCl). The reaction mixture was filtered and volatiles from the filtrate removed *in vacuo* to give essentially 1:2 mixture of *cis*-[$R_3SiOC(H)=C(H)OSiR_3$] (R = Me; **3a** and ^{i}Pr; **3b**) and (**NON**)Ca(THF)$_2$. These compounds were easily separated from each other by vacuum distillation - distilling off **3a/3b** as colourless oils and leaving (**NON**)Ca(THF)$_2$ as a colourless precipitate. (**NON**)Ca(THF)$_2$ can simply be converted back into the starting material (**NON**)Ca(OEt$_2$)$_2$ as described below.

***cis*-[Me$_3$SiOC(H)=C(H)OSiMe$_3$] (3a). Yield** – 17.6 mg (0.086 mmol, 72%) from **2-Mg** and 20.8 mg (0.102 mmol, 87%) from **2-Ca**. All characterising data matched that previously reported.[S3]

***cis*-[$^{i}Pr_3SiOC(H)=C(H)OSi^{i}Pr_3$] (3b). Yield** – 30.8 mg (0.083 mmol, 69%) from **2-Mg** and 39.7 mg (0.107 mmol, 91%) from **2-Ca**. N.B. the product can be further purified by flash column chromatography (silica) using neat *n*-hexane as the eluent (R_f = 0.3). **^{1}H NMR** (400 MHz, C_6D_6, 298 K): δ = 1.14 (br., 42H, C*H*(CH$_3$)$_2$ and CH(C*H*$_3$)$_2$), 5.57 (s, 2H, OC*H*); **^{13}C{^{1}H} NMR** (101 MHz, C_6D_6): δ = 12.5 (*C*H(CH$_3$)$_2$), 18.0 (CH(*C*H$_3$)$_2$), 125.4 (O*C*H); **^{29}Si{^{1}H} NMR** (79 MHz, C_6D_6): δ = 14.5; **HRMS** (ESI+) *m/z* [M + Na]$^+$ 395.2773 (calcd for $C_{20}H_{44}NaO_2Si_2$, 395.2777).

(NON)Ca(THF)$_2$. Yield - 0.152 g (0.176 mmol, 76%) from the synthesis of **3a** and 0.174 g (0.204 mmol, 87%) from the synthesis of **3b**. N.B. X-ray quality crystals of (**NON**)Ca(THF)$_2$ were grown by dissolving the powder recovered from the synthesis of **3a-b** (discussed above) in the minimum volume of warm benzene (ca. 60 °C) and allowing the solution to slowly cool to room temperature overnight. **^{1}H NMR** (400 MHz, C_6D_6, 298 K): δ = 1.07 (m, 8H, OCH$_2$C*H*$_2$), 1.11 (d, $^{3}J_{HH}$ = 7.0 Hz, 12H, CH(C*H*$_3$)$_2$), 1.34 (d, $^{3}J_{HH}$ = 6.8 Hz, 12H, CH(C*H*$_3$)$_2$), 1.38 (s, 18H, C(C*H*$_3$)$_3$), 1.79 (s, 6H, C(C*H*$_3$)$_2$), 3.17 (m, 8H, OC*H*$_2$CH$_3$), 3.61 (sept., $^{3}J_{HH}$ = 6.9 Hz, 8H, C*H*(CH$_3$)$_2$), 6.21 (d, $^{4}J_{HH}$ = 2.2 Hz, 2H, NON-*o*-C*H*), 6.65 (d, $^{4}J_{HH}$ = 2.2 Hz, 4H, NON-*p*-C*H*), 7.15 (t, $^{3}J_{HH}$ = 7.6 Hz, 2H, Dipp-*p*-C*H*), 7.28 (d, $^{3}J_{HH}$ = 7.6 Hz, 4H, Dipp-*m*-C*H*); **^{13}C{^{1}H} NMR** (101 MHz, C_6D_6): δ = 25.2, 25.4, 25.5 (CH(*C*H$_3$)$_2$ and OCH$_2$*C*H$_2$), 28.1 (*C*H(CH$_3$)$_2$), 31.9 (XA-C(*C*H$_3$)$_2$), 32.2 (C(*C*H$_3$)$_3$), 35.0 (*C*(CH$_3$)$_3$), 35.4 (XA-*C*(CH$_3$)$_2$), 69.1 (O*C*H$_2$CH$_3$), 103.3, 107.6, 122.6, 123.7, 128.6, 129.5, 139.2, 145.4, 146.4, 148.9, 149.7 (Ar-*C*); **IR** υ/cm^{-1} (Nujol): 2961(s), 2867(m), 1623(s), 1515(s), 1500(s), 1462(s), 1442(s), 1393(s), 1383(s), 1361(s), 1334(m), 1298(m), 1256(m), 1217(s), 1142(m), 1117(m), 1099(m), 1057(m), 1015(m), 934(m), 845(m), 802(m), 772(m), 759(m), 741(m), 656(m); **Elemental Analysis** – calc. for $C_{55}H_{78}CaN_2O_3$: C 77.24%, H 9.19%, N 3.38%; found: C 77.52%; H 9.22%, N 3.54%.

Reforming (NON)Ca(OEt$_2$)$_2$. The biproduct (**NON**)Ca(THF)$_2$ can simply be turned back into the calcium-containing starting material (**NON**)Ca(OEt$_2$)$_2$ by heating the solid to 200 °C under high vacuum (ca. 5 x 10^{-2}

mbar) for 2 hours, which gives a pale yellow powder. This powder is then dissolved in the minimum volume of Et_2O giving colourless solution. Removal of the solvent gives essentially quantitative yields of (**NON**)Ca(OEt_2)$_2$ as a colourless powder. For example, performing this transformation on a 0.250 g scale of (**NON**)Ca(THF)$_2$ using 5 mL of Et_2O gives 0.240 g of (**NON**)Ca(OEt_2)$_2$ (96% yield). All characterising data matched that previously reported.[S2]

Preparation of $^iPr_3SiOCH_2CH(Cl)OSi^iPr_3$ (4). To a solution of **3b** (50.0 mg, 0.134 mmol) in benzene (1 mL) was added a 2.0 M HCl in Et_2O solution (0.20 mL, 0.40 mmol) at room temperature. The reaction was sealed and stirred at room temperature overnight. Volatiles were removed *in vacuo* to give compound **4** as a colourless oil. **Yield** – 53.8 mg (0.132 mmol, 98%). **^{1}H NMR** (400 MHz, C_6D_6, 298 K): δ = 0.98-1.15 (m., 42H, C*H*(CH_3)$_2$ and CH(C*H*$_3$)$_2$), 4.04 (m., 2H, OC*H*$_2$), 6.03 (dd, $^3J_{HH}$ = 6.0, 4.4 Hz, 1H, OC*H*Cl); **^{13}C{^{1}H} NMR** (176 MHz, C_6D_6): δ = 12.3, 12.4 (*C*H(CH_3)$_2$), 18.0, 18.1 (CH(*C*H$_3$)$_2$), 70.7 (O*C*H$_2$) 91.4 (O*C*HCl); **^{29}Si{^{1}H} NMR** (139 MHz, C_6D_6): δ = 14.2, 18.3 **HRMS** (EI+) *m/z* [M – HCl]$^+$ 372.2871 (calcd for $C_{20}H_{44}O_2Si_2$, 372.2880).

Preparation of $^iPr_3SiOCH(Br)CH(Br)OSi^iPr_3$ (5). To a solution of **3b** (50.0 mg, 0.134 mmol) in benzene (1 mL) was added Br_2 (25.7 mg, 0.161 mmol) at room temperature. The reaction was sealed and stirred at room temperature overnight. Volatiles were removed *in vacuo* to give compound **5** as a colourless oil. **Yield** – 68.6 mg (0.127 mmol, 96%). **^{1}H NMR** (400 MHz, C_6D_6, 298 K): δ = 0.94-1.26 (m., 42H, C*H*(CH_3)$_2$ and CH(C*H*$_3$)$_2$), 6.53 (s, 2H, OC*H*Br); **^{13}C{^{1}H} NMR** (101 MHz, C_6D_6): δ = 12.4 (*C*H(CH_3)$_2$), 18.1 (CH(*C*H$_3$)$_2$), 88.0, 89.5 (O*C*HBr); **^{29}Si{^{1}H} NMR** (79 MHz, C_6D_6): δ = 20.2, 21.0; **HRMS** (EI+) *m/z* [^{i}Pr$_3$SiOH]$^+$ 174.1424 (calcd for $C_9H_{22}OSi$, 174.1440).

^{1}H and ^{13}C NMR spectra of new compounds

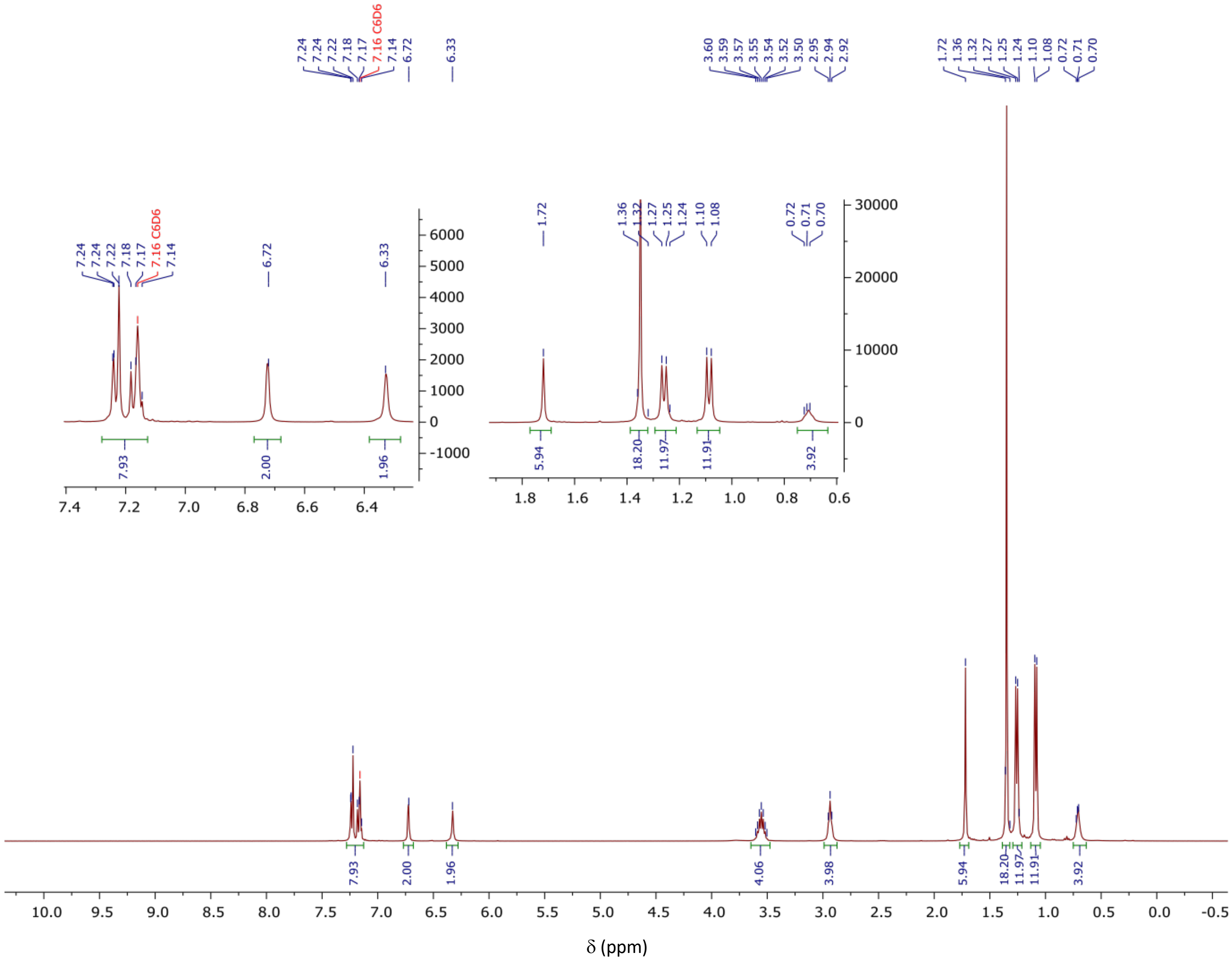

Figure S1: ^{1}H NMR spectrum of (**NON**)Mg(THF) (400 MHz, C_6D_6, 298 K)

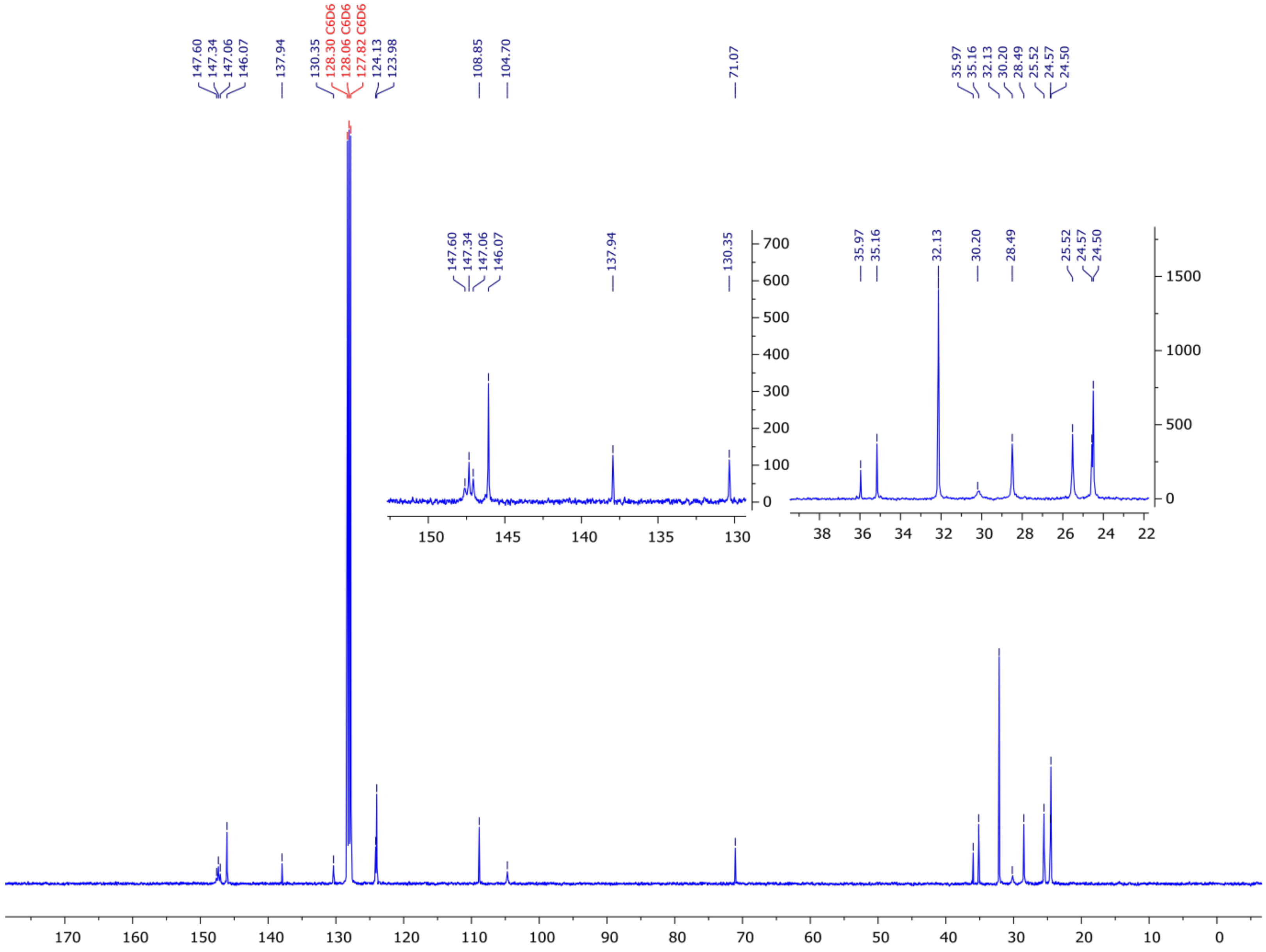

Figure S2: ^{13}C NMR spectrum of (**NON**)Mg(THF) (400 MHz, C_6D_6, 298 K)

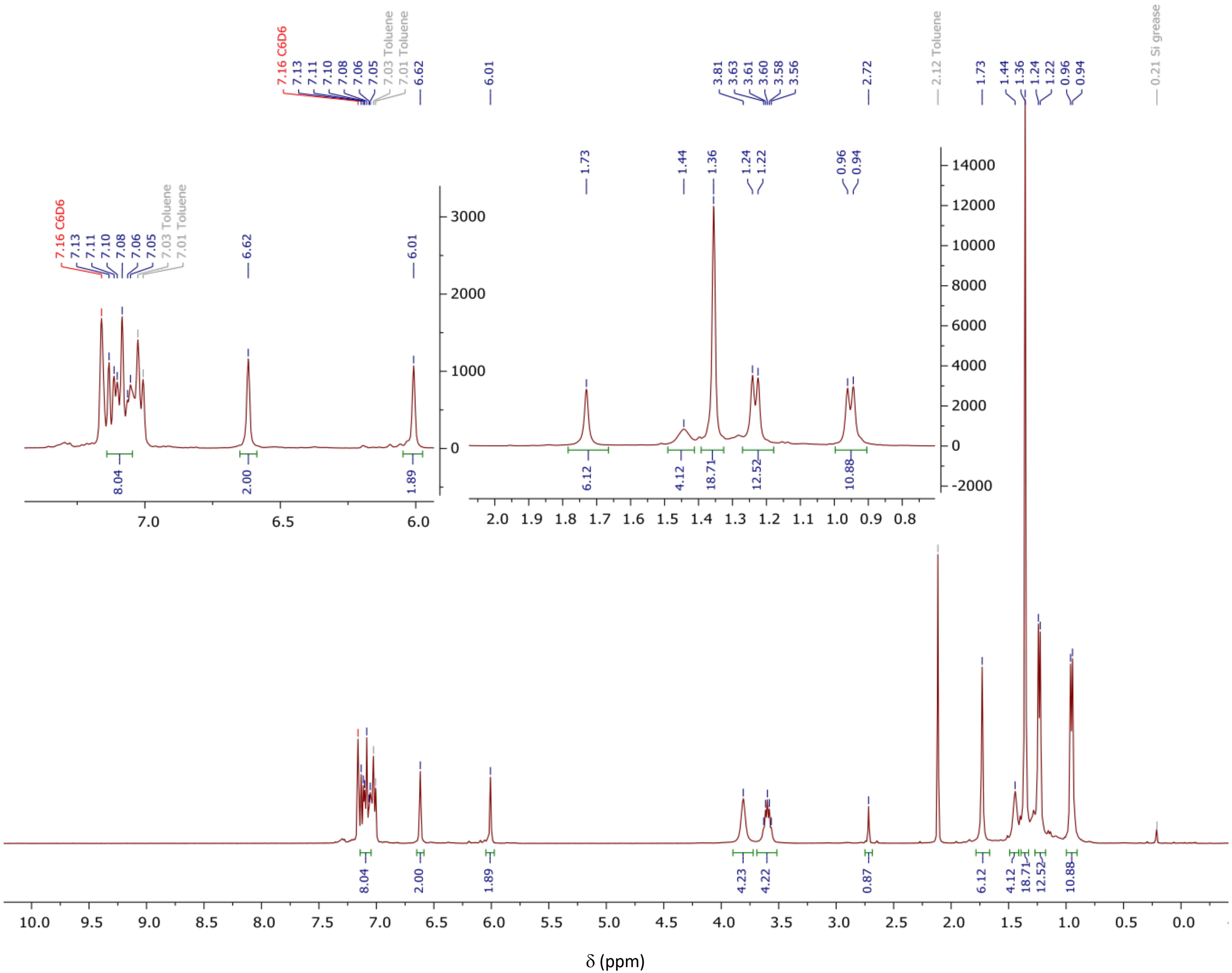

Figure S3: ^{1}H NMR spectrum of K_2[(**NON**)MgH(THF)]$_2$ (**1-Mg**) (400 MHz, C_6D_6, 298 K)

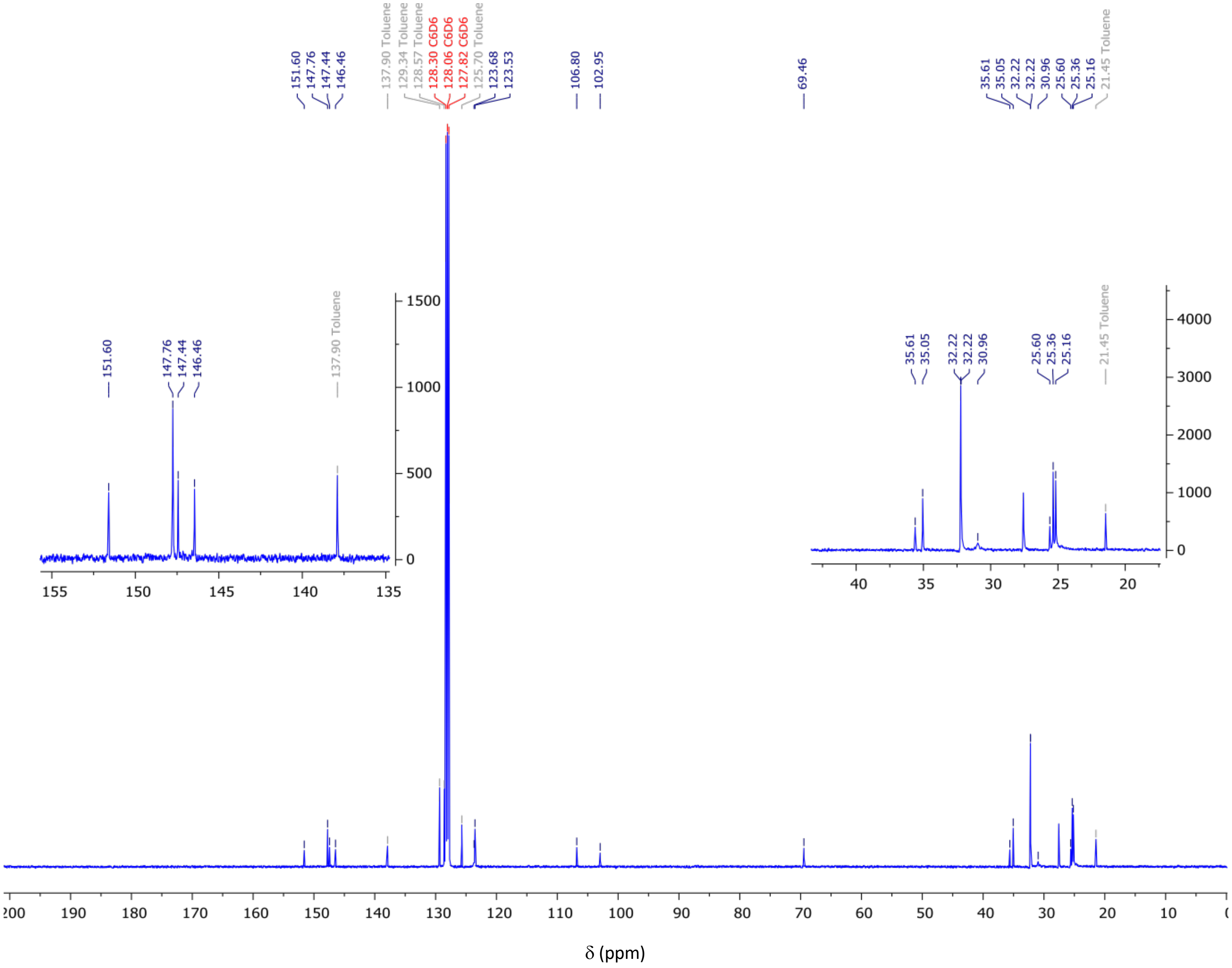

Figure S4: ^{13}C NMR spectrum of $K_2[(\mathbf{NON})MgH(THF)]_2$ (**1-Mg**) (400 MHz, C_6D_6, 298 K)

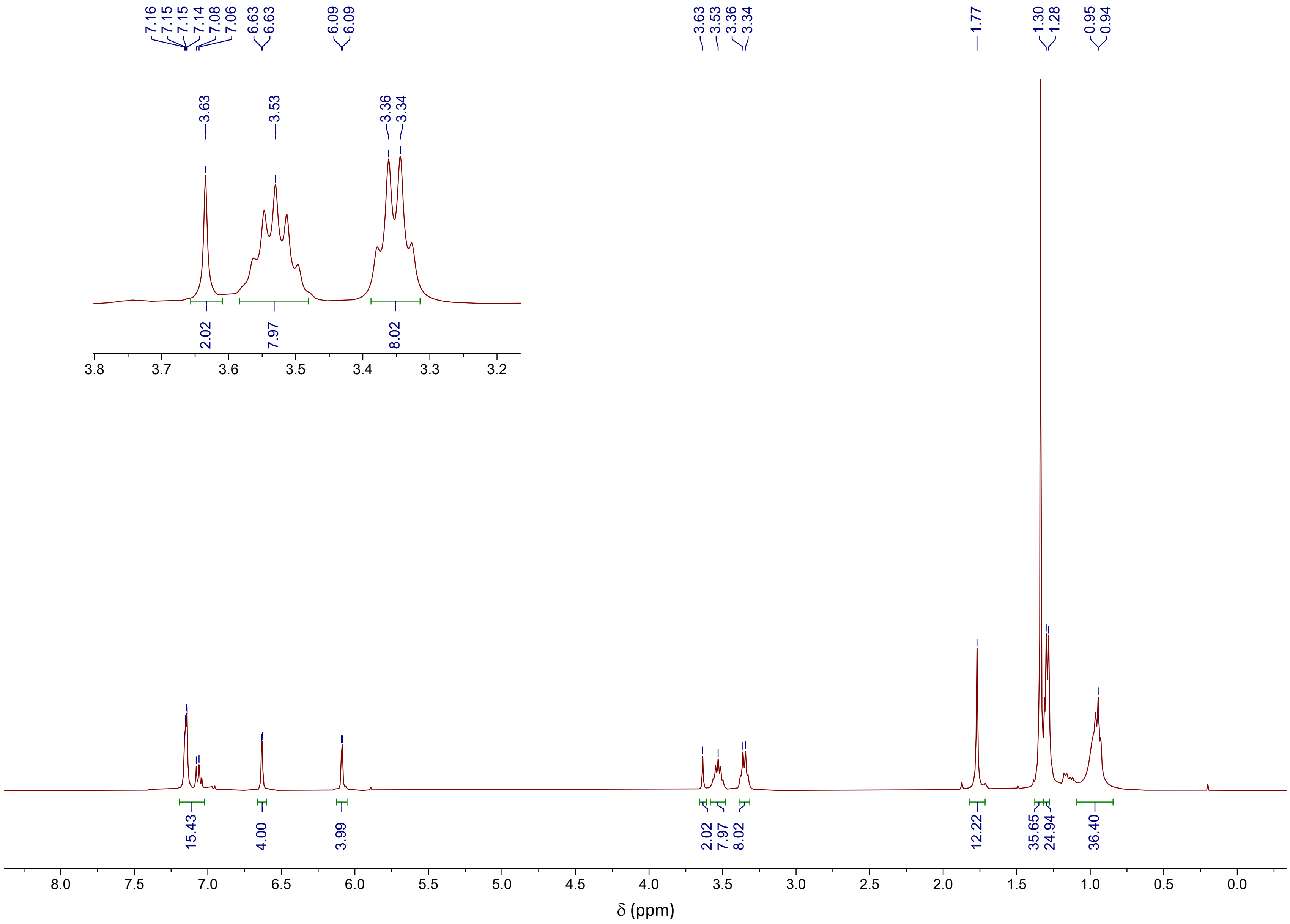

Figure S5: ^{1}H NMR spectrum of $K_2[(\mathbf{NON})CaH(OEt_2)]_2$ (**1-Ca**) (400 MHz, C_6D_6, 298 K)

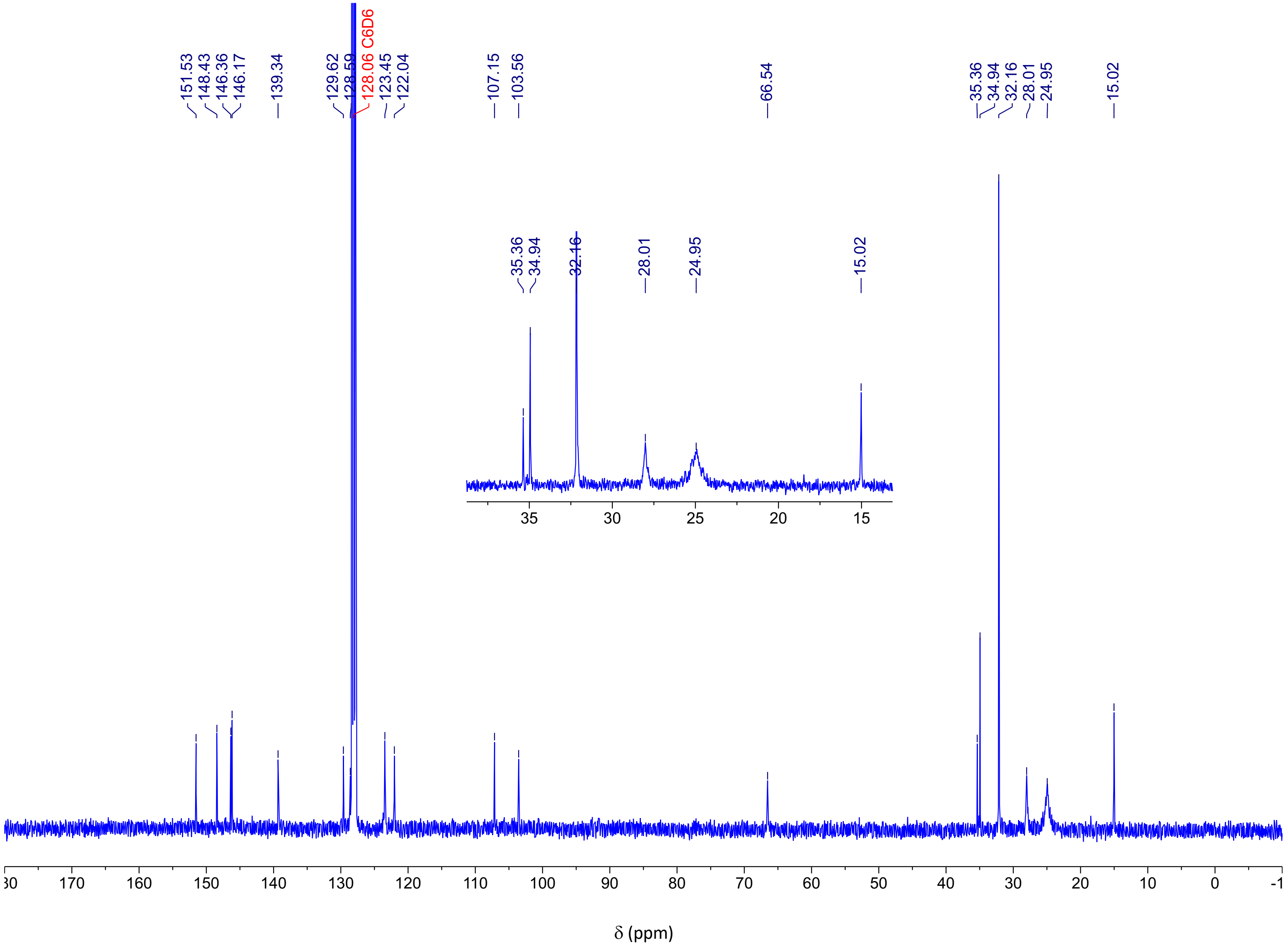

Figure S6: ^{1}H NMR spectrum of K_2[(**NON**)CaH(OEt_2)]$_2$ (**1-Ca**) (400 MHz, C_6D_6, 298 K)

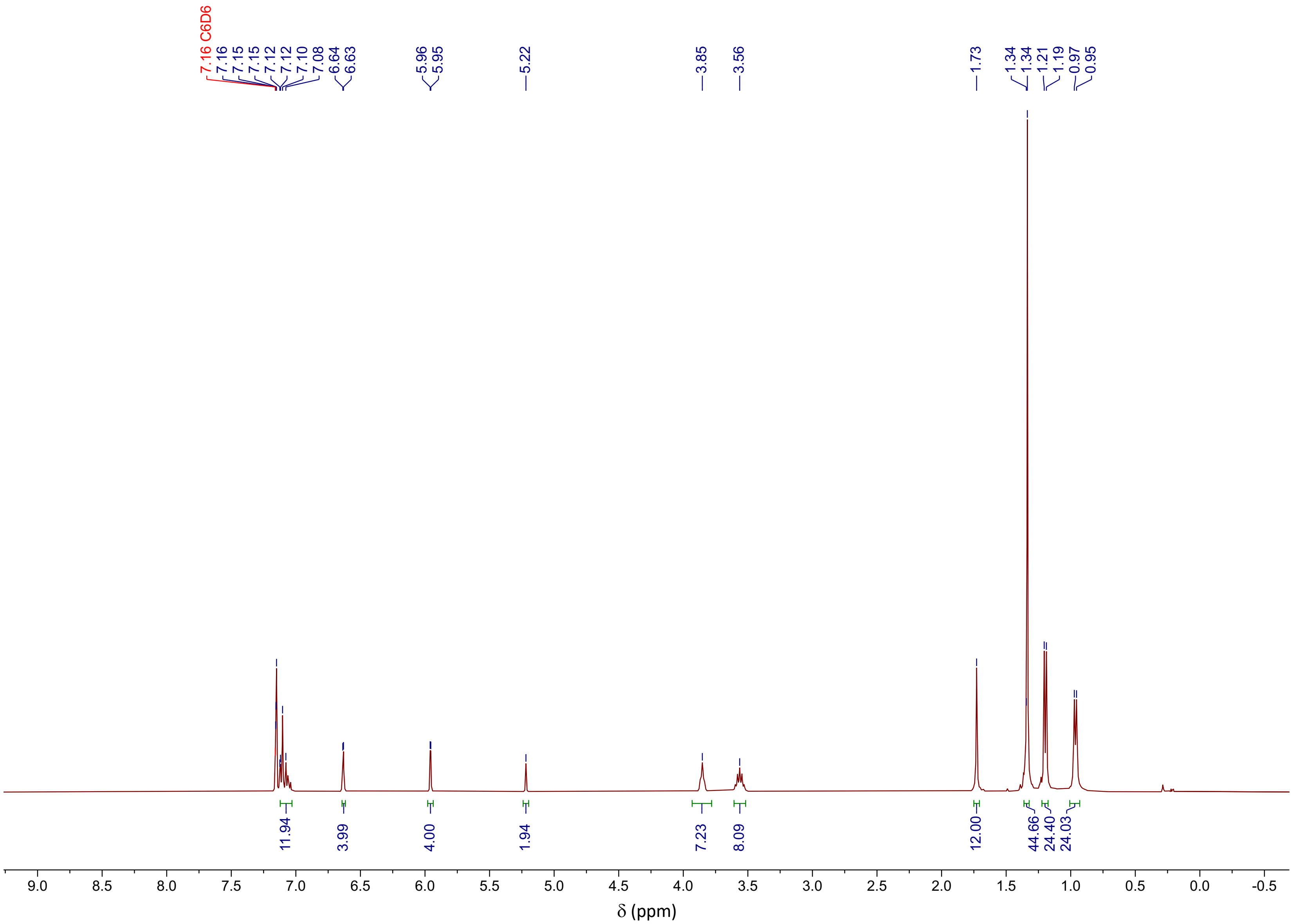

Figure S7: ^{1}H NMR spectrum of $K_2[\{(\mathbf{NON})Mg(THF)\}_2O_2C_2H_2]$ (**2-Mg**) (400 MHz, C_6D_6, 298 K)

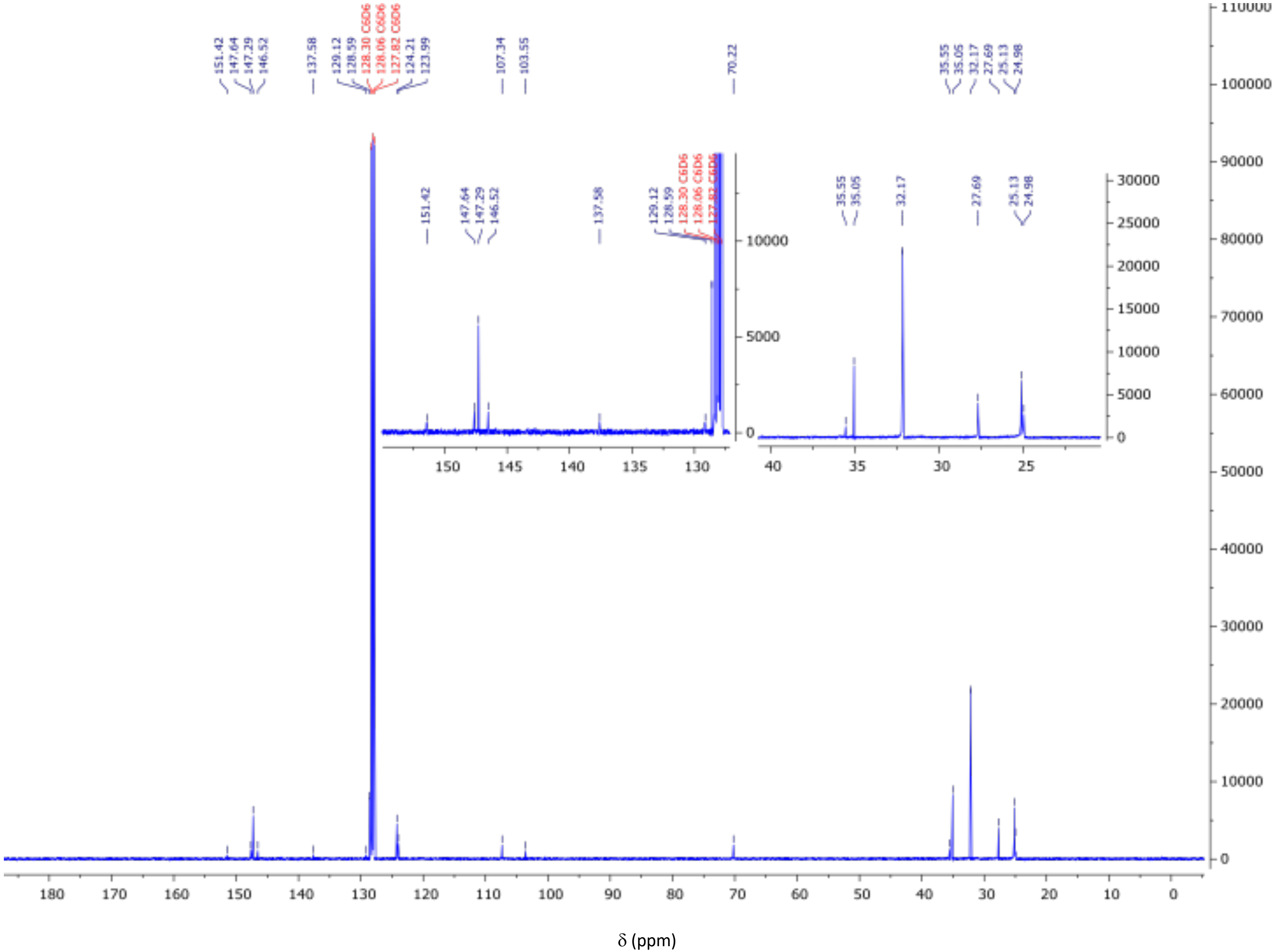

Figure S8: ^{1}H NMR spectrum of $K_2[\{(\mathbf{NON})Mg(THF)\}_2O_2C_2H_2]$ (**2-Mg**) (400 MHz, C_6D_6, 298 K)

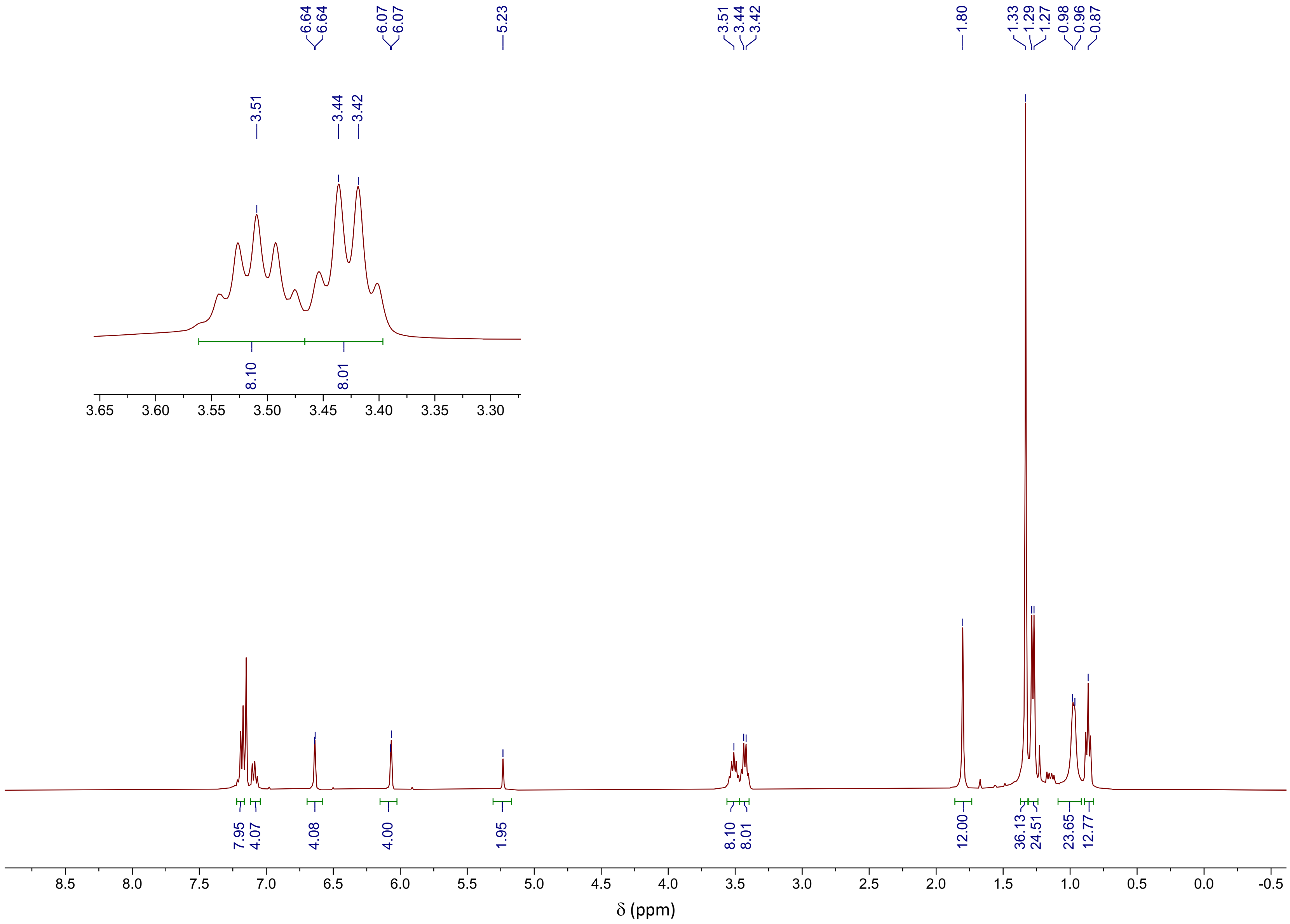

Figure S9: ^{1}H NMR spectrum of $K_2[\{(\mathbf{NON})Ca(OEt_2)\}_2O_2C_2H_2]$ (**2-Ca**) (400 MHz, C_6D_6, 298 K)

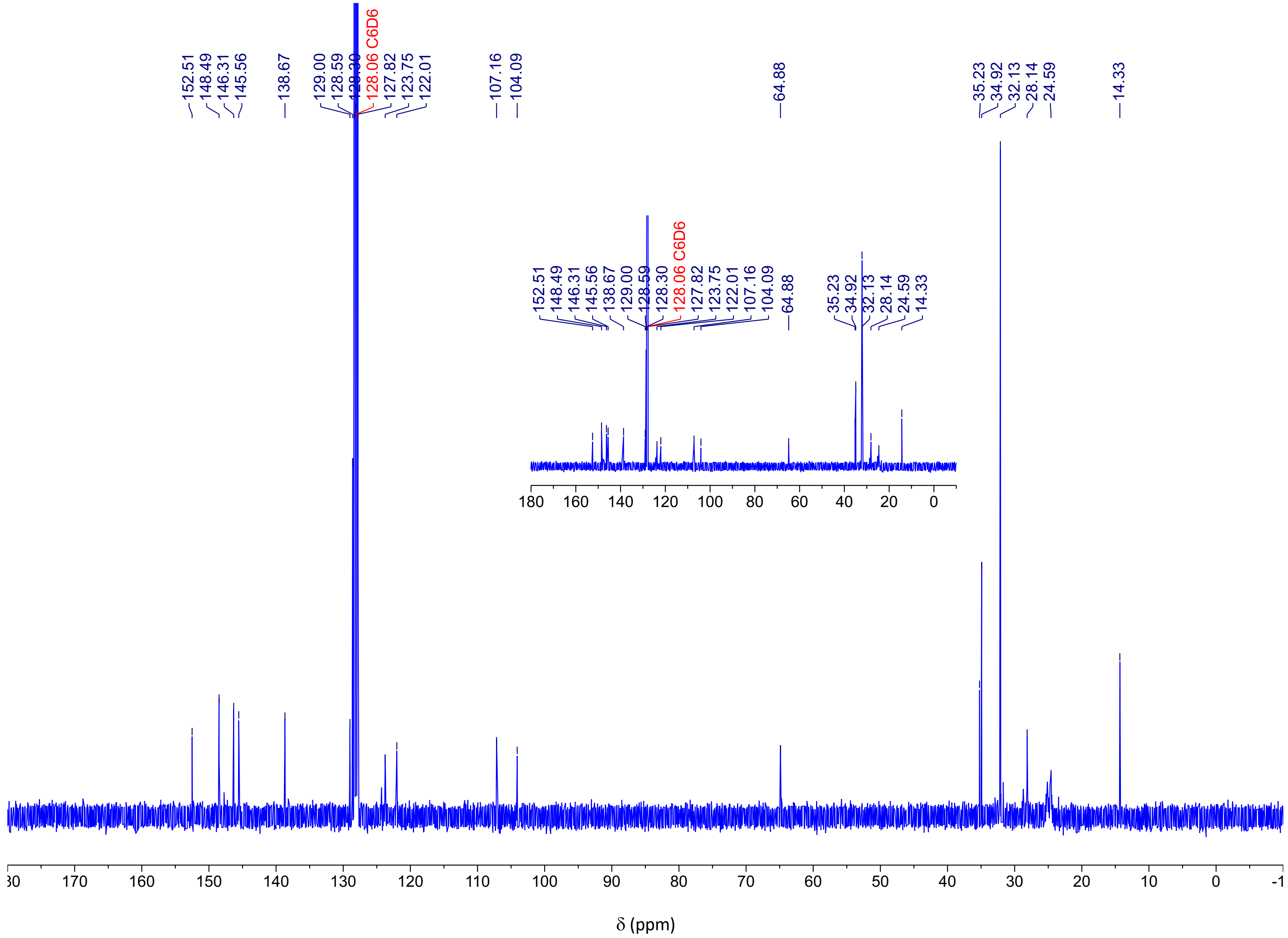

Figure S10: ^{13}C NMR spectrum of $K_2[\{(\mathbf{NON})Ca(OEt_2)\}_2O_2C_2H_2]$ (**2-Ca**) (400 MHz, C_6D_6, 298 K)

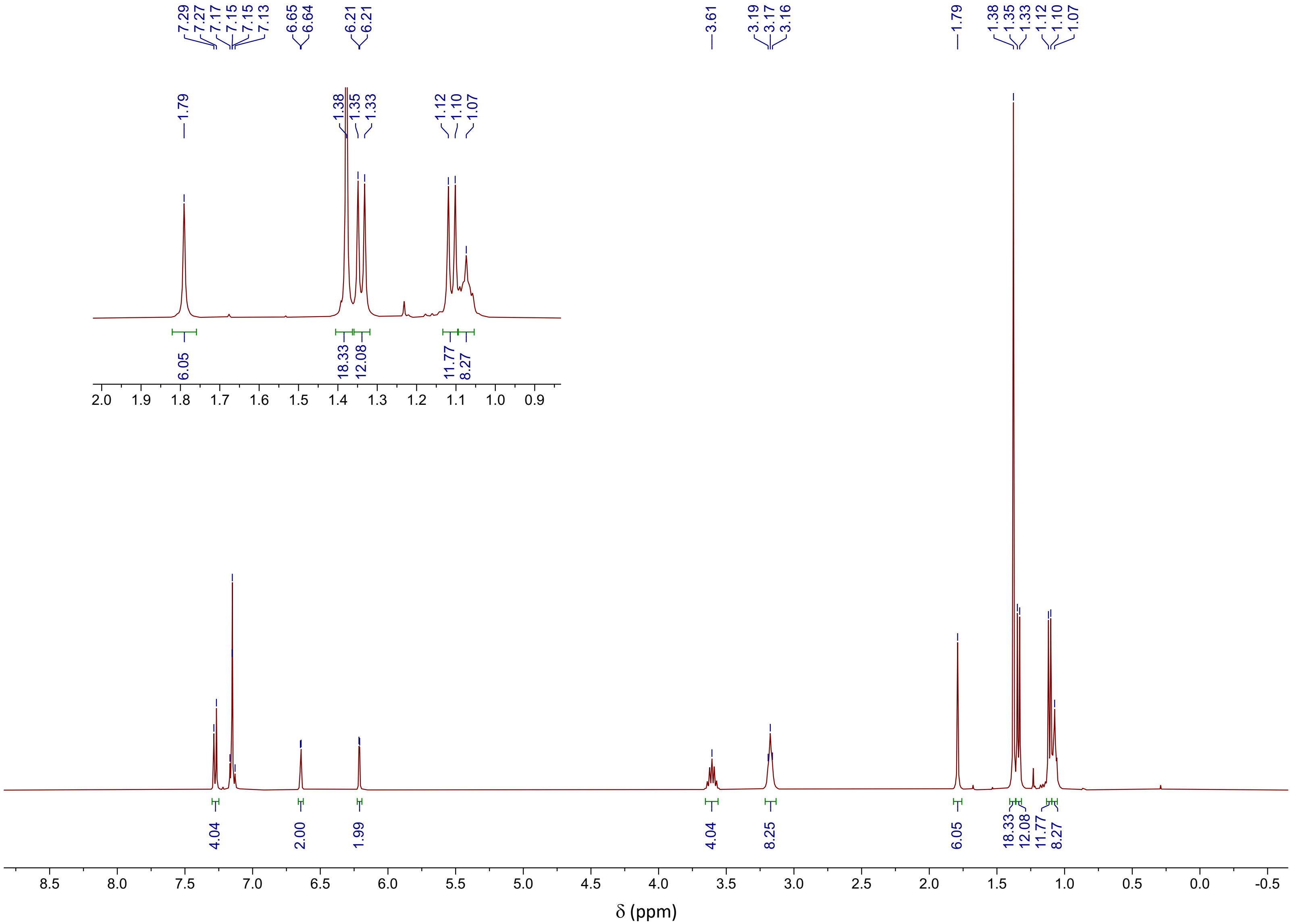

Figure S11: ^{1}H NMR spectrum of (**NON**)Ca(THF)$_2$ (400 MHz, C_6D_{12}, 298 K)

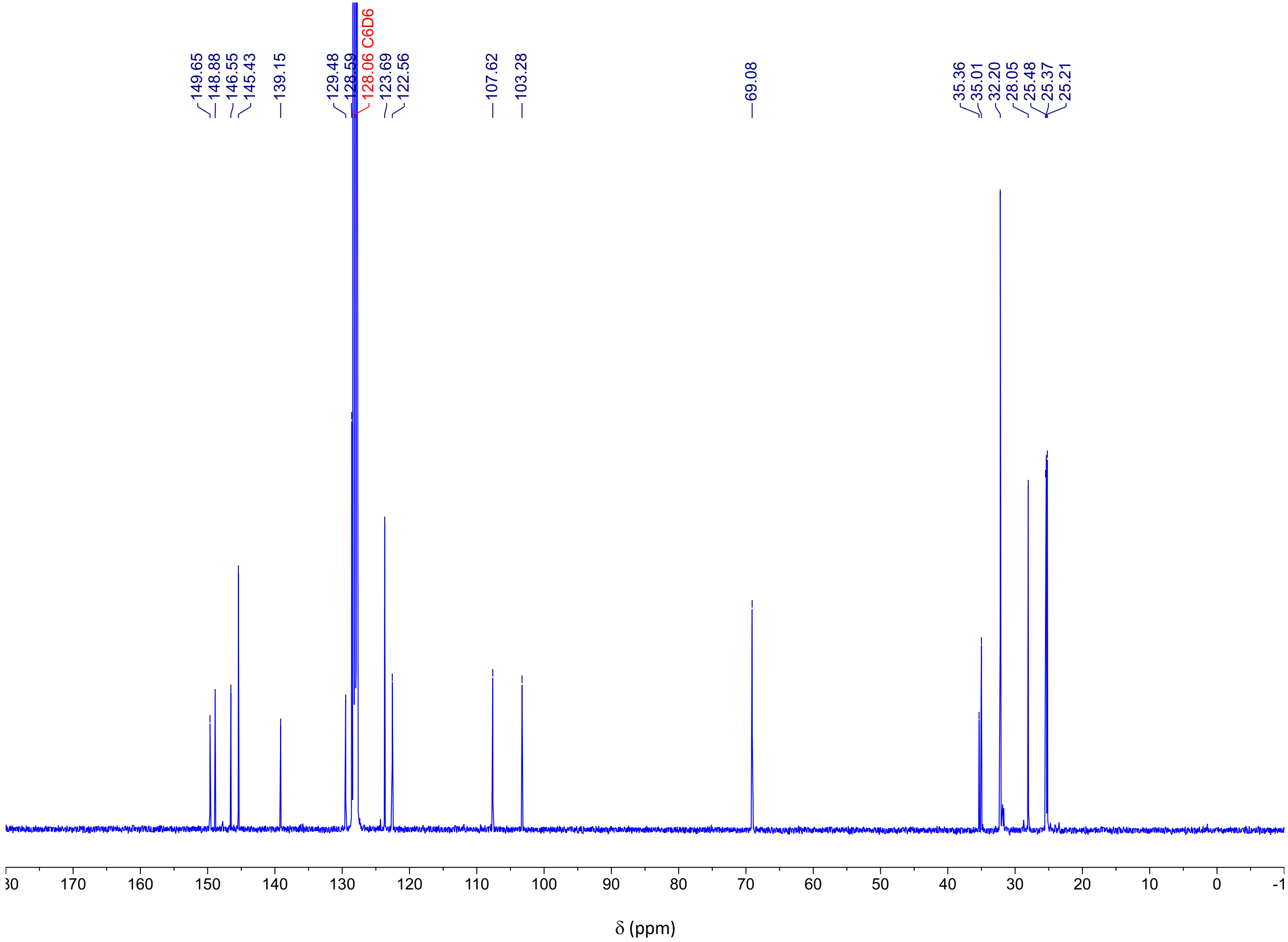

Figure S12: ^{13}C NMR spectrum of (**NON**)Ca(THF)$_2$ (400 MHz, C_6D_{12}, 298 K)

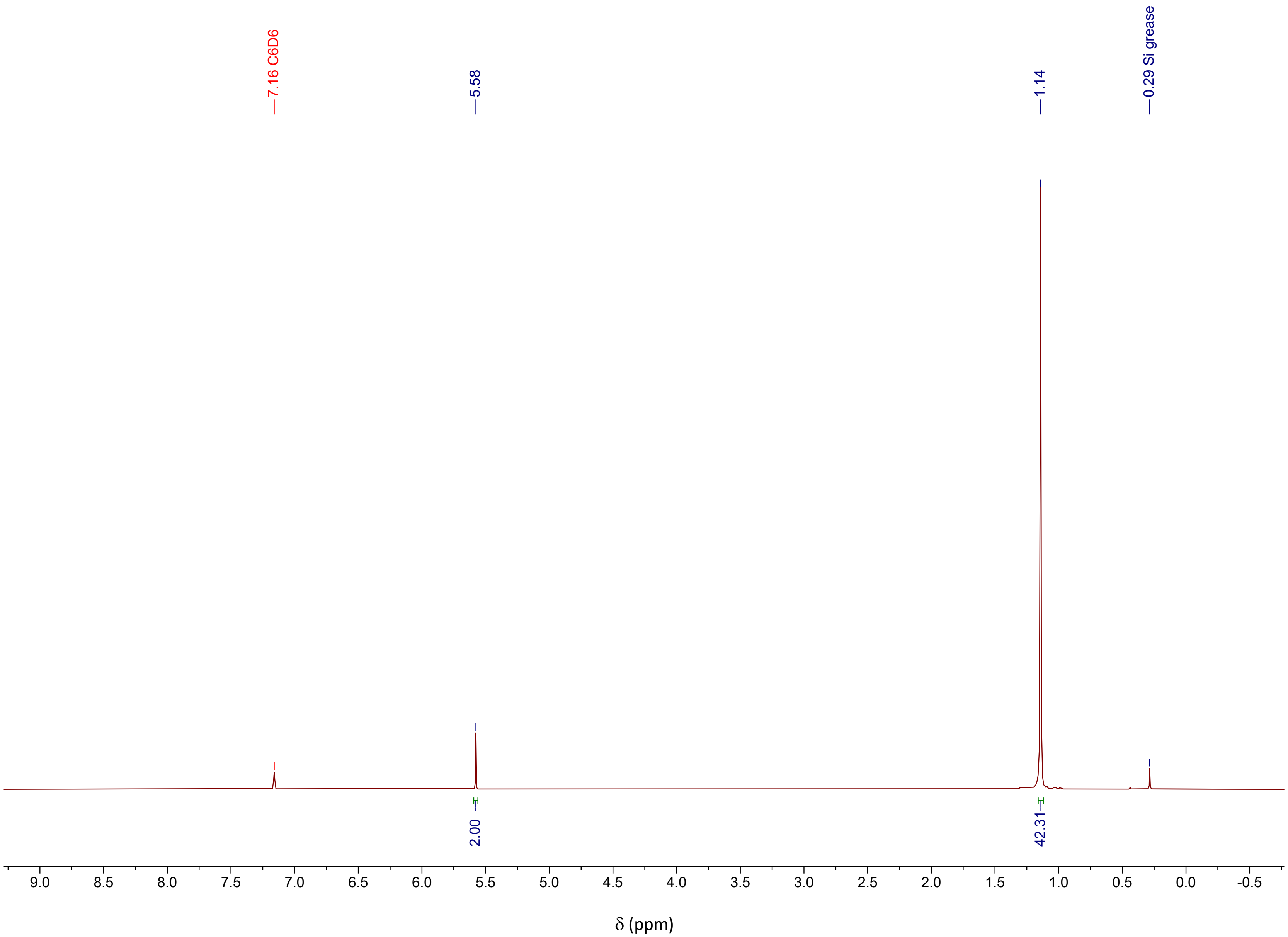

Figure S13: ^{1}H NMR spectrum of *cis*-[$^{i}Pr_3SiOC(H)=C(H)OSi^{i}Pr_3$] (**3b**) (400 MHz, C_6D_6, 298 K)

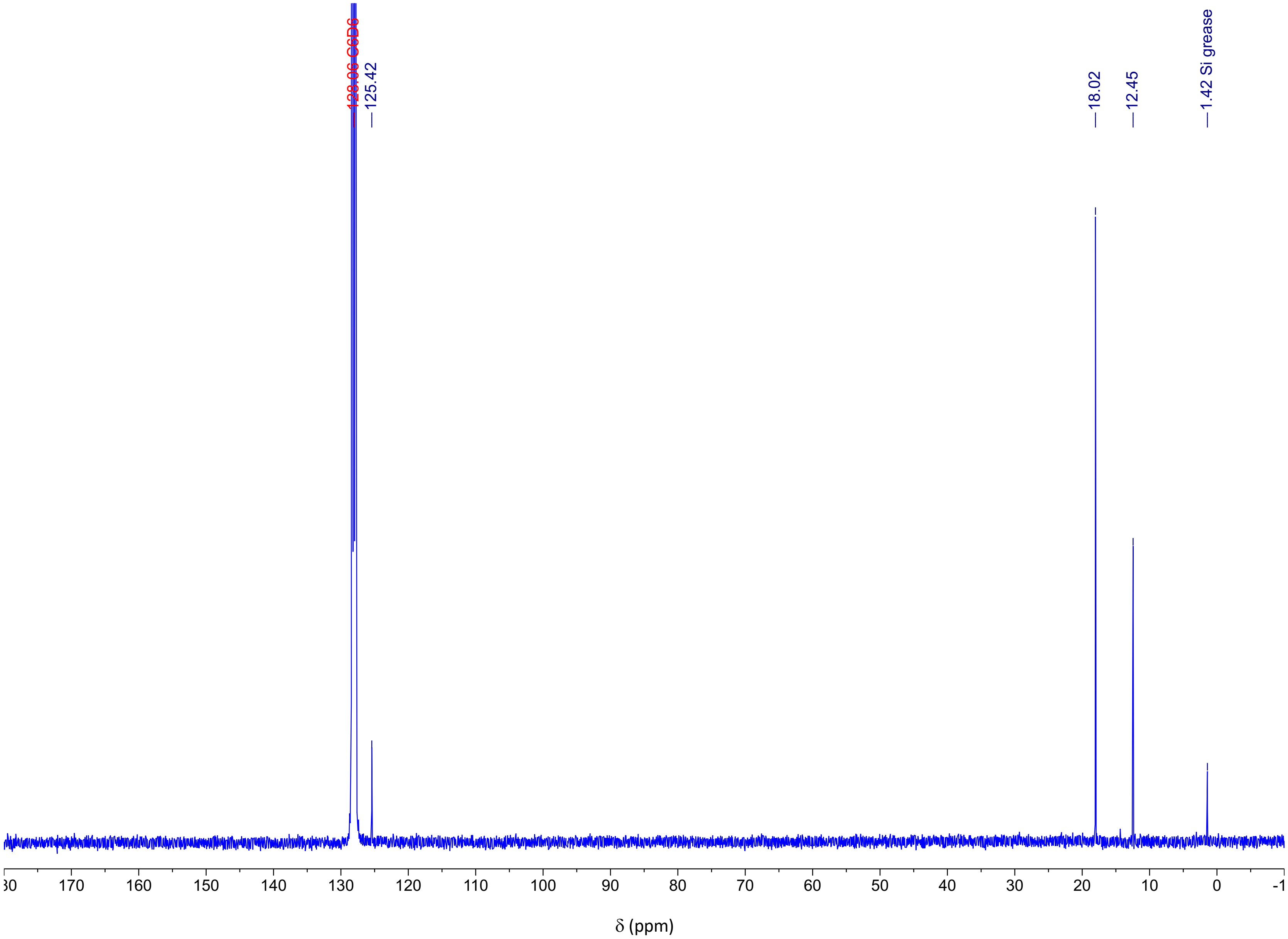

Figure S14: ^{13}C NMR spectrum of *cis*-[^{i}Pr$_3$SiOC(H)=C(H)OSi^{i}Pr$_3$] (**3b**) (400 MHz, C_6D_6, 298 K)

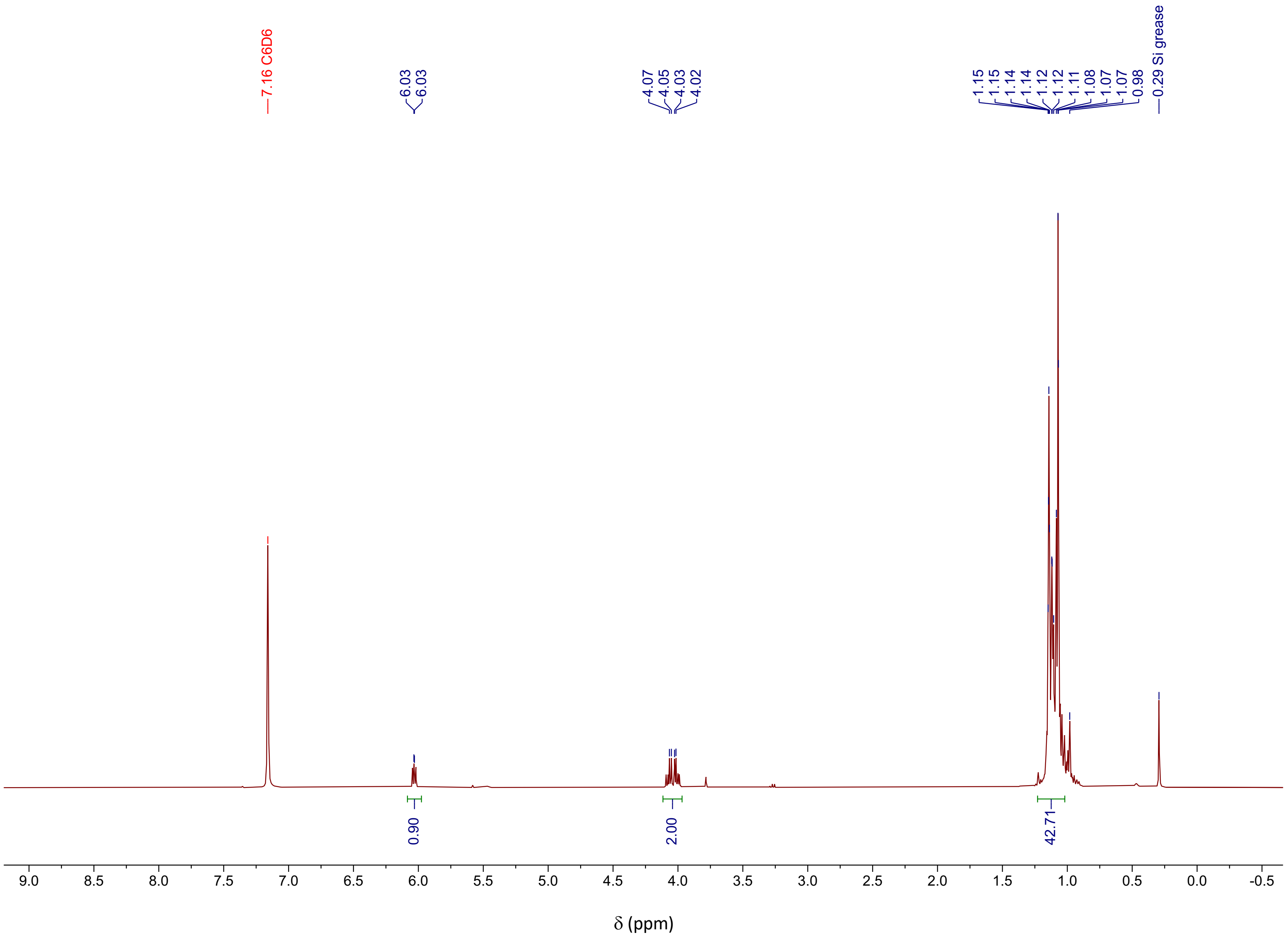

Figure S15: ^{1}H NMR spectrum of [$^{i}Pr_3SiOCH_2CH(Cl)OSi^{i}Pr_3$] (**4**) (400 MHz, C_6D_6, 298 K)

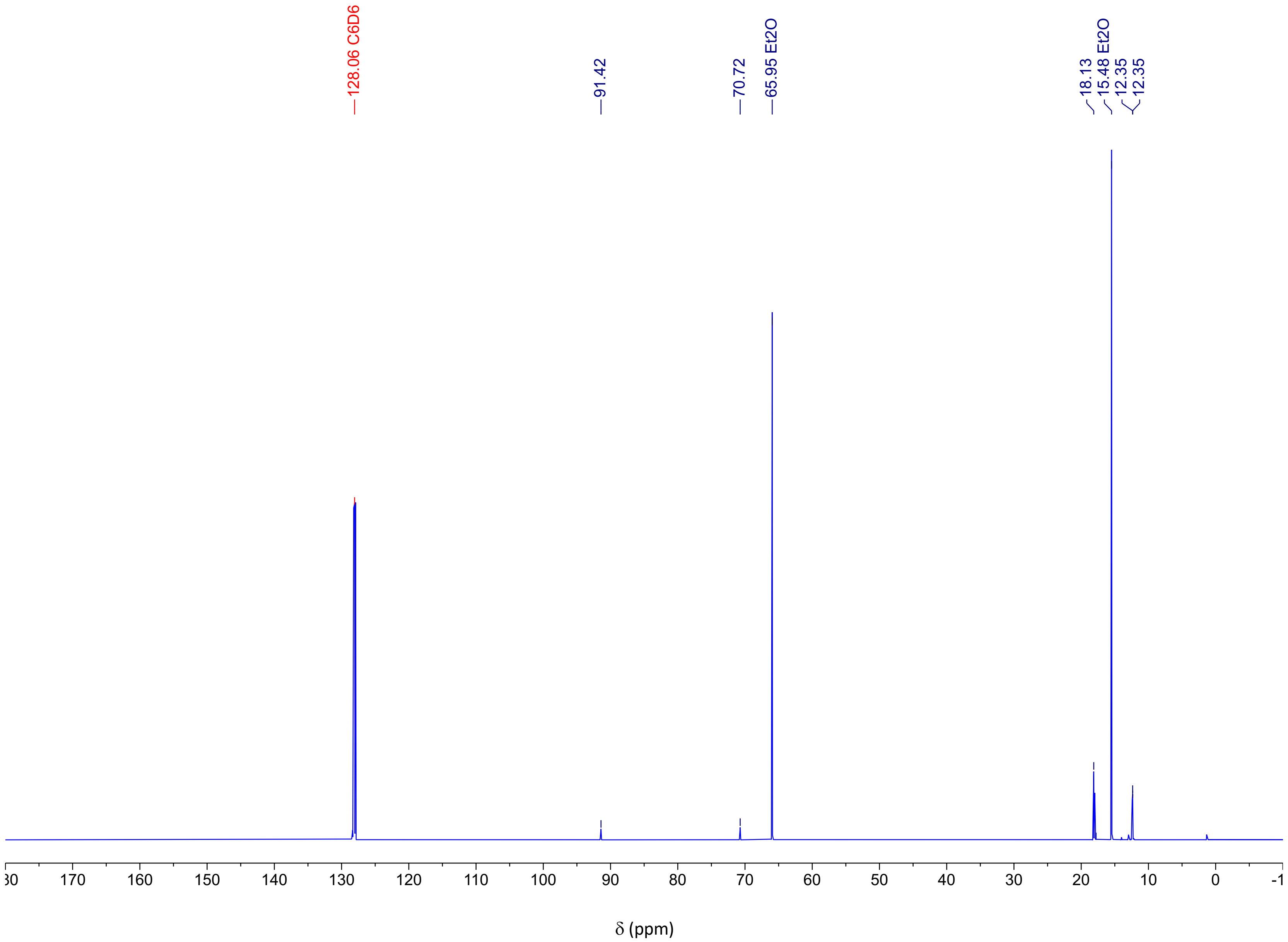

Figure S16: ^{13}C NMR spectrum of [^{i}Pr$_3$SiOCH$_2$CH(Cl)OSi^{i}Pr$_3$] (**4**) (400 MHz, C_6D_6, 298 K)

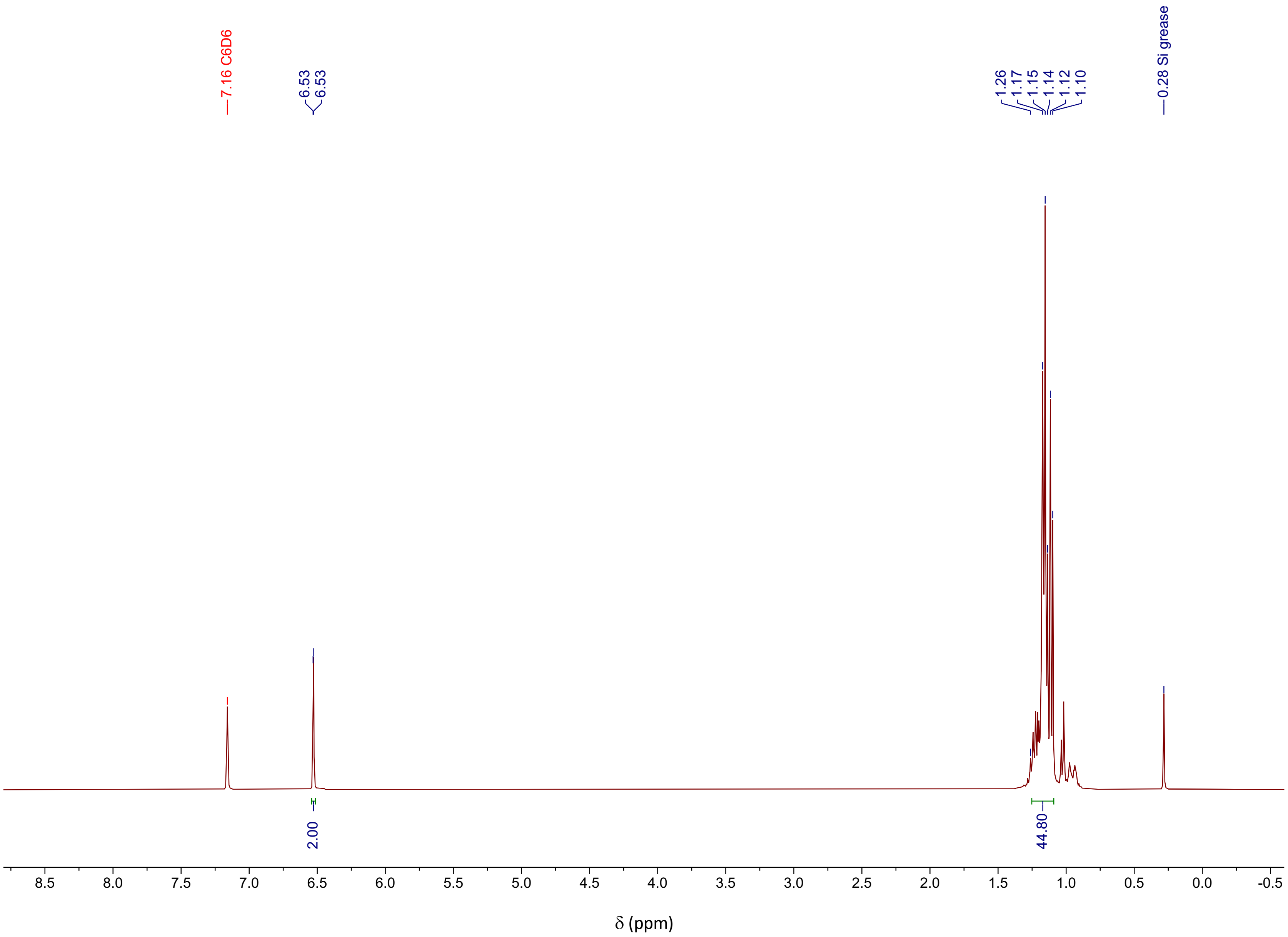

Figure S17: ^{1}H NMR spectrum of [$^{i}Pr_3$SiOCH(Br)CH(Br)OSi$^{i}Pr_3$] (**5**) (400 MHz, C_6D_6, 298 K)

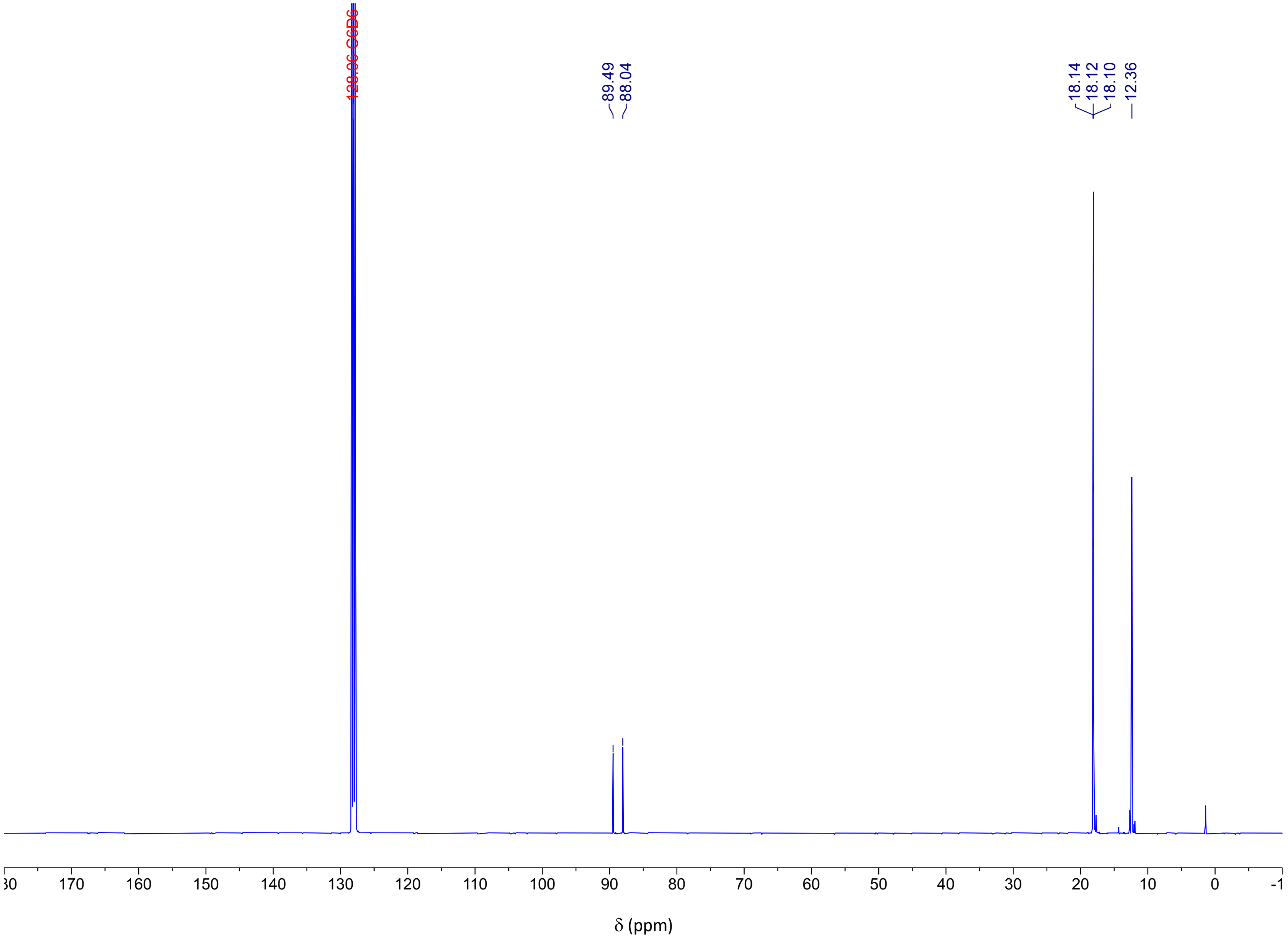

Figure S18: ^{13}C NMR spectrum of [^{i}Pr$_3$SiOCH(Br)CH(Br)OSi^{i}Pr$_3$] (**5**) (400 MHz, C_6D_6, 298 K)

Diffusion ordered NMR spectroscopy (DOSY) experimental details

Diffusion Ordered Spectroscopy (DOSY) experiments were carried out on a Bruker Avance 400 MHz spectrometer at 298 K. Compounds **1-Mg** and **1-Ca** were analysed at a 20 mg/mL concentration in C_6D_6. To avoid distorted diffusion coefficients, the spectra were collected without sample spinning. The Bruker dstebpgp3s convection corrected pulse sequence was used, with a diffusion delay of Δ = 100 ms and gradient pulse length of δ = 2 ms. Spectra were obtained over a 16 step gradient range from 2-95%. Spectra were processed using the MestReNova Bayesian DOSY transform function at a resolution factor of 0.1 and 128 points in the diffusion dimension over a range of $1x10^{-11} – 1x10^{-8}$ m^2s^{-1} as shown in Figure S19.

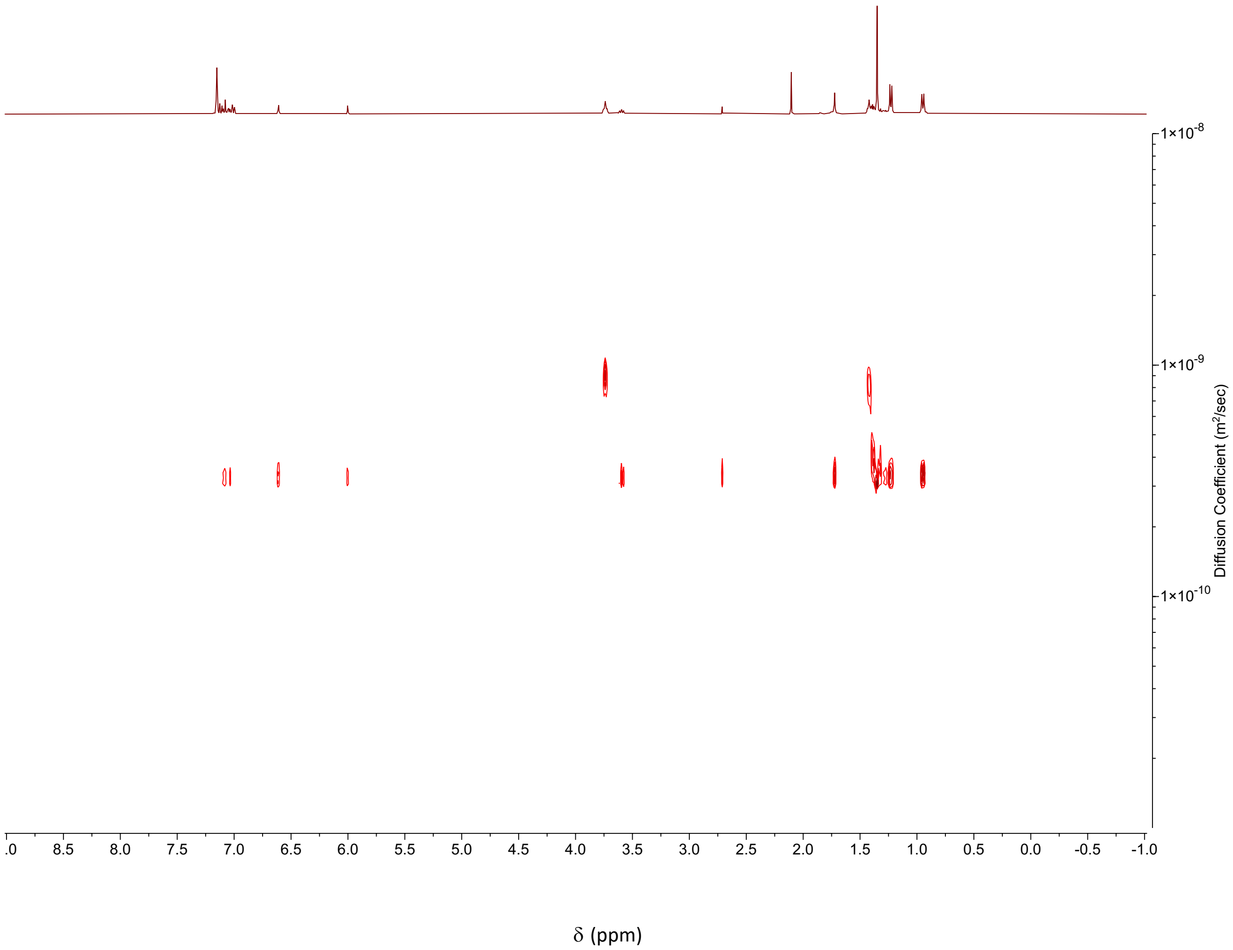

Figure S19: Processed ^{1}H NMR data of **1-Mg** in C_6D_6 using the Bayesian DOSY transform function.

The hydrodynamic radii (r_H) of **1-Mg** (9.6 Å) and **1-Ca** (9.6 Å) were calculated using the Stokes–Einstein equation (for diffusion of spherical particles through a liquid with low Reynolds number) shown below.

$$r_H = \frac{k_B T}{6\pi f \eta D}$$

$k_B = Boltzmann\ constant, 1.38064 \times 10^{-23}\ m^2\ kg\ s^{-2}\ K^{-1}$
$T = Temperature, 298\ K$
$f = shape\ correct\ factor, 1\ for\ a\ spherical\ particle$
$\eta = viscosity\ of\ solvent, 0.000601\ N\ s\ m^2\ (C_6H_6)$
$D = diffusion\ coefficient, 3.77(5)\ m^2 s^{-1}\ for\ \mathbf{1-Mg}$ and $3.86(5)\ m^2 s^{-1}\ for\ \mathbf{1-Ca}$

X-ray crystallographic studies

Single-crystal X-ray diffraction data were collected using either using a Rigaku Supernova dual-source diffractometer (compounds (**NON**)Mg(THF), **2-Mg**, **2-Ca** and (**NON**)Ca$(THF)_2$) or a Rigaku Xcaliber single-source (Mo) diffractometer (compounds **1-Mg** and **1-Ca**). Crystals were selected under Paratone-N oil, mounted on Micromount loops and quench-cooled using an Oxford Cryosystems open flow N_2 cooling device.[S4] Data were collected at 150 K (or 240 K for (**NON**)Ca$(THF)_2$) using mirror monochromated Cu K_α (λ = 1.5418 Å) or Mo K_α (λ = 0.71073 Å) radiation. Data collected were processed using the CrysAlisPro package, including unit cell parameter refinement and inter-frame scaling (which was carried out using SCALE3 ABSPACK within CrysAlisPro).[S5] Equivalent reflections were merged and diffraction patterns processed with the CrysAlisPro suite.[S5] Structures were subsequently solved using SHELXT-2018 and refined on F^2 using the SHELXL 2018 package and the graphical interface Olex2.[S6-S8]

Finalised CIFs for all X-ray diffraction structures (2124995-2125000) have been deposited at the Cambridge Crystallographic Data Centre. These can be obtained free-of-charge via www.ccdc.cam.ac.uk/data_request/cif, by emailing data_request@ccdc.cam.ac.uk, or by contacting The Cambridge Crystallographic Data Centre, 12 Union Road, Cambridge CB2 1EZ, UK; fax: +44 1223 336033.

Table S1: Crystallographic and refinement parameters for the structures of compounds (**NON**)Mg(THF), **1-Mg** and **1-Ca**.

	(**NON**)Mg(THF)·C_6H_6	**1-Mg**·2C_6H_6	**1-Ca**·6C_6H_6
Formula	$C_{57}H_{76}MgN_2O_2$	$C_{114}H_{154}K_2Mg_2N_4O_4$	$C_{138}H_{182}Ca_2K_2N_4O_4$
M	845.50	1771.22	2119.23
Cell Setting	Monoclinic	Monoclinic	Monoclinic
Space Group	$P2_1$	$I2/a$	$P2_1/c$
a/Å	11.7665(3)	20.5581(6)	14.0219(6)
b/Å	16.5973(4)	13.6712(4)	23.4792(10)
c/Å	13.1985(2)	37.5081(8)	20.5061(11)
α/°	90	90	90
β/°	94.614(2)	91.172(3)	109.824(6)
γ/°	90	90	90
V/$Å^3$	2569.21(10)	10539.6(5)	6351.0(6)
Z	2	4	2
Unique/$I > 2\sigma I$	10103/9467	12681/ 8612	15423/11240
R_{int}	0.0227	0.0370	0.0297
Parameters	680	627	756
R_1 (all data/ $I > 2\sigma I$)	0.0444/0.0413	0.0947/0.0566	0.0802/0.0526
wR_2 (all data/ $I > 2\sigma I$)	0.1094 0.1069	0.1316/0.1116	0.1377/ 0.1212
GooF	1.038	1.017	1.030
Residual max/min	0.515/-0.303	0.795 -0.295	0.934/-0.339
T/K	150.0(1)	150.0(1)	150.0(1)
Radiation, λ (Å)	Cu Kα, (1.54184)	Mo Kα, (0.71073)	Mo Kα, (0.71073)
CCDC number	2124995	2124996	2124997

Table S2: Crystallographic and refinement parameters for the structures of compounds **2-Mg**, **2-Ca** and (**NON**)Ca(THF)$_2$.

	**2-Mg**·8C_6H_6	**2-Ca**·8C_6H	(**NON**)Ca(THF)$_2$·C_6H_6
Formula	$C_{152}H_{190}K_2Mg_2N_4O_6$	$C_{152}H_{194}Ca_2K_2N_4O_6$	$C_{61}H_{84}$CaN2O3
M	2295.89	2331.46	933.38
Cell Setting	Monoclinic	Monoclinic	Monoclinic
Space Group	*C*2/c	*P*2$_1$/*c*	*P*2$_1$/*c*
a/Å	27.659(3)	34.2302(4)	13.36760(10)
b/Å	16.827(3)	14.4593(2)	21.7975(2)
c/Å	28.842(3)	14.4593(2)	19.7244(2)
α/°	90	90	90
β/°	90.347(10)	90.0460(10)	94.9880(10)
γ/°	90	90	90
V/Å^3	13424(3)	14379.5(3)	5725.54(9)
Z	4	4	4
Unique/*I* > 2σ*I*	15084/ 9954	28385/21305	11127/9398
R_{int}	0.0755	0.0552	0.0138
Parameters	961	1790	746
R_1 (all data/ *I* > 2σ*I*)	0.1473/ 0.1071	0.0985/0.0792	0.0661/0.0576
wR_2 (all data/ *I* > 2σ*I*)	0.2808/0.2583	0.2437/0.2183	0.1774/0.1673
GooF	1.069	1.014	1.063
Residual max/min	0.375/-0.685	0.696/-0.466	0.382/-0.344
T/K	150.0(1)	150.0(1)	240.0(1)
Radiation, **λ** (Å)	Cu Kα, (1.54184)	Cu Kα, (1.54184)	Cu Kα, (1.54184)
CCDC number	2124998	2124999	2125000

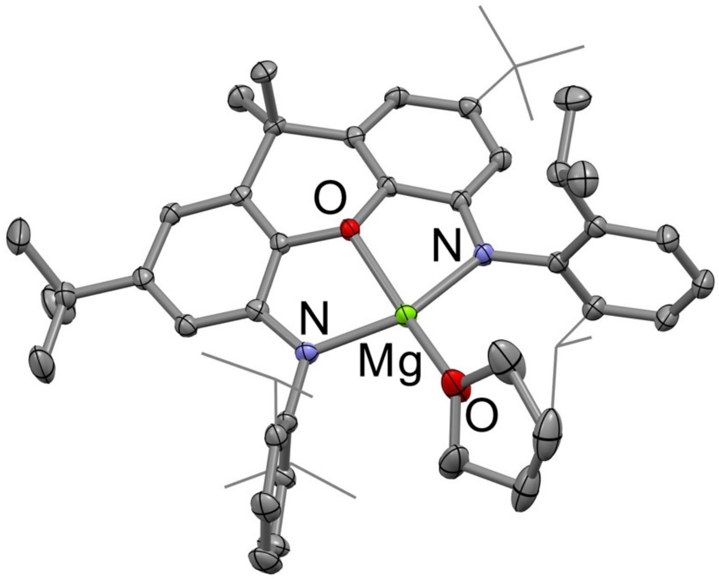

Figure S20. Molecular structure of (**NON**)Mg(THF) as determined by X-ray crystallography. Non-coordinating solvent molecules and hydrogen atoms have been removed, and selected groups of the **NON** ligand are shown in wireframe for clarity. Displacement ellipsoids set at the 50% probability level.

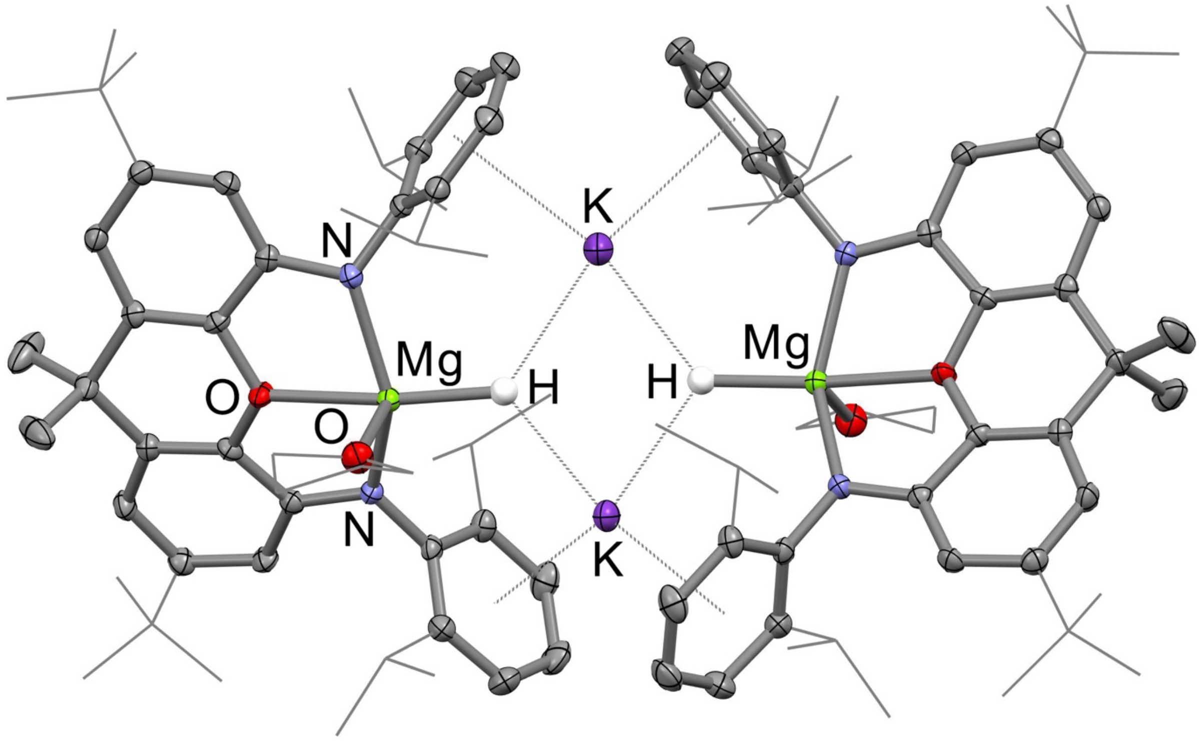

Figure S21. Molecular structure of **1-Mg** as determined by X-ray crystallography. Non-coordinating solvent molecules and most hydrogen atoms have been removed, and selected groups of the **NON** and THF ligands are shown in wireframe for clarity. Displacement ellipsoids set at the 50% probability level.

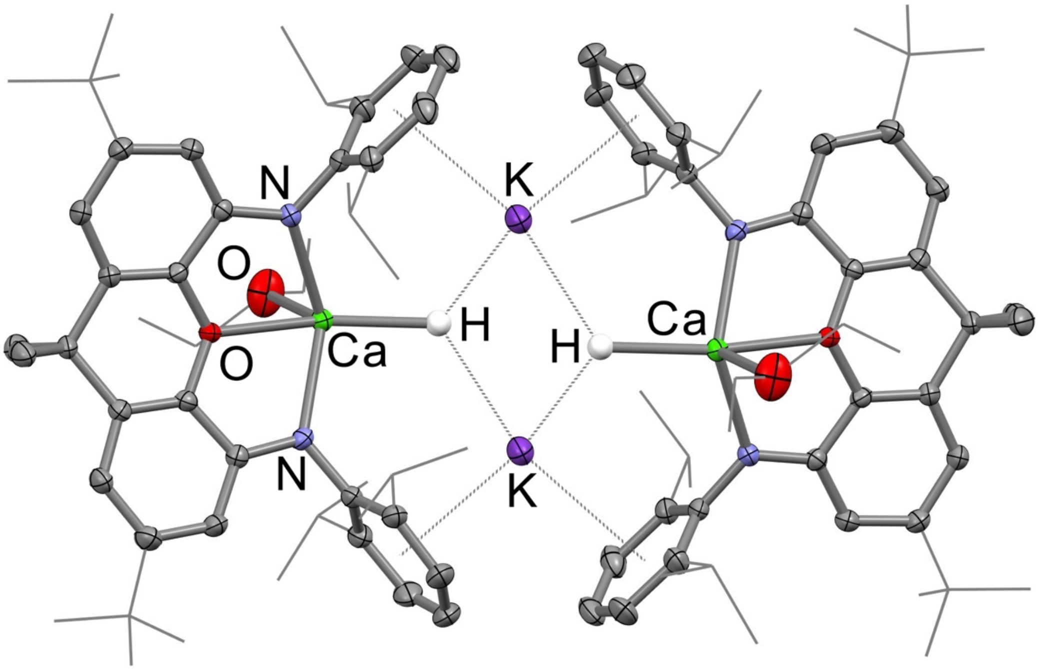

Figure S22. Molecular structure of **1-Ca** as determined by X-ray crystallography. Non-coordinating solvent molecules and most hydrogen atoms have been removed, and selected groups of the **NON** and diethyl ether ligands are shown in wireframe for clarity. Displacement ellipsoids set at the 50% probability level.

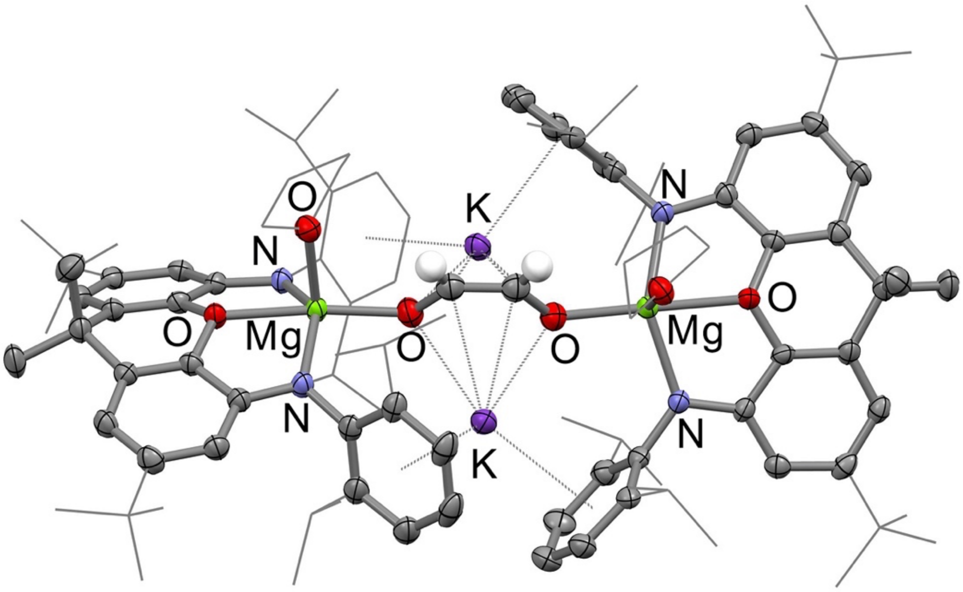

Figure S23. Molecular structure of **2-Mg** as determined by X-ray crystallography. Non-coordinating solvent molecules and most hydrogen atoms have been removed, and selected groups of the **NON** and THF ligands are shown in wireframe for clarity. Displacement ellipsoids set at the 50% probability level.

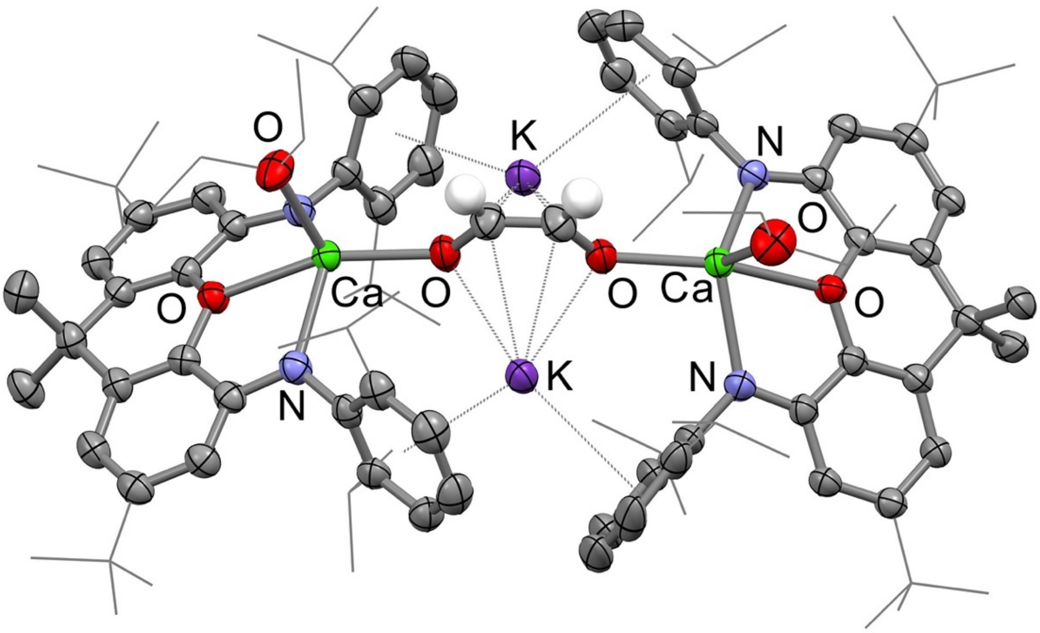

Figure S24. Molecular structure of **2-Ca** as determined by X-ray crystallography. Non-coordinating solvent molecules and most hydrogen atoms have been removed, and selected groups of the **NON** and diethyl ether ligands are shown in wireframe for clarity. Displacement ellipsoids set at the 50% probability level.

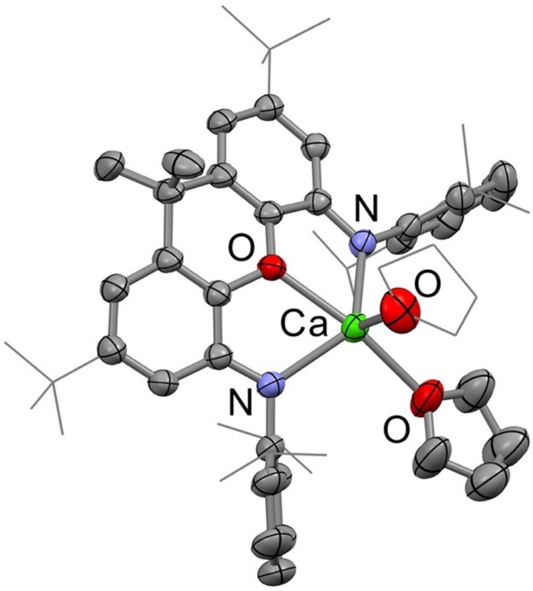

Figure S25. Molecular structure of (**NON**)Ca(THF)$_2$ as determined by X-ray crystallography. Non-coordinating solvent molecules and hydrogen atoms have been removed, and selected groups of the **NON** and THF ligand are shown in wireframe for clarity. Displacement ellipsoids set at the 50% probability level.

Neutron Laue diffraction experiment details

The structures of compounds **1-Mg** and **1-Ca** were investigated using single-crystal neutron Laue diffraction. The unit-cells employed were as for the X-ray determination as these cannot be reliably determined using Laue neutron diffraction. Care was taken to ensure that the temperature of the experiment was matched to the X-ray experiment using an Oxford Cryosystems COBRA™ cryostream which also served to protect the crystal from moisture and oxygen, the sample was handled immersed in argon to ensure compound stability while the crystal was transferred to the coldstream.

Neutron Laue data for both compounds were collected on the KOALA instrument at ANSTO. Data for **1-Mg** were collected from a colourless single-crystal (0.4 x 0.3 x 0.3 mm) mounted to the phi axis of the KOALA diffractometer which stands at an end guide position of TG3 - a supermirror thermal neutron guide at the OPAL nuclear reactor at ANSTO. A total 9 Laue diffraction images were collected in a single run (15000s exposures) with 17° rotation of the crystal around the phi axis occurring between exposures. A total of 17005 reflections from neutrons of wavelengths between 0.85 and 1.70 Å, covering the full sphere of reciprocal space to a maximum resolution of 1.35 Å were reduced [L4R(int) = 7.0(7.3) for 4σ observations] to yield 1660 independent reflections; 619 with $I > 4\sigma(I)$. Data reductions were by means of the LAUEG suite.[S9,S10]

Data for **1-Ca** were collected from a colourless single-crystal (4.0 x 1.5 x 1.4 mm) mounted to the phi axis of the KOALA (as above). A total 48 Laue diffraction images (1000s per exposure) were collected in 3 runs (run 1, 19 images; run 2, 19 images, run 3, 10 images) with 17° rotation of the crystal around the phi axis occurring between exposures. A total of 249419 reflections from neutrons of wavelengths between 0.85 and 1.70 Å covering the full sphere of reciprocal space to a maximum resolution of 0.95 Å were reduced [L4R(int) = 7.7(5.5) for 4σ observations] to yield 32912 independent reflections; 21023 with $I > 4\sigma(I)$. Data reductions were by means of the LAUEG suite.[S9,S10]

The refined structure model CIFs have been deposited at the Cambridge Crystallographic Data Centre (2125001-2125002). These can be obtained free-of-charge via www.ccdc.cam.ac.uk/data_request/cif, by emailing data_request@ccdc.cam.ac.uk, or by contacting The Cambridge Crystallographic Data Centre, 12 Union Road, Cambridge CB2 1EZ, UK; fax: +44 1223 336033.

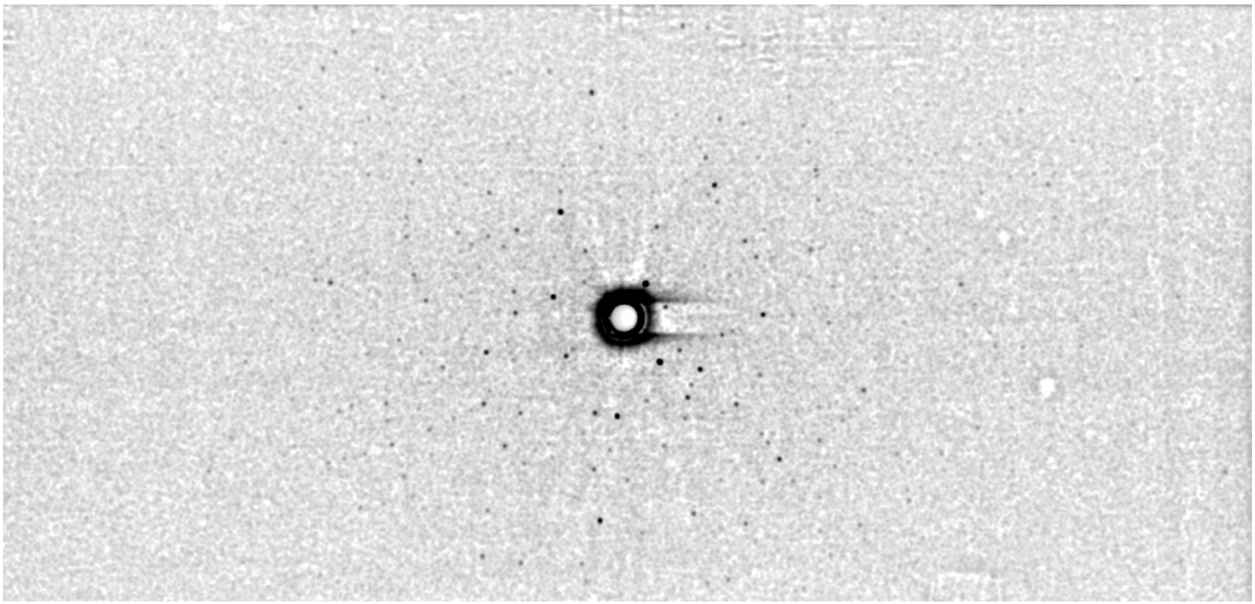

Figure S26. A representative Laue neutron diffraction image from a single-crystal of **1-Mg** from which data have been extracted for refinement of the neutron diffraction study reported here.

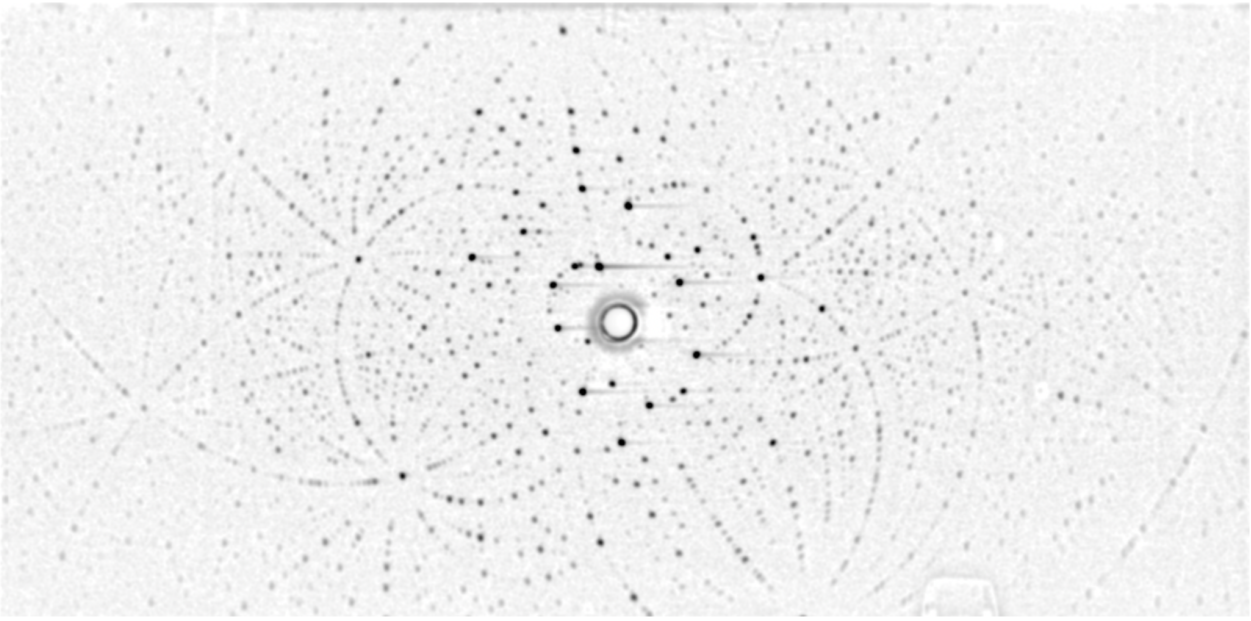

Figure S27. A representative Laue neutron diffraction image from a single-crystal of **1-Ca** from which data have been extracted for refinement of the neutron diffraction study reported here.

Computational details

The geometry optimizations of **1-Mg** and **1-Ca** were performed with the Gaussian16 (Revision C.01) programme[S11] using the PBE1PBE hybrid exchange functional[S12] and Def2-SVP basis sets.[S13] In addition, Grimme's empirical dispersion correction with Becke-Johnson damping (GD3BJ)[S14] was used as well as an ultrafine integration grid. Full analytical frequency calculations were performed for the optimized structures to ensure the nature of the stationary points found (minima, no imaginary frequencies). NBO analyses were done with NBO3.1 as implemented in Gaussian16 programme package.[S15] The QTAIM analyses[S16] were performed for the optimized anionic monomer structures (PBE1PBE-D3BJ/Def2-TZVP, ultrafine integration grid) using ADF2021.102 programme package.[S17] These calculations utilized the PBE1PBE functional and TZ2P basis sets[S18] for all atoms with empirical dispersion correction (GD3BJ) and good numerical quality.

The PBE1PBE-D3BJ/Def2-SVP optimised gas phase structures and calculated bond parameters for **1-Mg** and **1-Ca** are in good agreement with those experimentally observed values. The M–H bond distances were found to be 1.839 (**1-Mg**) and 2.170 Å (**1-Ca**). Inspection of the calculated frontier molecular orbitals reveals that the M–H σ-interaction is not the HOMO, but the HOMO-10 for **1-Mg** (Figure S18) and HOMO-7 for **1-Ca** (Figure S19). Further bonding analyses confirm that the M–H interaction can be described as ionic in nature: the calculated Wiberg bond indices (WBIs) are 0.1980 for **1-Mg** and 0.1491 for **1-Ca**. The quantum theory of atoms in molecules (QTAIM) calculations were performed for the optimised anionic monomeric structures, which returned significant charge difference between the M and H atoms (**1-Mg** 1.682 and H –0.780; **1-Ca** 1.582 and H –0.776;). Consistently, a bond critical point can be found between M and H, albeit with relatively low electron density ρ(r) (see SI) and positive Laplacian $\nabla^2\rho(r)$.

Table S3. Optimised bond parameters, relevant (uncorrected) IR frequencies, and FMO energies.

	**1-Ca**	**1-Mg**	**1-Ca, monomer**	**1-Mg, monomer**
**M-H (Å)**	2.170	1.839	2.127	1.785
**H-K (Å)**	2.633, 2.624	2.611, 2.564	-	-
**M-M (Å)**	7.075	6.856	-	-
**O-M-H (°)**	169.83	171.94	175.20	175.47
**$\nu_{M\text{-}H}$ (cm^{-1})**	1054, 1087	1267, 1292	1090	1370
**HOMO (eV)**	-4.578	-4.592	-2.149	-2.043
**LUMO (eV)**	-0.382	-0.488	2.432	2.546
**gap (eV)**	4.196	4.104	4.581	4.589

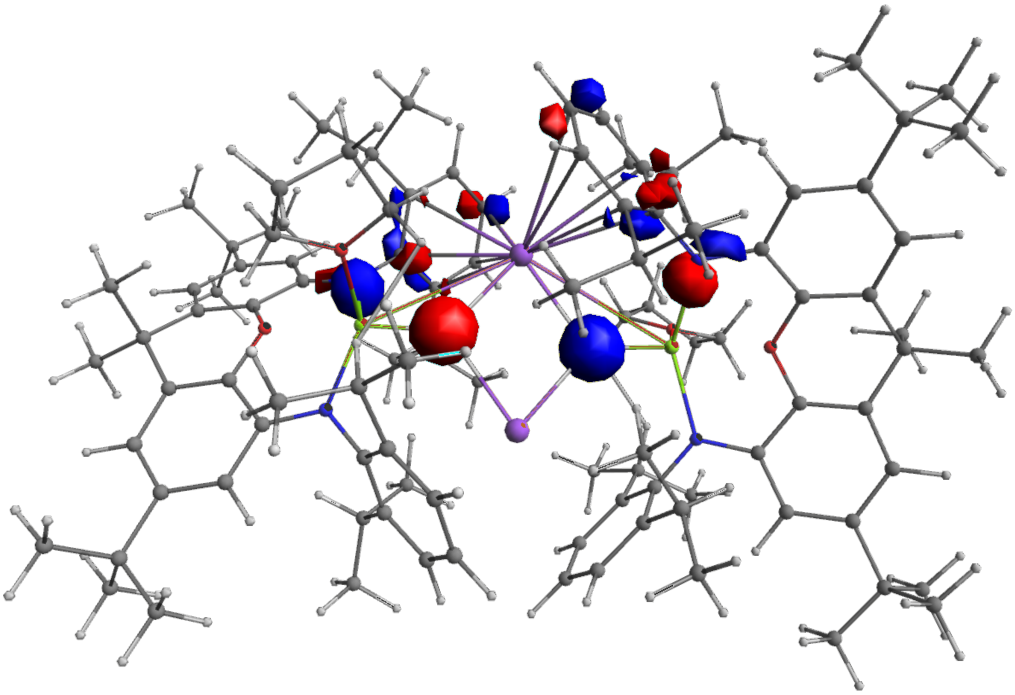

Figure S28. Plot of HOMO-10 (-6.441 eV) of **1-Mg** (isosurface value set at ±0.05 a.u.).

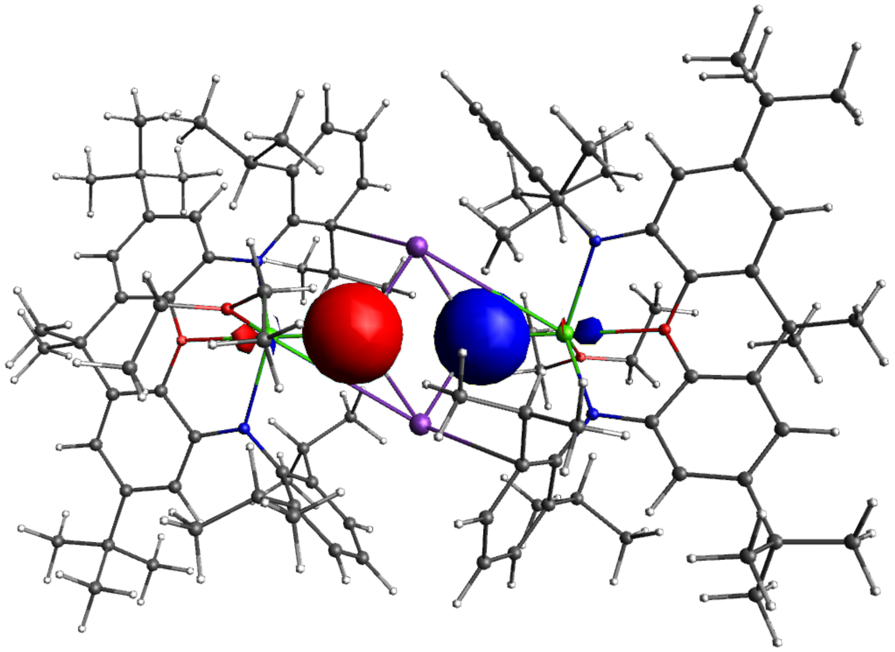

Figure S29. Plot of HOMO-7 (-6.220 eV) of **1-Ca** (isosurface value set at ±0.05 a.u.).

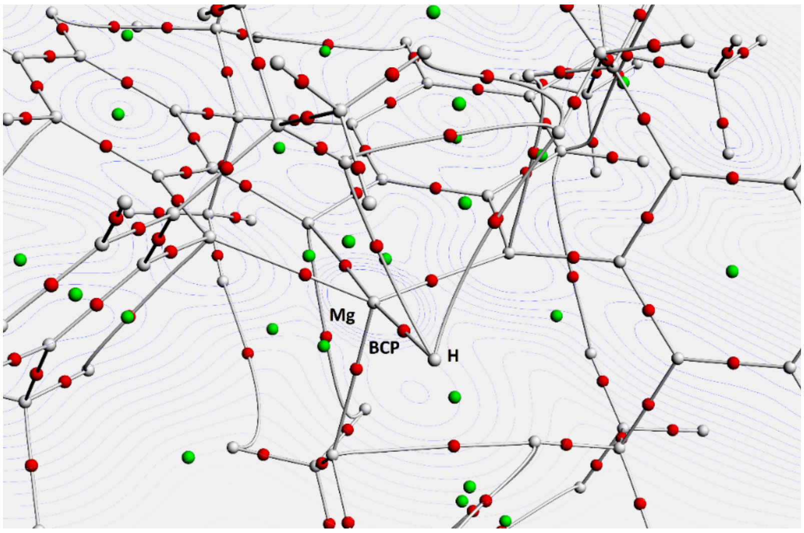

Figure S30. Contour plot of anionic **1-Mg** monomer. Red spheres are bond critical points.

Table S4. QTAIM derived atomic and BCP data for anionic monomer of **1-Mg**.

Atom/BCP	q(A)	ρ(r)	$\nabla^2\rho(r)$
**Mg**	1.682		
**H**	-0.780		
**Mg-H**		0.04708	0.14740

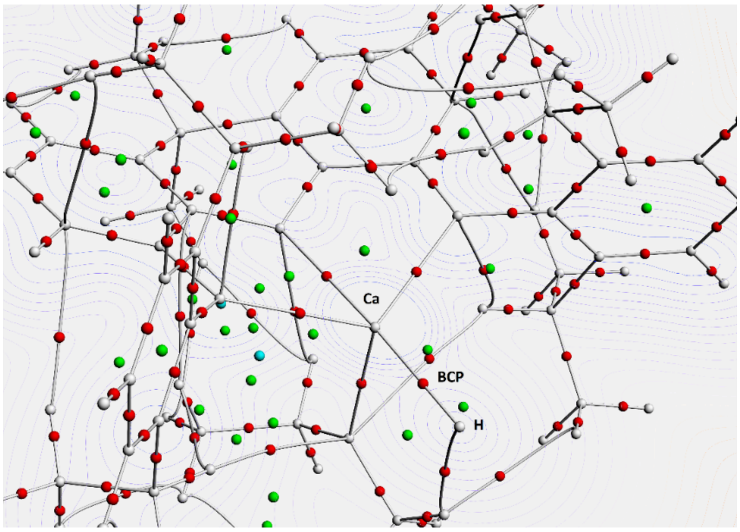

Figure S31. Contour plot of anionic **1-Ca** monomer. Red spheres are bond critical points.

Table S5. QTAIM derived atomic and BCP data for anionic monomer of **1-Ca**.

Atom/BCP	q(A)	ρ(r)	$\nabla^2\rho(r)$
**Ca**	1.582		
**H**	-0.776		
**Ca-H**		0.04029	0.07681

xyz-coordinates of the optimised structures

1-Mg

256

K	0.00002	1.87891	0.00002
Mg	-3.35337	-0.23140	-0.71033
H	-1.51854	-0.15574	-0.60940
O	-5.50130	-0.19682	-0.55181
N	-3.90295	1.75900	-1.16772
C	-6.11813	1.02114	-0.74602
K	-0.00002	-2.13033	0.00006
O	-3.51148	-1.23405	-2.55351
N	-3.82709	-1.75580	0.67832
C	-5.23385	2.06400	-1.08397
C	-5.83403	3.32434	-1.29662
H	-5.18254	4.15226	-1.57404
C	-7.20738	3.52586	-1.12770
C	-8.02000	2.44729	-0.74890
H	-9.08899	2.60184	-0.60449
C	-7.48089	1.16868	-0.55633
C	-8.29540	-0.07989	-0.21396
C	-7.42661	-1.00367	0.64160
C	-7.91184	-1.86978	1.63057
H	-8.97592	-1.86742	1.86567

C	-7.05109	-2.73884	2.31644
C	-5.68594	-2.73460	2.01188
H	-4.99977	-3.39297	2.54319
C	-5.14309	-1.87605	1.03202
C	-6.06928	-1.04239	0.37733
C	-7.62854	-3.67195	3.38718
C	-8.29372	-2.83218	4.48687
H	-8.71740	-3.48251	5.26956
H	-9.10850	-2.21050	4.08788
H	-7.56143	-2.15880	4.95860
C	-8.67116	-4.59739	2.74381
H	-8.21324	-5.20636	1.94888
H	-9.49741	-4.02798	2.29355
H	-9.10130	-5.27985	3.49502
C	-6.55317	-4.54427	4.03709
H	-7.01151	-5.19697	4.79611
H	-5.78397	-3.93805	4.53941
H	-6.05093	-5.19025	3.30089
C	-8.61591	-0.81530	-1.53137
H	-9.21537	-0.17237	-2.19451
H	-9.17573	-1.74149	-1.32829
H	-7.69072	-1.08503	-2.06136
C	-9.60186	0.27572	0.48953
H	-9.41736	0.80355	1.43641
H	-10.18799	-0.62917	0.70317
H	-10.22764	0.91439	-0.14962
C	-7.84471	4.90542	-1.33006
C	-6.82339	5.95883	-1.76181
H	-6.33850	5.69176	-2.71298
H	-6.03573	6.09839	-1.00578
H	-7.32505	6.92875	-1.90225
C	-8.92600	4.81431	-2.41609
H	-9.39819	5.79724	-2.57662
H	-9.71799	4.10146	-2.14381
H	-8.49122	4.48203	-3.37142
C	-8.47860	5.36499	-0.00918
H	-7.71765	5.43390	0.78363
H	-9.25492	4.66568	0.33435
H	-8.94465	6.35705	-0.12615
C	-2.99488	2.79666	-1.39913
C	-2.32433	2.87934	-2.64966
C	-1.34760	3.86230	-2.84308
H	-0.82375	3.92233	-3.79942
C	-1.05013	4.78938	-1.84646
H	-0.29679	5.56127	-2.02133
C	-1.73533	4.73626	-0.63452
H	-1.50916	5.47259	0.13903
C	-2.69784	3.75063	-0.38721

C	-3.38141	3.65995	0.96539
H	-4.36289	3.19530	0.80099
C	-3.62927	5.01456	1.61829
H	-2.69353	5.51227	1.92056
H	-4.23783	4.89181	2.52721
H	-4.16941	5.69121	0.93891
C	-2.60078	2.73365	1.89736
H	-2.47555	1.73737	1.44437
H	-3.13376	2.58570	2.84961
H	-1.60672	3.14970	2.13867
C	-2.73987	1.95015	-3.77159
H	-2.92877	0.97376	-3.30102
C	-1.68693	1.76753	-4.85670
H	-1.53877	2.68699	-5.44550
H	-2.00179	0.98626	-5.56666
H	-0.71347	1.47283	-4.44105
C	-4.06310	2.41812	-4.38446
H	-4.85608	2.49548	-3.62820
H	-4.40129	1.72018	-5.16822
H	-3.94250	3.40930	-4.85079
C	-2.92400	-2.62857	1.28678
C	-2.21899	-2.23180	2.45498
C	-1.32553	-3.12429	3.05961
H	-0.80342	-2.82491	3.97284
C	-1.10714	-4.39488	2.53085
H	-0.42587	-5.08995	3.02702
C	-1.77304	-4.77618	1.36681
H	-1.59046	-5.76935	0.94856
C	-2.67438	-3.91455	0.72963
C	-3.39577	-4.35207	-0.53067
H	-3.74659	-3.42371	-1.01033
C	-4.62885	-5.20097	-0.21246
H	-4.33901	-6.13029	0.30543
H	-5.15476	-5.48201	-1.13902
H	-5.33607	-4.65692	0.42782
C	-2.48309	-5.09052	-1.50667
H	-1.55664	-4.53417	-1.71666
H	-3.00051	-5.26017	-2.46403
H	-2.18658	-6.08122	-1.12669
C	-2.46856	-0.86790	3.06010
H	-2.97930	-0.28463	2.27913
C	-3.41941	-0.95236	4.25505
H	-4.37635	-1.40964	3.96648
H	-3.63036	0.05241	4.65462
H	-2.98058	-1.55445	5.06810
C	-1.17964	-0.14474	3.43214
H	-0.65149	-0.64301	4.26108
H	-1.39782	0.88102	3.76458

H	-0.47908	-0.08306	2.58244
C	-4.72165	-1.26070	-3.32595
H	-5.21404	-0.28273	-3.23318
H	-5.38684	-2.03126	-2.90224
C	-4.27357	-1.59725	-4.73662
H	-5.06109	-2.09488	-5.31904
H	-3.98248	-0.68022	-5.27272
C	-3.05239	-2.47513	-4.48401
H	-2.36797	-2.53767	-5.34157
H	-3.36123	-3.49896	-4.21972
C	-2.41747	-1.79129	-3.28661
H	-1.86704	-2.48050	-2.63120
H	-1.73696	-0.97747	-3.58360
Mg	3.35336	-0.23144	0.71036
H	1.51854	-0.15576	0.60945
O	5.50131	-0.19689	0.55180
N	3.90299	1.75895	1.16773
C	6.11815	1.02106	0.74599
O	3.51146	-1.23408	2.55356
N	3.82704	-1.75586	-0.67829
C	5.23389	2.06393	1.08395
C	5.83409	3.32427	1.29659
H	5.18262	4.15219	1.57402
C	7.20744	3.52578	1.12764
C	8.02004	2.44719	0.74882
H	9.08903	2.60174	0.60439
C	7.48091	1.16859	0.55626
C	8.29540	-0.07999	0.21387
C	7.42658	-1.00376	-0.64166
C	7.91177	-1.86986	-1.63066
H	8.97585	-1.86751	-1.86579
C	7.05099	-2.73890	-2.31652
C	5.68585	-2.73465	-2.01192
H	4.99966	-3.39301	-2.54322
C	5.14304	-1.87612	-1.03203
C	6.06925	-1.04246	-0.37736
C	7.62840	-3.67200	-3.38729
C	8.29355	-2.83222	-4.48698
H	8.71721	-3.48255	-5.26969
H	9.10836	-2.21056	-4.08801
H	7.56126	-2.15883	-4.95868
C	8.67103	-4.59747	-2.74397
H	8.21313	-5.20644	-1.94902
H	9.49729	-4.02807	-2.29372
H	9.10114	-5.27992	-3.49519
C	6.55300	-4.54430	-4.03718
H	7.01131	-5.19699	-4.79623
H	5.78379	-3.93806	-4.53946

H	6.05078	-5.19028	-3.30097
C	8.61594	-0.81540	1.53126
H	9.21542	-0.17248	2.19440
H	9.17575	-1.74159	1.32817
H	7.69076	-1.08513	2.06129
C	9.60184	0.27562	-0.48965
H	9.41732	0.80345	-1.43653
H	10.18796	-0.62928	-0.70332
H	10.22765	0.91428	0.14948
C	7.84479	4.90532	1.32997
C	6.82349	5.95875	1.76174
H	6.33860	5.69169	2.71291
H	6.03581	6.09832	1.00571
H	7.32515	6.92867	1.90216
C	8.92609	4.81422	2.41599
H	9.39829	5.79714	2.57651
H	9.71808	4.10136	2.14371
H	8.49132	4.48195	3.37133
C	8.47866	5.36488	0.00908
H	7.71770	5.43380	-0.78371
H	9.25496	4.66555	-0.33445
H	8.94472	6.35694	0.12604
C	2.99494	2.79664	1.39915
C	2.32447	2.87936	2.64972
C	1.34781	3.86239	2.84320
H	0.82402	3.92244	3.79956
C	1.05036	4.78950	1.84660
H	0.29709	5.56144	2.02152
C	1.73550	4.73634	0.63463
H	1.50934	5.47269	-0.13890
C	2.69792	3.75064	0.38725
C	3.38141	3.65993	-0.96538
H	4.36282	3.19510	-0.80106
C	3.62947	5.01453	-1.61821
H	2.69379	5.51242	-1.92042
H	4.23797	4.89173	-2.52717
H	4.16975	5.69105	-0.93882
C	2.60057	2.73382	-1.89738
H	2.47512	1.73755	-1.44441
H	3.13351	2.58578	-2.84964
H	1.60660	3.15009	-2.13867
C	2.74001	1.95014	3.77163
H	2.92884	0.97375	3.30104
C	1.68712	1.76757	4.85679
H	1.53905	2.68703	5.44560
H	2.00198	0.98628	5.56672
H	0.71363	1.47293	4.44119
C	4.06330	2.41803	4.38443

H	4.85625	2.49535	3.62812
H	4.40150	1.72007	5.16816
H	3.94278	3.40922	4.85077
C	2.92393	-2.62861	-1.28674
C	2.21893	-2.23180	-2.45492
C	1.32545	-3.12427	-3.05957
H	0.80334	-2.82486	-3.97279
C	1.10703	-4.39486	-2.53083
H	0.42575	-5.08991	-3.02700
C	1.77293	-4.77619	-1.36680
H	1.59032	-5.76937	-0.94856
C	2.67429	-3.91459	-0.72961
C	3.39566	-4.35217	0.53070
H	3.74650	-3.42383	1.01037
C	4.62871	-5.20110	0.21248
H	4.33884	-6.13041	-0.30543
H	5.15459	-5.48219	1.13904
H	5.33596	-4.65708	-0.42778
C	2.48294	-5.09060	1.50667
H	1.55650	-4.53422	1.71665
H	3.00034	-5.26029	2.46402
H	2.18640	-6.08128	1.12665
C	2.46857	-0.86790	-3.06001
H	2.97936	-0.28471	-2.27902
C	3.41941	-0.95238	-4.25497
H	4.37631	-1.40974	-3.96643
H	3.63043	0.05240	-4.65449
H	2.98053	-1.55439	-5.06804
C	1.17971	-0.14460	-3.43198
H	0.65151	-0.64277	-4.26095
H	1.39797	0.88116	-3.76435
H	0.47916	-0.08293	-2.58227
C	4.72163	-1.26074	3.32599
H	5.21404	-0.28279	3.23320
H	5.38680	-2.03132	2.90229
C	4.27354	-1.59725	4.73667
H	5.06106	-2.09489	5.31910
H	3.98249	-0.68021	5.27276
C	3.05235	-2.47511	4.48409
H	2.36793	-2.53761	5.34165
H	3.36115	-3.49895	4.21981
C	2.41744	-1.79127	3.28667
H	1.86699	-2.48048	2.63128
H	1.73695	-0.97743	3.58365

1-Ca

260

C	-3.77535	2.70195	4.52205

C	-2.64680	2.07193	3.70978
C	-3.19157	1.24261	2.54701
C	-1.64562	3.09618	3.21403
C	-0.26599	2.98270	3.54346
C	0.65466	3.92093	2.99653
C	0.18060	4.94778	2.17376
C	-1.17516	5.07031	1.87179
C	-2.07519	4.14103	2.38715
N	0.18921	1.93208	4.33842
C	-0.06400	1.98520	5.68257
C	-0.54087	3.10397	6.39282
C	-0.75912	3.07913	7.77676
C	-0.50499	1.90081	8.48356
C	-0.03049	0.75352	7.82696
C	0.17264	0.82671	6.46109
O	0.59445	-0.28348	5.75045
C	0.21225	-1.52101	6.24014
C	0.02002	-1.71112	7.59666
C	0.34408	-0.54822	8.53371
C	-0.39259	-2.98064	8.03369
C	-0.58970	-4.02084	7.12250
C	-0.37886	-3.78120	5.75854
C	0.01254	-2.52279	5.25928
N	0.19513	-2.20389	3.93883
C	0.01402	-3.24220	3.02328
C	1.09314	-4.10995	2.69578
C	0.86864	-5.19960	1.84808
C	-0.38750	-5.44132	1.30036
C	-1.42839	-4.55354	1.55954
C	-1.24831	-3.44545	2.39655
C	-1.04324	-5.41806	7.55934
C	-1.21324	-5.52260	9.07524
C	-0.36517	-0.68761	9.87754
C	1.86825	-0.53991	8.77139
C	-1.24487	4.35756	8.46875
C	-2.56428	4.82056	7.83524
C	2.47243	-3.87575	3.27807
C	2.80207	-4.88199	4.38114
C	-2.40035	-2.49746	2.66847
C	-3.29929	-3.01969	3.79049
C	2.12422	3.82031	3.35564
C	3.05655	4.39230	2.29399
C	-1.48128	4.15079	9.96523
C	-0.18346	5.45398	8.29418
C	-2.39237	-5.73995	6.89984
C	0.00049	-6.45466	7.11928
C	-3.21615	-2.18144	1.41586
C	3.55300	-3.86366	2.19782

C	2.39369	4.45712	4.72062
Ca	0.97174	-0.06347	3.40087
O	3.32449	-0.16691	3.59618
C	3.94623	-0.24334	4.87955
C	3.88116	1.07469	5.61595
C	4.16930	-0.22693	2.45471
C	4.98349	1.02720	2.23675
K	0.31163	-2.09470	-0.12619
C	0.38750	5.44132	-1.30036
C	-0.86864	5.19960	-1.84808
C	-1.09314	4.10995	-2.69578
C	-0.01402	3.24220	-3.02328
C	1.24831	3.44545	-2.39655
C	1.42839	4.55354	-1.55954
N	-0.19513	2.20389	-3.93883
C	-0.01254	2.52279	-5.25928
C	-0.21225	1.52101	-6.24014
C	-0.02002	1.71112	-7.59666
C	0.39259	2.98064	-8.03369
C	0.58970	4.02084	-7.12250
C	0.37886	3.78120	-5.75854
C	-0.34408	0.54822	-8.53371
C	0.03049	-0.75352	-7.82696
C	-0.17264	-0.82671	-6.46109
O	-0.59445	0.28348	-5.75045
C	0.06400	-1.98520	-5.68257
C	0.54087	-3.10397	-6.39282
C	0.75912	-3.07913	-7.77676
C	0.50499	-1.90081	-8.48356
C	1.04324	5.41806	-7.55934
C	1.21324	5.52260	-9.07524
C	0.36517	0.68761	-9.87754
C	-1.86825	0.53991	-8.77139
C	1.24487	-4.35756	-8.46875
C	2.56428	-4.82056	-7.83524
N	-0.18921	-1.93208	-4.33842
C	0.26599	-2.98270	-3.54346
C	1.64562	-3.09618	-3.21403
C	2.07519	-4.14103	-2.38715
C	1.17516	-5.07031	-1.87179
C	-0.18060	-4.94778	-2.17376
C	-0.65466	-3.92093	-2.99653
C	-2.47243	3.87575	-3.27807
C	-2.80207	4.88199	-4.38114
C	2.40035	2.49746	-2.66847
C	3.29929	3.01969	-3.79049
C	2.64680	-2.07193	-3.70978
C	3.77535	-2.70195	-4.52205

C	-2.12422	-3.82031	-3.35564
C	-3.05655	-4.39230	-2.29399
C	1.48128	-4.15079	-9.96523
C	0.18346	-5.45398	-8.29418
C	2.39237	5.73995	-6.89984
C	-0.00049	6.45466	-7.11928
C	3.21615	2.18144	-1.41586
C	-3.55300	3.86366	-2.19782
C	-2.39369	-4.45712	-4.72062
C	3.19157	-1.24261	-2.54701
Ca	-0.97174	0.06347	-3.40087
O	-3.32449	0.16691	-3.59618
C	-3.94623	0.24334	-4.87955
C	-3.88116	-1.07469	-5.61595
C	-4.16930	0.22693	-2.45471
C	-4.98349	-1.02720	-2.23675
K	-0.31163	2.09470	0.12619
H	0.93682	0.07058	1.23539
H	-0.54495	-4.58250	5.03597
H	-0.55261	-3.14682	9.09594
H	-0.66910	1.86267	9.55747
H	-0.74121	4.01446	5.82413
H	-2.24226	3.37815	10.15331
H	-1.83596	5.08832	10.42065
H	-0.55805	3.85692	10.48717
H	-0.50517	6.38871	8.78200
H	0.00100	5.67252	7.23189
H	0.77339	5.14401	8.74200
H	-3.33827	4.04346	7.93109
H	-2.44565	5.04748	6.76557
H	-2.93146	5.73389	8.33070
H	-0.06074	-1.61378	10.38477
H	-1.45813	-0.69906	9.75696
H	-0.09707	0.14179	10.54695
H	2.18739	-1.47800	9.25163
H	2.15308	0.30701	9.41506
H	2.40896	-0.44169	7.81901
H	-2.74334	-6.74036	7.20152
H	-2.32160	-5.72443	5.80225
H	-3.15592	-5.00399	7.19568
H	0.98056	-6.23847	7.57225
H	0.13096	-6.46245	6.02712
H	-0.30466	-7.46769	7.42828
H	-0.27009	-5.31995	9.60525
H	-1.53663	-6.53960	9.34608
H	-1.97402	-4.82059	9.44885
H	1.69282	-5.88161	1.62111
H	-0.55257	-6.31188	0.66212

H	-2.40715	-4.73423	1.10831
H	-1.94420	-1.56258	3.03394
H	-2.60099	-1.74408	0.61105
H	-4.00722	-1.45307	1.64735
H	-3.71604	-3.07508	1.01026
H	-3.77594	-3.97054	3.49987
H	-4.09792	-2.29589	4.01932
H	-2.72373	-3.19446	4.71008
H	2.44059	-2.87737	3.74155
H	3.66001	-4.84535	1.70956
H	4.53366	-3.61081	2.63133
H	3.33214	-3.12533	1.40980
H	2.05198	-4.84484	5.18355
H	3.78778	-4.66741	4.82449
H	2.82929	-5.91042	3.98468
H	-3.13804	4.22925	2.14214
H	-1.52522	5.88210	1.23070
H	0.88405	5.67291	1.76098
H	2.34229	2.74413	3.46056
H	2.16123	5.53424	4.69720
H	3.45297	4.34625	5.00316
H	1.77939	3.99495	5.50580
H	2.95282	5.48465	2.19690
H	2.87195	3.94826	1.30324
H	4.10625	4.19894	2.56334
H	-2.10249	1.38730	4.37573
H	-3.73177	1.86914	1.81732
H	-3.89265	0.47512	2.90925
H	-2.38428	0.71597	2.01055
H	-3.37352	3.26322	5.37695
H	-4.44767	1.92398	4.91632
H	-4.38409	3.39074	3.91365
H	4.32953	1.90509	2.14083
H	5.56570	0.93292	1.30762
H	5.69264	1.20846	3.05842
H	4.81729	-1.11710	2.53737
H	3.48821	-0.38814	1.60373
H	3.41549	-1.02766	5.44444
H	4.98702	-0.58460	4.74807
H	4.43615	1.86222	5.08639
H	4.31235	0.96358	6.62208
H	2.83934	1.40815	5.73451
H	-0.93682	-0.07058	-1.23539
H	0.54495	4.58250	-5.03597
H	0.55261	3.14682	-9.09594
H	0.66910	-1.86267	-9.55747
H	0.74121	-4.01446	-5.82413
H	2.24226	-3.37815	-10.15331

H	1.83596	-5.08832	-10.42065
H	0.55805	-3.85692	-10.48717
H	0.50517	-6.38871	-8.78200
H	-0.00100	-5.67252	-7.23189
H	-0.77339	-5.14401	-8.74200
H	3.33827	-4.04346	-7.93109
H	2.44565	-5.04748	-6.76557
H	2.93146	-5.73389	-8.33070
H	0.06074	1.61378	-10.38477
H	1.45813	0.69906	-9.75696
H	0.09707	-0.14179	-10.54695
H	-2.18739	1.47800	-9.25163
H	-2.15308	-0.30701	-9.41506
H	-2.40896	0.44169	-7.81901
H	2.74334	6.74036	-7.20152
H	2.32160	5.72443	-5.80225
H	3.15592	5.00399	-7.19568
H	-0.98056	6.23847	-7.57225
H	-0.13096	6.46245	-6.02712
H	0.30466	7.46769	-7.42828
H	0.27009	5.31995	-9.60525
H	1.53663	6.53960	-9.34608
H	1.97402	4.82059	-9.44885
H	-1.69282	5.88161	-1.62111
H	0.55257	6.31188	-0.66212
H	2.40715	4.73423	-1.10831
H	1.94420	1.56258	-3.03394
H	2.60099	1.74408	-0.61105
H	4.00722	1.45307	-1.64735
H	3.71604	3.07508	-1.01026
H	3.77594	3.97054	-3.49987
H	4.09792	2.29589	-4.01932
H	2.72373	3.19446	-4.71008
H	-2.44059	2.87737	-3.74155
H	-3.66001	4.84535	-1.70956
H	-4.53366	3.61081	-2.63133
H	-3.33214	3.12533	-1.40980
H	-2.05198	4.84484	-5.18355
H	-3.78778	4.66741	-4.82449
H	-2.82929	5.91042	-3.98468
H	3.13804	-4.22925	-2.14214
H	1.52522	-5.88210	-1.23070
H	-0.88405	-5.67291	-1.76098
H	-2.34229	-2.74413	-3.46056
H	-2.16123	-5.53424	-4.69720
H	-3.45297	-4.34625	-5.00316
H	-1.77939	-3.99495	-5.50580
H	-2.95282	-5.48465	-2.19690

H	-2.87195	-3.94826	-1.30324
H	-4.10625	-4.19894	-2.56334
H	2.10249	-1.38730	-4.37573
H	3.73177	-1.86914	-1.81732
H	3.89265	-0.47512	-2.90925
H	2.38428	-0.71597	-2.01055
H	3.37352	-3.26322	-5.37695
H	4.44767	-1.92398	-4.91632
H	4.38409	-3.39074	-3.91365
H	-4.32953	-1.90509	-2.14083
H	-5.56570	-0.93292	-1.30762
H	-5.69264	-1.20846	-3.05842
H	-4.81729	1.11710	-2.53737
H	-3.48821	0.38814	-1.60373
H	-3.41549	1.02766	-5.44444
H	-4.98702	0.58460	-4.74807
H	-4.43615	-1.86222	-5.08639
H	-4.31235	-0.96358	-6.62208
H	-2.83934	-1.40815	-5.73451

1-Mg anionic monomer

127

Mg	0.00411	-1.54835	-0.00431
H	0.06162	-3.21041	0.64420
O	-0.03146	0.60746	-0.65862
N	1.99396	-0.79174	0.14575
C	1.14253	1.31055	-0.51038
O	-0.07393	-2.03620	-2.08213
N	-1.96389	-0.81104	0.33110
C	2.22260	0.52600	-0.06909
C	3.43230	1.22421	0.12066
H	4.28516	0.65578	0.46466
C	3.53329	2.59402	-0.09318
C	2.41353	3.31237	-0.51198
H	2.48540	4.38047	-0.66742
C	1.19372	2.66695	-0.72696
C	-0.07103	3.35017	-1.23169
C	-1.27221	2.65728	-0.59986
C	-2.47497	3.29038	-0.27947
H	-2.57247	4.35689	-0.43209
C	-3.54762	2.56051	0.23283
C	-3.41646	1.19082	0.42836
H	-4.22953	0.61291	0.84539
C	-2.22270	0.50365	0.13125
C	-1.18960	1.30139	-0.38935
C	-4.84816	3.29008	0.56675
C	-4.57033	4.39527	1.58980

H	-5.49382	4.92914	1.83793
H	-3.84988	5.12169	1.20959
H	-4.16001	3.97044	2.50908
C	-5.42140	3.91434	-0.70942
H	-5.63078	3.14116	-1.45304
H	-4.72017	4.62268	-1.15485
H	-6.35420	4.44604	-0.49301
C	-5.90526	2.36017	1.15449
H	-6.81011	2.93176	1.38093
H	-5.56044	1.89377	2.08012
H	-6.17822	1.56423	0.45779
C	-0.14968	3.14590	-2.75582
H	0.71623	3.60113	-3.24476
H	-1.06356	3.59745	-3.15193
H	-0.16103	2.08201	-2.99924
C	-0.06166	4.84431	-0.94051
H	-0.00535	5.03982	0.13202
H	-0.96378	5.31691	-1.33338
H	0.78951	5.32441	-1.42707
C	4.84775	3.33844	0.13681
C	5.97681	2.40950	0.57202
H	6.17677	1.64029	-0.17753
H	5.74766	1.90857	1.51530
H	6.89354	2.98919	0.71422
C	5.27908	4.03155	-1.15912
H	6.21895	4.57375	-1.01035
H	4.52617	4.74409	-1.50103
H	5.42769	3.29676	-1.95439
C	4.64990	4.39113	1.23183
H	4.34357	3.91635	2.16721
H	3.87711	5.11156	0.95714
H	5.58081	4.93948	1.41153
C	3.05341	-1.53122	0.69200
C	3.83408	-2.36355	-0.13320
C	4.87712	-3.09498	0.42142
H	5.47806	-3.73752	-0.21303
C	5.16170	-3.02235	1.77490
H	5.97959	-3.59884	2.19394
C	4.38116	-2.22082	2.58881
H	4.58685	-2.17960	3.65420
C	3.32837	-1.47580	2.07098
C	2.45999	-0.65810	2.99757
H	1.76311	-0.09893	2.37113
C	3.26139	0.35188	3.81037
H	3.97682	-0.14262	4.47474
H	2.59453	0.95668	4.43169
H	3.81552	1.02585	3.15335
C	1.63845	-1.57645	3.89796

H	1.06350	-2.28632	3.29903
H	0.94359	-0.99634	4.51274
H	2.28620	-2.14986	4.56867
C	3.53516	-2.42906	-1.61191
H	2.46678	-2.22073	-1.70268
C	3.81255	-3.79285	-2.22795
H	4.88305	-4.01643	-2.26073
H	3.44933	-3.82431	-3.25996
H	3.31930	-4.58947	-1.66604
C	4.27388	-1.33004	-2.37262
H	4.02553	-0.34442	-1.97592
H	4.01519	-1.34843	-3.43711
H	5.35680	-1.46669	-2.28741
C	-3.05096	-1.63972	0.62844
C	-3.16430	-2.22384	1.90799
C	-4.24264	-3.05431	2.18425
H	-4.32899	-3.50541	3.16603
C	-5.21449	-3.31734	1.23190
H	-6.05400	-3.96216	1.46940
C	-5.09138	-2.76267	-0.02879
H	-5.83762	-2.98528	-0.78620
C	-4.01957	-1.93862	-0.35270
C	-3.88967	-1.41834	-1.76755
H	-2.96770	-0.83620	-1.80971
C	-5.04534	-0.50431	-2.16198
H	-5.99979	-1.03919	-2.12988
H	-4.90804	-0.12589	-3.17977
H	-5.10777	0.35238	-1.48993
C	-3.75661	-2.56824	-2.76216
H	-2.93643	-3.23098	-2.48217
H	-3.56728	-2.18659	-3.77083
H	-4.66991	-3.16930	-2.80027
C	-2.12665	-1.90931	2.95911
H	-1.16536	-1.88686	2.43765
C	-2.36444	-0.52834	3.56549
H	-2.38599	0.24340	2.79538
H	-1.57181	-0.27923	4.27790
H	-3.32036	-0.50409	4.09941
C	-2.01361	-2.96361	4.04820
H	-2.90620	-2.99920	4.68131
H	-1.16471	-2.73409	4.69709
H	-1.85280	-3.95569	3.62044
C	0.26436	-1.16535	-3.16631
H	1.05197	-0.48640	-2.83569
H	-0.62329	-0.57660	-3.41868
C	0.68973	-2.07655	-4.30472
H	0.46866	-1.64578	-5.28230
H	1.76433	-2.26852	-4.25141

C	-0.08926	-3.35310	-4.00971
H	0.34593	-4.24148	-4.46979
H	-1.12409	-3.25820	-4.34949
C	-0.03539	-3.40623	-2.49681
H	-0.87059	-3.92168	-2.02435
H	0.88976	-3.86221	-2.13345

1-Ca anionic monomer

129

C	-4.29909	-1.97185	2.94039
C	-3.28040	-1.29686	2.27996
C	-3.13247	-1.45217	0.88549
C	-4.00490	-2.31676	0.19307
C	-5.00773	-2.97763	0.89270
C	-5.16785	-2.80446	2.25726
C	-2.31038	-0.44835	3.06982
C	-1.45143	-1.32119	3.98057
N	-2.09293	-0.79882	0.21071
C	-2.25421	0.52826	-0.02073
C	-3.44166	1.25859	0.16211
C	-3.53574	2.62171	-0.10391
C	-2.41391	3.30390	-0.55635
C	-1.20156	2.63103	-0.75285
C	-1.15220	1.28296	-0.48771
O	0.02072	0.56932	-0.63407
C	1.19782	1.26879	-0.44918
C	1.26761	2.61839	-0.70118
C	0.04841	3.30110	-1.30477
C	2.47605	3.28129	-0.45201
C	3.57754	2.58369	0.02570
C	3.47301	1.21290	0.24391
C	2.28176	0.49579	0.03025
C	-3.86174	-2.48381	-1.30227
C	-4.61945	-1.38514	-2.04513
C	-4.88197	3.31521	0.09479
C	-5.91868	2.67994	-0.83735
C	0.04927	4.80121	-1.03748
C	0.08229	3.08062	-2.82789
C	4.89750	3.27867	0.35426
C	6.03850	2.63547	-0.43833
N	2.09478	-0.82636	0.26875
C	3.22199	-1.55587	0.66802
C	4.16782	-1.99657	-0.28005
C	5.28663	-2.70321	0.14435
C	5.47965	-3.00474	1.48054
C	4.52694	-2.61444	2.40706
C	3.39945	-1.89756	2.02550

C	3.95988	-1.74293	-1.75531
C	3.88560	-3.05740	-2.52645
C	2.37822	-1.46049	3.05014
C	2.21236	-2.44502	4.19863
C	-3.00617	0.65156	3.86246
C	4.87026	4.76679	0.02079
C	5.18207	3.13003	1.85250
C	-5.34277	3.14713	1.54525
C	-4.81863	4.80798	-0.21158
C	5.02629	-0.82600	-2.34424
C	2.68681	-0.05940	3.57365
C	-4.27904	-3.85842	-1.80325
Ca	0.00071	-1.88157	-0.16955
O	0.07326	-2.12304	-2.57264
C	0.25510	-3.43107	-3.10573
C	-0.92835	-3.92109	-3.90586
C	0.06589	-1.05611	-3.51339
C	-1.31923	-0.48185	-3.69845
H	-0.01368	-3.99685	0.05034
H	4.32076	0.66081	0.63189
H	2.54123	4.34298	-0.63277
H	-2.46751	4.36190	-0.76134
H	-4.30506	0.71623	0.52913
H	-4.10427	5.32067	0.43736
H	-5.80250	5.25802	-0.05085
H	-4.53138	4.99331	-1.24954
H	-6.89535	3.15873	-0.70984
H	-6.03267	1.61350	-0.63560
H	-5.61407	2.78993	-1.88128
H	-4.61536	3.58496	2.23345
H	-5.45965	2.09451	1.80817
H	-6.30649	3.64322	1.70069
H	0.93860	5.26669	-1.46546
H	0.02468	5.01530	0.03266
H	-0.81418	5.27535	-1.50708
H	0.98330	3.52910	-3.25565
H	-0.79860	3.52847	-3.29663
H	0.08753	2.01512	-3.05988
H	6.12614	3.61906	2.11531
H	5.24970	2.07961	2.14072
H	4.38171	3.58483	2.44149
H	5.85687	2.71917	-1.51297
H	6.14482	1.57617	-0.19922
H	6.98857	3.12983	-0.21024
H	4.68344	4.93594	-1.04276
H	5.83604	5.21651	0.26860
H	4.10039	5.29247	0.59075
H	6.01673	-3.03285	-0.58903

H	6.35720	-3.55789	1.79768
H	4.66509	-2.87573	3.45027
H	1.42141	-1.39272	2.52402
H	2.02044	-3.45510	3.82987
H	1.37076	-2.14685	4.82840
H	3.09939	-2.47733	4.83837
H	3.65003	-0.05031	4.09427
H	1.91622	0.26783	4.27863
H	2.73449	0.66374	2.75842
H	2.99474	-1.24040	-1.85090
H	4.84107	-3.58950	-2.49601
H	3.64263	-2.87477	-3.57819
H	3.12529	-3.71657	-2.10268
H	5.03241	0.13853	-1.83552
H	4.83721	-0.64813	-3.40759
H	6.02272	-1.26990	-2.25199
H	-4.40598	-1.85227	4.01443
H	-5.95673	-3.32829	2.78631
H	-5.67852	-3.64490	0.36334
H	-2.79969	-2.34893	-1.52765
H	-5.69097	-1.44795	-1.82919
H	-4.48597	-1.48320	-3.12727
H	-4.27159	-0.39547	-1.74565
H	-5.35234	-4.03001	-1.67947
H	-3.74216	-4.65109	-1.27739
H	-4.06663	-3.94953	-2.87229
H	-1.64662	0.03404	2.35064
H	-2.06105	-1.81375	4.74420
H	-0.69315	-0.71897	4.48955
H	-0.94250	-2.10483	3.41419
H	-3.58250	1.29976	3.20064
H	-2.26993	1.26919	4.38509
H	-3.68596	0.23719	4.61346
H	-1.82920	-3.91829	-3.29104
H	-0.74210	-4.94771	-4.23200
H	-1.10742	-3.31415	-4.79693
H	1.17056	-3.43875	-3.70920
H	0.40478	-4.06439	-2.22529
H	0.74571	-0.29051	-3.12871
H	0.47903	-1.41794	-4.46175
H	-2.00762	-1.22469	-4.10440
H	-1.28299	0.36948	-4.38315
H	-1.71915	-0.12998	-2.74594

References

S1 C. A. Cruz, D. J. H. Emslie, L. E. Harrington, J. F. Britten, C. M. Robertson, *Organometallics*, **2007**, *26*, 692.

S2 J. S. McMullen, A. J. Edwards, J. Hicks, *Dalton Trans.*, **2021**, *50*, 8685-8689.

S3 G. Wulff, P. Birnbrich, *Chem. Ber.* **1992**, *125*,473-477.

S4 J. Cosier, A. M. Glazer, *J. Appl. Cryst.* 1986, **19**, 105.

S5 CrysAlisPro v171.41.93a (Rigaku Oxford Diffraction, 2021).

S6 G. M. Sheldrick, *Acta Cryst.*, 2015, **C71**, 3-8.

S7 G. M. Sheldrick, *Acta Cryst.*, 2015, **A71**, 3-8.

S8 O. V. Dolomanov, L. J. Bourhis, R. J. Gildea, J. A. K. Howard, H. Puschmann, *J. Appl. Crystallogr*. **2009**, *42*, 339-341.

S9 R. O. Piltz, *J. Appl. Cryst.*, 2018, **51**, 635.

S10 R. O. Piltz*, J. Appl. Cryst.*, 2018. **51**, 963.

S11 Gaussian 16, Revision C.01, M. J. Frisch, G. W. Trucks, H. B. Schlegel, G. E. Scuseria, M. A. Robb, J. R. Cheeseman, G. Scalmani, V. Barone, G. A. Petersson, H. Nakatsuji, X. Li, M. Caricato, A. V. Marenich, J. Bloino, B. G. Janesko, R. Gomperts, B. Mennucci, H. P. Hratchian, J. V. Ortiz, A. F. Izmaylov, J. L. Sonnenberg, D. Williams-Young, F. Ding, F. Lipparini, F. Egidi, J. Goings, B. Peng, A. Petrone, T. Henderson, D. Ranasinghe, V. G. Zakrzewski, J. Gao, N. Rega, G. Zheng, W. Liang, M. Hada, M. Ehara, K. Toyota, R. Fukuda, J. Hasegawa, M. Ishida, T. Nakajima, Y. Honda, O. Kitao, H. Nakai, T. Vreven, K. Throssell, J. A. Montgomery, Jr., J. E. Peralta, F. Ogliaro, M. J. Bearpark, J. J. Heyd, E. N. Brothers, K. N. Kudin, V. N. Staroverov, T. A. Keith, R. Kobayashi, J. Normand, K. Raghavachari, A. P. Rendell, J. C. Burant, S. S. Iyengar, J. Tomasi, M. Cossi, J. M. Millam, M. Klene, C. Adamo, R. Cammi, J. W. Ochterski, R. L. Martin, K. Morokuma, O. Farkas, J. B. Foresman, and D. J. Fox, Gaussian, Inc., Wallingford CT, 2019.

S12 a) J. P. Perdew, K. Burke, and M. Ernzerhof, *Phys. Rev. Lett.*, **1996**, *77*, 3865-68. b) J. P. Perdew, K. Burke, and M. Ernzerhof, *Phys. Rev. Lett.*, **1997**, *78*, 1396. c) C. Adamo and V. Barone, *J. Chem. Phys.*, **1999**, *110*, 6158-69.

S13 a) F. Weigend and R. Ahlrichs, *Phys. Chem. Chem. Phys.*, **2005**, *7,* 3297-305. b) F. Weigend, *Phys. Chem. Chem. Phys.*, **2006**, *8,* 1057-65.

S14 NBO Version 3.1, E. D. Glendening, A. E. Reed, J. E. Carpenter, and F. Weinhold.

S15 S. Grimme, S. Ehrlich and L. Goerigk, *J. Comp. Chem*. **2011**, *32*, 1456-65.

S16 J.I. Rodríguez, R.F.W. Bader, P.W. Ayers, C. Michel, A.W. Götz and C. Bo, *Chemical Physics Letters,* **2009**, *472*, 149, b) J.I. Rodríguez, *Journal of Computational Chemistry*, **2013**, *34*, 681.

S17 a) G. te Velde, F.M. Bickelhaupt, E.J. Baerends, C. Fonseca Guerra, S.J.A. van Gisbergen, J.G. Snijders and T. Ziegler, *Journal of Computational Chemistry,* **2001***, 22*, 931, b) ADF 2021.1, SCM, Theoretical Chemistry, Vrije Universiteit, Amsterdam, The Netherlands, http://www.scm.com.

S18 E. van Lenthe and E.J. Baerends, *Journal of Computational Chemistry*, **2003**, *24*, 1142.
